# Sexual identity-related inequalities in associations between adverse childhood experiences and health in late adolescence–A national cohort study

**DOI:** 10.1371/journal.pone.0312161

**Published:** 2024-12-11

**Authors:** Rahul Chandrasekar, Alexis Karamanos, Annastazia Learoyd, Amal R. Khanolkar

**Affiliations:** 1 University College London Medical School, London, United Kingdom; 2 Department of Population Health Sciences, King’s College London, London, United Kingdom; University of Technology Sydney, AUSTRALIA

## Abstract

This study examined associations between adverse childhood experiences (ACEs) and mental health and health-risk behaviours, including differences by sexual minority (SM) identity. We included 8,686 adolescents (males = 50.7%, SM = 10.3%) from the UK-wide Millennium Cohort Study with data on eight ACEs (e.g., domestic violence, parental psychological distress, bullying) recorded between ages 3–14 and a wide-range of health indicators and health-risk behaviours at age 17. Associations between 1) Sexual identity and ACEs were analysed using multinomial logistic regression and 2) Cumulative ACE scores and all outcomes were analysed using linear/logistic regression (with appropriate interactions assessing differences in ACE-outcome associations by sexual identity and adjusted for sex, ethnicity, and parental income). Results showed SM individuals had a higher prevalence of bullying (33.9% vs. 20.3%) and experiencing ≥3ACEs [Bisexual: RRR 1.87 (95% CI 1.35, 2.57), Gay/Lesbian RRR 2.08 (1.24, 3.48)]. The number of individuals experiencing adverse mental health outcomes increased in relation to greater ACE exposure with evidence for effect-moderation by sexual identity in certain outcomes. For example, among individuals with 0 ACEs, 8% of heterosexual adolescents reported psychological distress vs. 22% in bisexual and 17% in gay/lesbian peers, increasing to 16% in heterosexual vs. 41% in bisexual and 45% in gay/lesbian adolescents with ≥3 ACEs. Similar patterns were observed for other health indicators (e.g., self-harm, suicidality, sleep quality) and health-risk behaviours (e.g., risky sex). Our findings indicate that ACEs are associated (with a gradient) with worse mental health and well-being, and higher risk of some health-risk behaviours. However, the detrimental effect of ACEs on health is worse in SM adolescents compared to heterosexual peers.

## Introduction

Sexual-minority (SM) adolescents have substantially higher risk of poorer mental health including depression/anxiety, self-harm, attempted suicide, and health-risk behaviours such as drug and alcohol misuse; a trend which continues into adulthood [1–6]. The minority stress theory posits that SM individuals are exposed to a chronically stressful social environment by virtue of their sexual identity that their heterosexual counterparts may avoid experiencing, such as systemic discrimination and victimisation [7]. Long-term exposure may lead to psychological distress and mental illness and adoption of health-risk behaviours, possibly as coping mechanisms [8, 9]. Indeed, adolescence is a key life phase not only of biological changes but also discovery of self-identity and psychosocial maturation [10]. Compromise of a healthy mental state and adverse health behaviours in adolescence impacts subsequent health and has psychosocial consequences for accessing higher education, entering the workplace, and effective participation in society as a young adult [11].

Adverse childhood experiences (ACEs), comprising verbal, physical, and sexual maltreatment and events of household dysfunction, affect over half of the population [12–14]. The first major study of the long-term effects of ACEs by Felitti et al. in 1998 included three types of abuse, such as physical abuse, and four markers of household dysfunction, such as domestic violence, and living with household members who were mentally ill [15]. In the twenty-five years since, further household factors, such as parental separation, and community factors, such as neighbourhood violence and discrimination, have been explored [16]. Exposure to ACEs is associated with increased risk of adverse health, including cardiovascular, respiratory, metabolic, and psychiatric illness, and multimorbidity in later life [12, 17, 18]. ACE are also associated with health-risk behaviours like alcohol/drug misuse and sexual risk-taking, but also wider social outcomes such as interpersonal violence and incarceration [12, 19]. A substantial body of evidence now indicates that exposure to an increasing number of ACEs increases risk for a wide range of health outcomes (i.e. a dose-response/gradient relationship) in the short- and long-term [18, 20]. The study by Felitti et al. used a summary score of seven ACEs to create an ‘ACE score’ which became the dominant approach to conceptualising ACEs in health research replicated in hundreds of studies since with a wide range of health and social outcomes due to its simplicity to calculate, implement and understand [21]. ACE scores also have the advantage of measuring adversities experienced by a child in multiple domains [13]. Pathways between ACEs and detrimental health outcomes are hypothesized to implicate allostatic load and dysregulation of multiple body systems such as the immune and autonomic nervous systems, overactivation of the hypothalamic-pituitary-adrenal (HPA) axis, and even compromised somatic growth [22, 23]. These pathways are intwined with emotional dysregulation and adoption of health-risk behaviours [24]. Studies on associations between ACEs and health typically use total ACE scores (the total number of adversities experienced by an individual) which has limitations including the assumption that constituent ACEs contribute equally to their impact on health [13]. Nonetheless, total ACE scores have been consistently associated with increased risk for poorer health with a gradient [15].

Compared to heterosexual individuals, SM groups report both a higher prevalence of ACEs overall, and specific types like sexual, verbal, and physical abuse and ACEs unique to non-heterosexual populations, such as heterosexist-based bullying and religious trauma [3, 25–29]. Studies indicate that ACEs mediate the relationship between SM identity and poor mental health, possibly implicating a greater burden of ACEs in SM individuals in explaining worse mental health in this population [30–32]. However, limited studies have investigated whether ACEs themselves differentially impact SM individuals, compared to heterosexual peers, in associations with adverse health. A recent study comprising Danish adults suggested SM individuals (especially bisexual), have worse mental health compared to their heterosexual peers with the same ACE burden [33]. However, whether associations between ACEs and mental health differ by sexual identity using interactions is largely unexplored. McCabe et al. found evidence for a slight differential impact of ACEs, by sexual orientation, on substance use/mental health disorders in an adult US population while Clements-Nolle et al. did not find any interaction but demonstrated a disproportionate increase in risk for suicidality in SM adolescents with a high ACE burden [34, 35]. This existing evidence is largely based on cross-sectional studies comprising adults and retrospectively collected ACE-data. However, longitudinal studies specifically investigating whether ACEs differentially increased risk for adverse health and health-risk behaviours are lacking, especially in adolescence.

We build on existing literature by exploring moderation by sexual identity in the associations of ACEs with a wide range of mental and general health outcomes, and health-risk behaviours in adolescents on the cusp of adulthood (i.e., late adolescence). We investigated outcomes relevant to adolescents using prospectively collected ACE data in a nationally representative UK birth cohort [36].

This study investigated whether 1. SM adolescents have higher risk for ACEs compared to their cisgender heterosexual peers. 2. ACEs in early- to mid-childhood (ages 3 to 14 years) are associated with higher risk for adverse health and health-risk behaviours in SM individuals compared to heterosexual peers in later adolescence (aged 17 years).

## Materials and methods

### Study design and participants

This longitudinal study used data from the Millennium Cohort Study (MCS), a nationally representative birth cohort following children born Sept 2000-Jan 2002 [37]. A national sample of 19,517 children from across the UK were recruited to the MCS and followed-up over seven sweeps (ages 9 months, 3, 5, 7, 11, 14 and 17 years). The MCS includes children living in non-household situations and children who were not born in the UK but lived in the country at recruitment. From age seven, cohort members completed their own questionnaire, as well as data being gathered in all waves from main carers (97% biological mothers).

The eligible sample for this study included 10,757 children (from 10,625 families or 73.6% of the eligible sample at this sweep) who attended the age 17 sweep. Further, the MCS used a stratified clustered framework and was oversampled to have higher proportions of ethnic minority (EM) participants and socioeconomically disadvantaged families (with appropriate sample weighting ensuring national representativeness). Attrition at the age 17 sweep was predicted by single-parent families, parents with lower-income occupation and lower educational level, Black ethnicity, and male sex. The final study sample included 8,686 children with data on all variables of interest.

Ethics approval for the MCS study was obtained from the National Research Ethics Service Committee London—Central (reference 13/LO/1786). All data are anonymized and available to researchers via the UK Data Service after registering online and via special license. Cohort members ≥16 years provided verbal consent to take part in the overall assessment, and each survey element.

### ACEs

Both parent- and child-reported ACEs were recorded between ages 3 and 14 in the MCS. We included eight ACEs (seven reported by the main carer–the mother in almost all cases, and one reported by the cohort member themselves). ACEs were operationalised based on previous studies using the MCS data and commonly accepted approaches in the evidence base [38, 39]. Six ACEs were categorised as binary variables based on answers on exposure (yes/no) and sometimes frequency of exposure. Parental mental health was assessed using the validated 6-item Kessler psychological distress scale which assesses symptoms of nonspecific psychological distress (e.g. “How often do you feel depressed?”) in the preceding four weeks. Items were scored to create an overall score which was categorised into a binary variable (scores ≥13 indicated nonspecific serious psychological distress or diagnosable mental illness) [40]. Harsh parenting was derived from summing the binarized responses to a series of six questions from Straus’ conflict tactics scales to create a summary score which was then dichotomised as a score ≥5 indicating ‘harsh parenting’ at that age. This operationalisation method has been used before in the MCS given the six items demonstrated ‘excellent construct validity’ [38, 41]. Complete details of all ACEs (including the original questions, ages when recorded and final form used in analysis are described in Table 1). Data from all ACE-related questions were coded to create binary variables (either not experiencing or experiencing the ACE). We estimated a cumulative adversity score variable (ACE scores) indicating the total number of ACEs experienced by a participant between ages 3–14 with the categories: 0 ACEs, 1 ACE, 2 ACEs and ≥3ACEs. To examine whether associations differed by types of ACEs, they were grouped into 1 Parental (5 ACEs), 2 Parenting (2 ACEs) and 3 Bullying (Table B in S1 Appendix).

**Table 1:** Information on the eight ACEs included in this study on 8,686 adolescents from the Millennium Cohort Study.

Information of operationalisation of Adverse Childhood Experiences (ACEs)		
ACE	Operationalisation	Ages of data collection
Parental divorce	The main carer was asked about marital status to identify occurrence of divorce or legal separation. ***Dichotomized*: *occurrence of divorce/legal separation vs*. *no experience of divorce/legal separation***	3, 5, 7, 11, 14
Parental psychological distress	Kessler score calculated from 6 components each scored 0–4, hence total Kessler score at each sweep derived as a score from 0–24. Binarized as ≥13 to represent psychological distress at a given age. ***Dichotomized*: *Kessler ≥ 13 at any given age vs*. *Kessler ≤12 at all sweeps***	3, 5, 7, 11, 14
Parental problem drinking	Based upon 3 variables. • Main carer CAGE questionnaire at Age 3 ○ *Dichotomized*: *CAGE = 2/3/4 indicating problem drinking vs CAGE = 0/1* • Age 11 parental alcohol use questions ○ “not being able to stop drinking”, “failed to do as expected due to drinking”, “relatives and friends have been concerned about drinking behaviour” ○ *Dichotomized “Yes” to all three questions indicating problem drinking vs*. *“No” to all three questions* • Age 14 parental alcohol use questions ○ “not being able to stop drinking”, “failed to do as expected due to drinking”, “relatives and friends have been concerned about drinking behaviour” ○ *Dichotomized “Yes” to all three questions indicating problem drinking vs*. *“No” to all three questions* ***Dichotomized*: *problem drinking as defined by any of the three variables above vs*. *none of the three variables above***	3, 11, 14
Parental recreational drug use	Main carer asked whether they have used recreational drugs in the last 12 months (occasionally, regularly, never) ***Dichotomized*: *occasionally/regularly at any age vs*. *never at all age***	3, 5, 14
Domestic violence	The main carer was asked about the use of force by the partner in relationship (Yes or No) ***Dichotomized*: *yes at any age vs*. *no at all ages***	3, 5, 7, 11, 14
Smacking (by parent)	The main carer was questioned about ‘How often smacks the child when naughty? (Daily, about once a week or more, once a month, rarely or never) ***Dichotomized*: *daily/weekly at any age vs*. *monthly/rarely/never at all ages***	3, 5, 7
Harsh parenting	Based on 6 questions from Straus’ conflict tactics scales posed to main carer at each of ages- 3, 5, 7 How often do you do the following when child is being naughty? (*Each dichotomized daily/weekly/monthly indicating yes vs*. *rarely/never indicating no*): • Ignore them • Shout at them • Send them to their room/naughty chair • Take away treats • Tell them off • Bribe them Summary score obtained by summing yes responses, creating sweeps-specific score (range 0–6) at age-3, 5, 7. (*Binarized as score ≥ 5 indicating harsh parenting at that age vs*. *score < 5*) ***Dichotomized*: *sweep-specific score ≥ 5 at any of ages 3*, *5*, *7 indicating harsh parenting vs*. *all sweep-specific scores < 5***	3, 5, 7
Bullying	Based on three variables • Cohort member asked ‘How often do other children hurt you/pick on you on purpose?’ at Age 11 ○ *Dichotomized*: *most days/weekly indicating bullying vs*. *monthly/every few months/less often/never* • Cohort member asked ‘How often do other children hurt you/pick on you on purpose?’ at Age 14 ○ *Dichotomized*: *most days/weekly indicating bullying vs*. *monthly/every few months/less often/never* • Cohort member asked ‘‘How often have other children sent you unwanted or nasty emails, texts, or messages, or posted something nasty about you on a website?” at Age 14 ○ *Dichotomized*: *most days/weekly indicating cyberbullying vs*. *monthly/every few months/less often/never* ***Dichotomized*: *any of the three variables above indicating experience of bullying at age 11/14 vs*. *none of the three variables above***	11, 14

### Sexual and ethnic identities

Participants were asked at age 17, ‘*Which of the following options best describes how you currently think of yourself’ and* could choose from six options. Based on responses, participants were categorized into 1. Completely heterosexual and 2. Gay/lesbian (mainly gay/lesbian or completely gay/lesbian) and 3. Bisexual. ‘Mainly heterosexual’ individuals and those who did not identify with any of the above categories were excluded from analysis (Table 2).

**Table 2:** Information on ethnicity, sexual identity, and childhood socioeconomic position of 8,686 adolescents aged 17 years from the Millennium Cohort Study.

Information of ethnicity of participants (Final N = 8,686)	
Ethnicity	Frequency (%)
White	6988 (80.5)
Mixed ethnicity	244 (2.8)
Indian	260 (3.0)
Pakistani	512 (5.9)
Bangladeshi	229 (2.6)
Black Caribbean	92 (1.1)
Black African	200 (2.3)
Other	161 (1.9)
**Information on sexual identity of participants (Final N = 8686)**	
**Sexual identity**	**Frequency (%)**
Heterosexual	7791 (89.7)
Bisexual	648 (7.5)
Gay/Lesbian (includes mainly gay/lesbian and completely gay/lesbian)	247 (2.8)
Mainly heterosexual (***excluded*** n = 1081) Other (***excluded*** n = 154)	
**Information on childhood socioeconomic position (SEP) (Final N = 8686)**	
**Household income quintile**	**Frequency (95% CI)**
1 (lowest income)	1782
2	1825
3	1754
4	1625
5 (highest income)	1700

Parent/guardian reported participant’s ethnicity at age 3 (original categories in Table 2). Information from subsequent ages was used to replace any missing ethnicity at age 3. For analysis, subjects were grouped into one of two ethnic groups, creating a binary variable: 1. White (ethnic majority) and 2. EM (mixed-ethnicity, South Asian, Black and ‘other’). The ‘other’ group included participants from Asia (excluding South Asia), the Middle East and South America. The mixed ethnic group included any combination of mixed-ethnic backgrounds.

### Mental and general health

Questionnaires assessed health indicators and health-risk behaviours answered by adolescents at age 17. Mental health indicators included continuous and binary variables of the Kessler Psychological Distress Scale for nonspecific psychological distress, and the self-reported Strengths and Difficulties Questionnaire (SDQ-S) which assesses behavioural markers of mental health difficulties in young people (including its 5 subscales assessing conduct problems, hyperactivity/inattention, emotional symptoms [depression/anxiety], peer problems and prosocial behaviour difficulty) [42]. The total scores for Kessler and each SDQ-S subscale were categorized into binary variables based on recommended cut-off points to indicate individuals with and without difficulties [40, 43]. Other mental health indicators included doctor diagnosed depression in the preceding year, self-harm (actions like burning, bruising/pinching, taking an overdose of tablets and pulling out hair*)*, attempted suicide and self-esteem (assessed using the 5-item Rosenberg self-esteem scale). Mental wellbeing was assessed using the shortened Warwick-Edinburgh Mental Wellbeing Scale which provides a single summary score indicating overall wellbeing. Internal consistency of the different scales was assessed using Cronbach’s alpha and was found to be acceptable (α>0.7).

General health indicators included self-assessed general health, chronic physical or mental health conditions in the preceding year, quality of sleep and body mass index (continuous BMI, and categorized into normal vs. obesity [including overweight] using the International Obesity Task Force age- and sex-specific cut-offs for 2-18-year-olds). Social adversity was assessed by experiences of victimization (i.e., experiences of verbal, physical, sexual assault and/or harassment in the past year).

The original questions and full details on the operationalisation of health and health-risk behaviour outcomes are described in Table A in S1 Appendix.

### Health-risk behaviours

These were coded as binary indicators (never tried/experienced/none vs. yes) and included smoking habits (non-smokers and regular smokers), frequent binge drinking in previous 4 weeks, recreational drug use, and specifically frequency of cannabis use in the previous year. Sexual behaviour was assessed by risky sex (did not use any contraception). We also examined frequency of physical activity in the previous week (none vs. any). Antisocial behaviour was assessed by one or more of the following acts in the previous 12 months: Pushed or shoved/hit/slapped/punched someone, hit someone with or used a weapon, stolen something from someone, harassed someone via mobile phone/email, sent pictures or spread rumours about someone and made unwelcome sexual approaches/sexually assaulted someone. The original questions, complete component items of each scale and all health-risk indicators, and how they were categorized (including references) are listed in Table A in S1 Appendix.

### Other covariates

Parental income was used as an indicator of socioeconomic position. Household income (Organisation for Economic Co-operation and Development UK) was categorized into equalized quintiles (where quintiles 1 and 5 represent the lowest and highest income quintiles respectively). Sex was based on sex at birth (hereafter sex).

### Statistical analysis

All analyses were conducted in Stata V17 (StataCorp LP, College Station, Texas).

Initial descriptive analysis included calculating total/grouped ACE scores, estimating the mean and prevalence/distribution of individual and grouped ACEs, for the entire study sample and by sexual identity. Differences in mean scores and prevalence of ACEs between sexual identity groups were estimated by unpaired t-tests and Wald tests run after univariate logistic regression, respectively.

First, we examined associations between sexual identity and risk for number of ACEs (total ACE scores) using multivariable multinomial logistic regression modelling (with adjustment for sex, ethnicity, and parental income). The multinomial logistic regression model calculates the relative risk ratio (RRR), which is the ratio of two relative risks (derived from the exponentiated multinomial logit coefficient) and is interpreted for a unit change in the predictor variable. Associations between sexual identity and risk for ACEs were also run with interactions between sex at birth and sexual identity to assess whether risk differed between males and females. The models were run as described above. Associations between the two forms of ACE indicators (total and grouped scores) and all health outcomes were analysed using multivariable linear and logistic regression modelling (for continuous and binary outcomes, respectively). All regression models included interaction terms between ACE scores and sexual identity variables to assess whether associations with outcomes differed by sexual identity. The predicted probabilities (margins) for each outcome for each of the six categories (generated by the interaction terms between the ACE and sexual identity categories) were estimated using the *‘Margins’* command in Stata. Predicted probabilities were also visualised to aid understanding of the interactions between ACE and sexual identity variables. To account for the stratified cluster design of the MCS and attrition over time, all regression analyses were weighted with non-response weights from the birth sweep (using the Stata ‘*svy*’ command for survey data). All models were adjusted for sex at birth, parental income and ethnicity.

### Missing data

Missing data in the five sweeps (ages 3 to 14) was addressed using multiple imputation with chained equations assuming data missing at random (MAR and including 25 imputations) [44]. The MAR mechanism (often largely untestable) implies that systematic differences between the missing values and the observed values can be explained by observed data, which is a plausible assumption in the British birth cohorts given the rich data available from birth. In addition to ethnicity and socioeconomic indicators, it is reasonable to assume ACEs are also MAR given their association with many variables including the wide range of health outcomes and health-risk behaviours in the dataset [20]. The main purpose of imputation was to address missing data in ACEs. For most ACEs, the proportions were very similar in complete case and imputed data (Table W in S1 Appendix). As long as a study participant had data on at least one ACE (recorded at any age), then the participant was included in the sample, and any missing ACE data was imputed. Ethnicity and parental income were available >98% of study participants. All participants had data on sex at birth. The imputation model also included BMI at each age and birth weight of study participants, and parental educational level as auxiliary variables to help strengthen the quality of imputed data. Auxiliary variables with stronger associations with incompletely observed variables or the probability of data being missing, increases the potential for reducing bias [45]. Further, including both baseline and longitudinal auxiliary variables increases the efficiency of imputing missing ACE data [45].

## Results

Table 3 and Table B in S1 Appendix display the distribution of individual, total and grouped ACE scores respectively, stratified by sexual identity. The most reported ACEs were maternal harsh parenting (55%) and physical punishment (26%) in the full sample. In general, there were no large differences in the distribution of individuals ACEs between heterosexual and SM individuals except for bullying (experienced by 39.1% of lesbian/gay individuals compared to 21.7% of heterosexual peers). The proportion of individuals reporting no ACEs was higher in heterosexual (19.5%) compared to SM (15.6%) groups. Further, 26.7% of SM individuals experienced ≥3ACEs compared to 21.2% of heterosexual peers.

**Table 3:** Distribution of Adverse Childhood Experiences (ACEs) in 8,686 adolescents aged 17 years from the Millennium Cohort Study by sexual identity [% (95% CI)].

		ALL N = 8,686	Heterosexual N = 7,791	Bisexual N = 648	Lesbian or Gay N = 247	Sexual minority (bisexual + lesbian/gay combined) N = 895
**Adverse Childhood Experience (ACE)** ^**1**^	Maternal drug use	7.0 [6.4, 7.6]	6.7 [6.1, 7.4]	**9.3 [7.0, 11.7]**	**9.7 [5.8, 13.6]**	**9.4 [7.4, 11.5]**
	Maternal psychological distress	14.1 [13.3, 14.9]	14.0 [13.2, 14.8]	14.3 [11.5, 17.2]	15.4 [10.6, 20.3]	14.6 [12.2, 17.1]
	Domestic violence against mother	13.3 [12.3, 14.3]	13.0 [11.9, 14.1]	16.2 [12.8, 19.6]	14.8 [9.4, 20.1]	**15.8 [12.9, 18.7]**
	Maternal physical punishment	25.5 [24.5, 26.6]	25.7 [24.6, 26.8]	23.0 [19.6, 26.5]	25.1 [19.3, 30.8]	23.6 [20.7, 26.5]
	Maternal harsh parenting	54.9 [53.7, 56.0]	55.0 [53.7, 56.2]	53.8 [49.7, 57.9]	54.6 [48.0, 61.1]	54.0 [50.5, 57.5]
	Parental separation/divorce	18.1 [17.2, 18.9]	17.9 [17.0, 18.8]	20.9 [17.6, 24.2]	16.1 [11.2, 21.0]	19.6 [16.8, 22.3]
	Maternal problematic drinking	7.8 [6.9, 8.7]	7.6 [6.7, 8.5]	9.4 [6.8, 12.0]	8.7 [4.4, 13.0]	9.2 [7.0, 11.5]
	Bullying	21.7 [20.8, 22.6]	20.3 [19.4, 21.3]	**31.9 [28.3, 35.6]**	**39.1 [32.9, 45.4]**	**33.9 [30.8, 37.1]**
**Breakdown of total ACE score by sexuality [% (95% CI)]**						
**Total ACE score** ^**2**^	0	19.1 [18.1, 20.1]	19.5 [18.4, 20.6]	16.2 [13.0, 19.4]	14.2 [9.5, 18.8]	15.6 [13.1, 18.2]
	1	32.9 [31.8, 34.0]	33.0 [31.8, 34.3]	32.2 [28.2, 36.2]	31.6 [25.1, 38.2]	32.0 [28.7, 35.3]
	2	26.3 [25.1, 27.4]	26.4 [25.2, 27.6]	24.6 [20.8, 28.5]	28.4 [21.6, 35.1]	25.7 [22.4, 28.9]
	≥3	21.7 [20.7, 22.7]	21.2 [20.1, 22.2]	27.0 [23.2, 30.8]	25.8 [19.8, 31.9]	26.7 [23.4, 30.0]
**Mean total ACE score by sexuality [95% CI]**						
**Mean total ACE score** ^**3**^		1.62 [1.59,1.65]	1.60 [1.57,1.63]	**1.79 [1.68,1.90]**	**1.83 [1.66,2.01]**	**1.80 [1.71,1.89]**

1. Estimates in **bold** indicate a statistically significant difference in proportions of this ACE in this sexual identity group compared to heterosexual individuals (p ≤ 0.05 for Wald test after univariate logistic regression)

2. Wald test performed on univariate multinomial logistic regression was used to confirm differences in the distribution of total ACE scores across sexual identity groups (p ≤0.05 indicated statistically significant differences)

3. Estimates in **bold** indicate a statistically significant difference in the mean of the total ACE score in this sexuality group compared to heterosexual individuals (p ≤0.05 for unpaired t-test)

### Sexual identity-related differences in having ACEs

Table 4 and Table C in S1 Appendix display results from models examining associations between sexual identity and total ACE scores and grouped ACEs respectively. SM individuals were more likely to have had ACEs compared to heterosexual peers. For example, both bisexual (RRR 1.87, 95%CI 1.35–2.57) and gay/lesbian (RRR 2.08, 1.24–3.48) individuals were twice as likely to have had ≥3ACEs compared to heterosexual peers. There were no differences between bisexual, gay/lesbian individuals and heterosexual peers in having parenting ACEs. However, bisexual individuals were more likely to have had parental ACEs (RRR 1.70, 1.26–2.29) compared to heterosexual peers. Gay/lesbian (RRR 2.11, 1.71–2.60) and bisexual (RRR 2.74, 1.97–3.80) individuals were more than twice as likely to experience bullying compared to heterosexual peers.

**Table 4:** Associations between sexual identity and risk for Adverse Childhood Experiences (ACEs) in 8,686 adolescents aged 17 years from the Millennium Cohort Study (estimates are from multivariable multinomial logistic regression with adjustment for sex at birth, ethnicity, and childhood socioeconomic position).

Total ACE = 0 (reference)		
Total ACE score = 1		
Variable	Category	Relative Risk Ratio [95% CI]
Sexual identity	*Heterosexual*	*Reference*
	Bisexual	1.22 [0.90,1.67]
	Gay/Lesbian	1.35 [0.80,2.29]
Sex	Female	*Reference*
	Male	**1.21 [1.04, 1.42]**
Ethnicity	White	*Reference*
	Ethnic minority	1.08 [0.85,1.38]
Childhood socioeconomic position (parental income quintile)	5 (highest income)	*Reference*
	4	1.14 [0.89,1.47]
	3	1.01 [0.83,1.23]
	2	1.13 [0.87,1.46]
	1 (lowest income)	0.99 [0.76,1.29]
**Total ACE score = 2**		
**Variable**	**Category**	**Relative Risk Ratio [95% CI]**
Sexual identity	*Heterosexual*	*Reference*
	Bisexual	1.29 [0.95,1.75]
	Gay/Lesbian	1.61 [0.97,2.67]
Sex	Female	*Reference*
	Male	**1.33 [1.12, 1.57]**
Ethnicity	White	*Reference*
	Ethnic minority	1.22 [0.95,1.58]
Childhood socioeconomic position (income quintile)	5 (highest income)	*Reference*
	4	**1.37 [1.05,1.78]**
	3	1.24 [0.98,1.57]
	2	**1.70 [1.30,2.22]**
	1 (lowest income)	**1.59 [1.21,2.09]**
**Total ACE score ≥3**		
**Variable**	**Category**	**Relative Risk Ratio [95% CI]**
Sexual identity	*Heterosexual*	*Reference*
	Bisexual	**1.87 [1.35,2.57]**
	Gay/Lesbian	**2.08 [1.24,3.48]**
Sex	Female	*Reference*
	Male	**1.65 [1.39, 1.96]**
Ethnicity	White	*Reference*
	Ethnic minority	1.12 [0.87,1.45]
Childhood socioeconomic position (income quintile)	5 (highest income)	*Reference*
	4	1.27 [0.94,1.71]
	3	**1.68 [1.28,2.20]**
	2	**2.98 [2.22,4.01]**
	1 (lowest income)	**3.20 [2.44,4.20]**

Estimates in **bold** indicate statistical significance i.e., 95% CI does not include 1.

### Sexual identity related differences in associations between (total) ACEs and health, and health-risk behaviours

**Fig 1:**
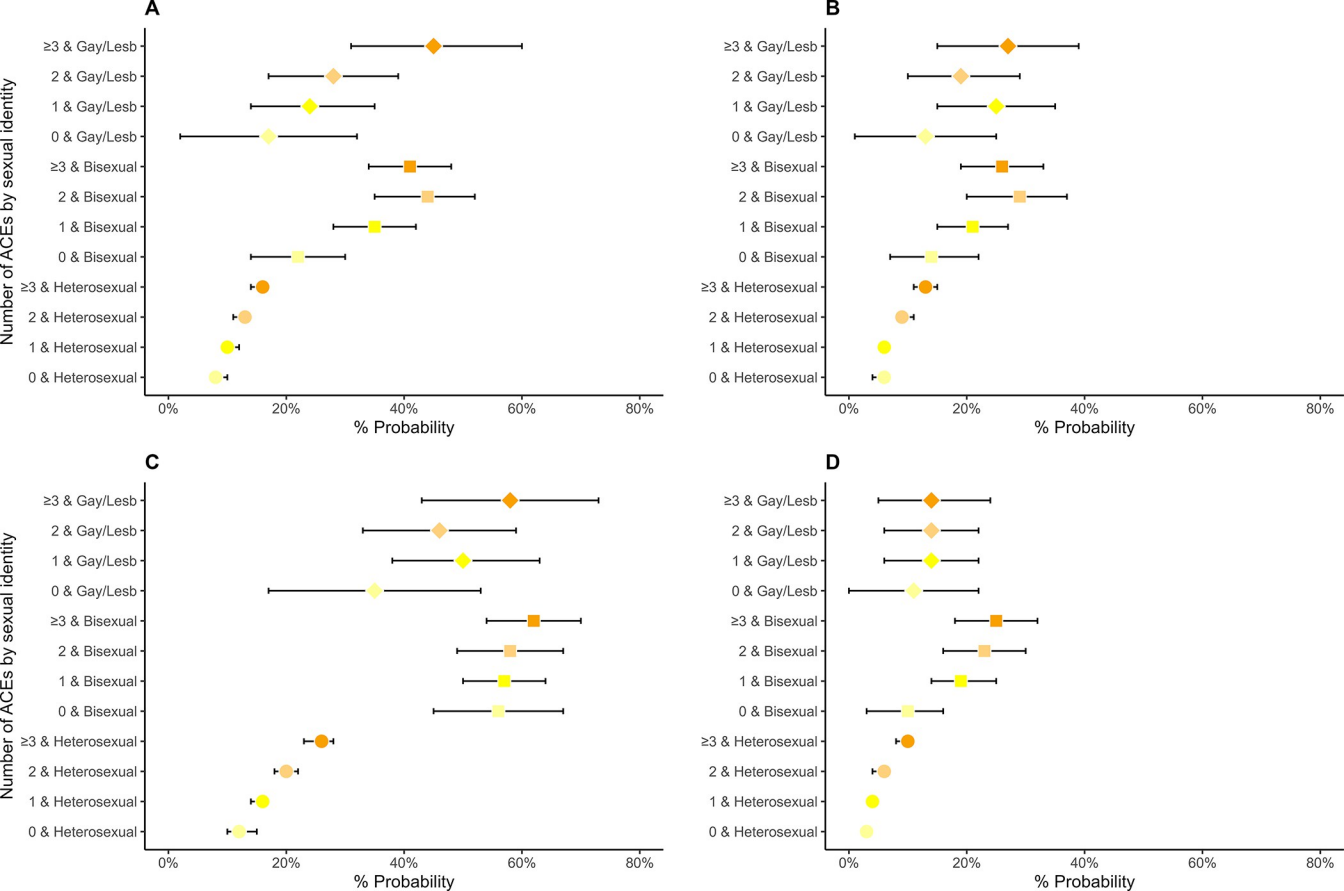
Associations between Adverse Childhood Experiences (ACEs) and mental health in 8,686 participants from the Millennium Cohort Study (estimates are predicted probabilities derived from logistic regression models adjusted for sex at birth, ethnicity, and childhood socioeconomic position). A = Psychological distress, B = Doctor-diagnosed depression or anxiety, C = Self harm, D = Suicidality.

**Fig 2:**
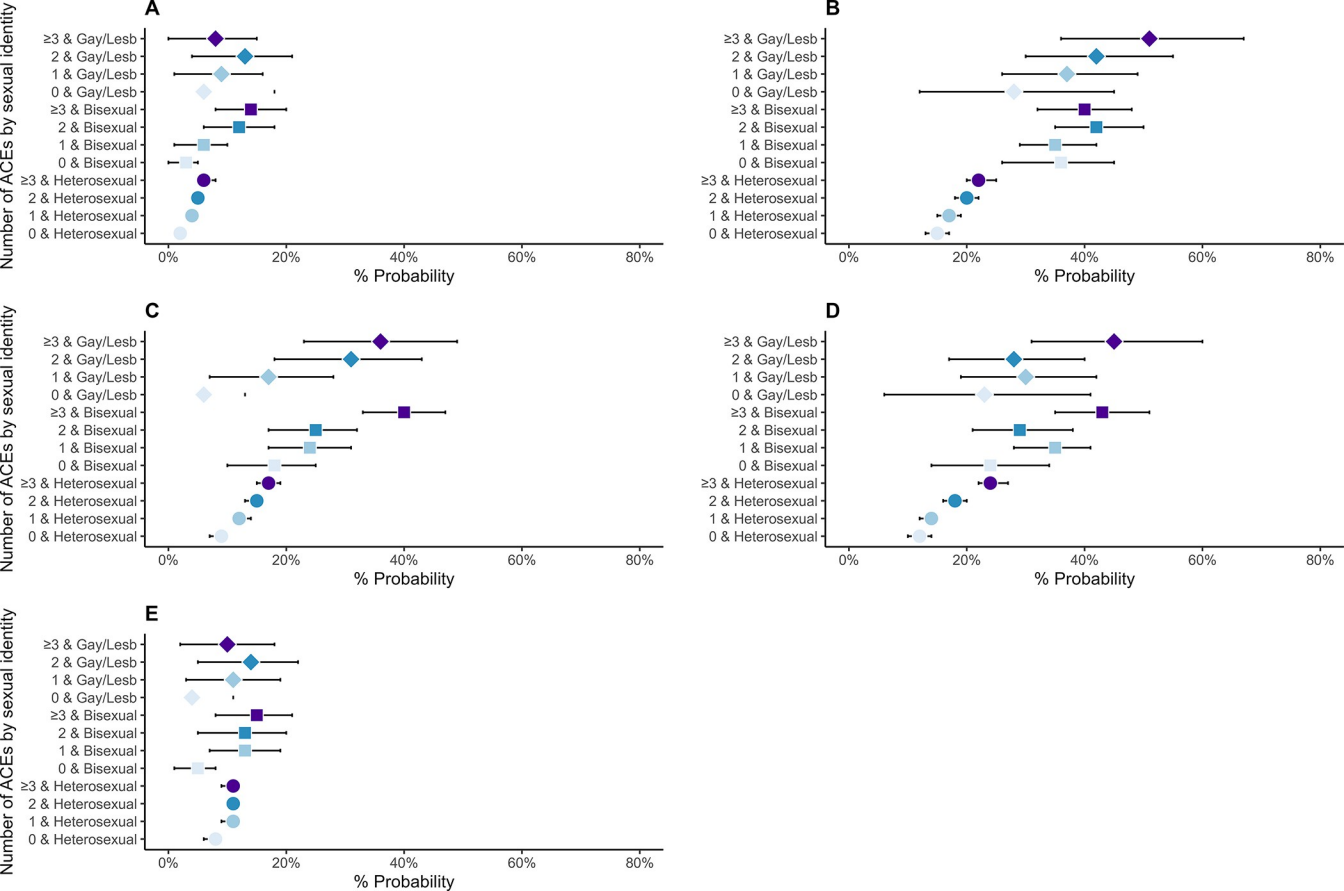
Associations between Adverse Childhood Experiences (ACEs) and mental health in 8,686 participants from the Millennium Cohort Study (estimates are predicted probabilities derived from logistic regression models adjusted for sex at birth, ethnicity, and childhood socioeconomic position). A = Conduct problems, B = Emotional symptoms, C = Hyperactivity/Inattention, D = Peer problems, E = Prosocial behaviour difficulty.

**Fig 3:**
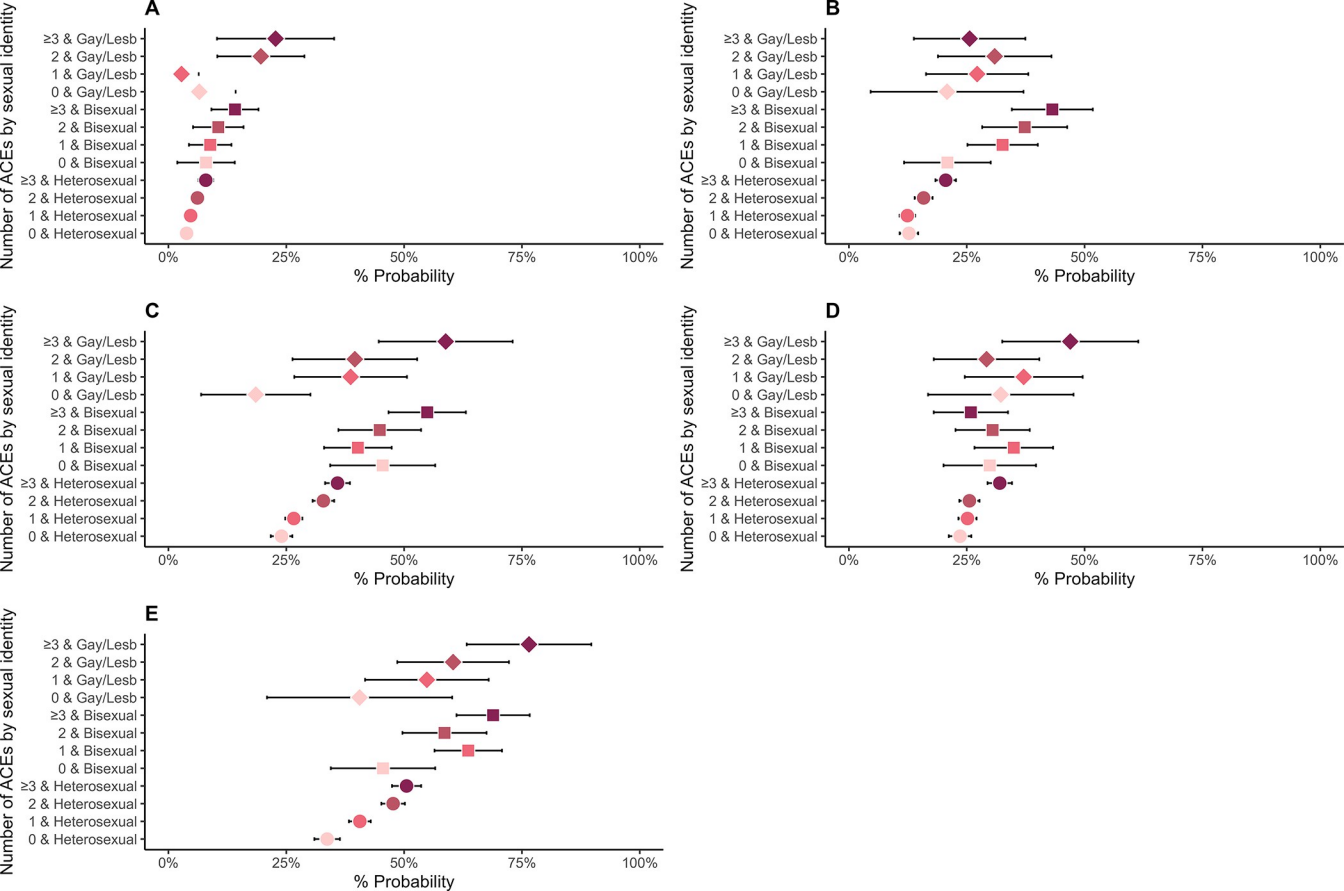
Associations between Adverse Childhood Experiences (ACEs) and general health in 8,686 participants from the Millennium Cohort Study (estimates are predicted probabilities derived from logistic regression models adjusted for sex at birth, ethnicity, and childhood socioeconomic position). A = Poor self-rated general health, B = Physical/mental health condition in past 12 months, C = Poor sleep quality, D = Overweight/Obese, E = Victimisation.

**Fig 4:**
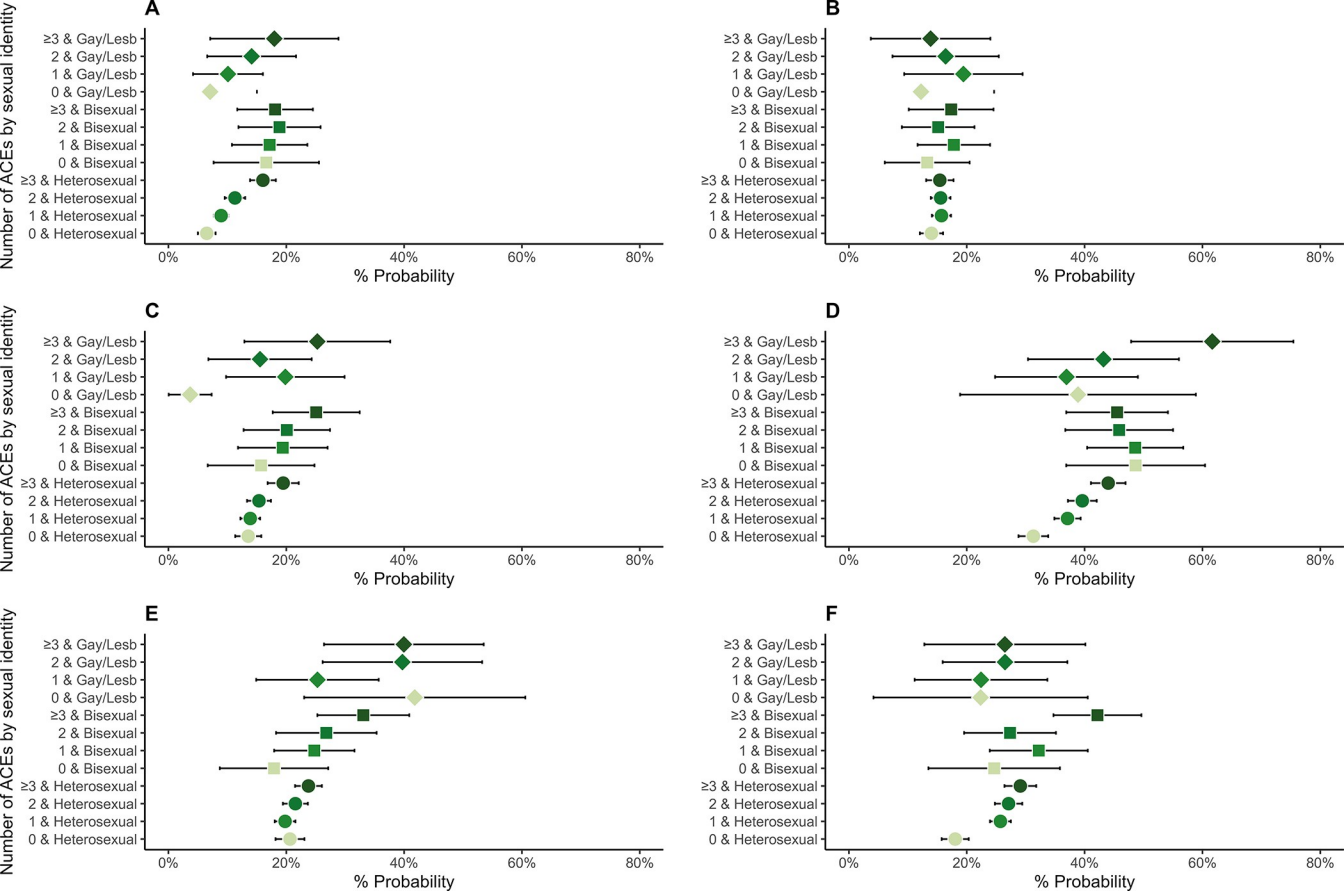
Associations between Adverse Childhood Experiences (ACEs) and health-risk behaviours in 8,686 participants from the Millennium Cohort Study (estimates are predicted probabilities derived from logistic regression models adjusted for sex at birth, ethnicity, and childhood socioeconomic position). A = Regular smoking habit, B = Frequent binge drinking, C = Frequent cannabis use, D = Sexual risk-taking, E = Lack of exercise, F = Antisocial behaviour.

Tables D and F in S1 Appendix (visualised in Figs 1–4) display results from logistic and linear regression models respectively (Tables E and G in S1 Appendix display the corresponding predictive margins or probabilities).

In general, compared to having no ACEs, individuals with ACEs had higher odds ratios (OR) for adverse health with an observable gradient (for e.g., OR 1.64 [95% CI 1.21,2.23] and 2.01 [1.51,2.68] for psychological distress in individuals with 2 and ≥3ACEs respectively, main effects in logistic regression models). Corresponding estimates for suicidality were OR 2.21 [1.38,3.54] and 3.86 [2.49,5.96], respectively. Individuals with ACEs were more likely to self-report adverse health-risk behaviours (for e.g., OR 1.84 [1.35,2.51] and 2.79 [2.08,3.75] for regular smoking for those with 2 and ≥3ACEs, respectively).

In general, we observed that the proportions of individuals who self-reported worse health increased in relation to both the number of ACEs and sexual identity (predicted probabilities, Figs 1–3, and Table E in S1 Appendix). For example, among individuals with no ACEs, 8% (95% CI: 7–10%) of heterosexual, 22% (14–30%) bisexual and 17% (2–32%) gay/lesbian adolescents reported psychological distress, which increased to 13% (11–14%), 44% (35–52%) and 28% (17–39%) for individuals with 2 ACEs and 16% (14–17%), 41% (34–48%) and 45% (31–60%) for individuals with ≥3ACEs, respectively. We observed a similar pattern with other indicators of mental health (SDQ emotional symptoms and peer problems subscales, self-harm, physical/mental health condition in previous year and poor sleep quality). Further, the higher number of individuals reporting poor mental health was particularly stark between 1) heterosexual individuals without ACEs and those with 2 or ≥3ACEs, 2) SM individuals and their heterosexual peers with 2 ACES or ≥3ACEs, though the pattern varied between gay/lesbian and bisexual individuals. For example, among heterosexual individuals, those reporting suicidality for 0, 1, 2 and ≥3ACEs were 3% (2–4%), 4% (3–5%), 6% (4–7) and 10% (8–11%), respectively. Corresponding numbers for bisexual individuals were 10% (3–16%), 19% (14–25%), 23% (16–30%), and 25% (18–32%) but there were minimal differences among gay/lesbian individuals with and without ACEs.

Greater numbers of individuals with ACEs reported certain adverse health-risk behaviours compared to those with no ACEs (Fig 4 and Table E in S1 Appendix). Further, higher numbers of SM individuals with ACEs reported adverse health-risk behaviours compared to heterosexual peers with the same number of ACEs. For example, 31% (29–34%) of heterosexual, 49% (37–60%) bisexual and 39% (19–59%) gay/lesbian individuals without ACEs reported risky sex, which increased to 44% (41–47%), 46% (37–54%) and 62% (48–75) in individuals with ≥3ACEs, respectively.

Tables U and V in S1 Appendix display results from models examining associations between sexual identity and total ACE scores which include interactions between sexual identity and sex. Male adolescents are at higher risk of accumulating ACEs (e.g. RRR 1.64, 1.38–1.96 for ≥3ACEs) compared to females. Higher proportions of heterosexual and SM males reported ACEs compared to female peers. For example, 22% of heterosexual males reported ≥3ACEs compared to 18% heterosexual females. 32% of bisexual and 31% of gay males reported ≥3ACEs compared to 24% of bisexual and lesbian females.

Results related to grouped ACE scores and all outcomes are presented in the Supplemental Results in S1 Appendix.

## Discussion

This study based on a nationally representative sample of adolescents found 1. Those identifying as SM were more likely to have experienced ACEs compared to cisgender heterosexual peers. 2. Individuals with ACEs were more likely to report worse mental health (consistent with two psychological distress assessment tools, doctor-diagnosed depression, and acute problems like self-harm and attempted suicide) compared to those without ACEs. Further, there was a gradient in the increase of risk of adverse health in relation to the number of ACEs. 3. Consistent patterns indicating that SM individuals with ACEs reported worse mental and general health, and some adverse health-risk behaviours compared to heterosexual peers with the same number of ACEs. 4. When examining different types of ACEs, parenting ACEs (like physical punishment) were not found to be associated with adverse health. But parental ACEs (like domestic violence, parental mental health) and bullying were consistently associated with adverse health and some health-risk behaviours.

Our study showed a higher average ACE exposure and higher risk for the accumulation of multiple ACEs in sexual-minority individuals compared to their heterosexual peers, which corroborates with literature on this topic [25–27]. Notably, we found SM individuals to be at an alarmingly higher risk of being bullied/cyberbullied, in line with existing evidence [4, 46].

Our findings of an increased risk of mental health and well-being problems in SM populations agree with previous reviews and meta-analyses [3, 47]. In our sample, SM individuals (especially bisexual) had higher risk for psychological distress, doctor diagnosed depression/anxiety, self-harm, and suicidality. Our study also addresses several improvements called for by Jonas et al. in the existing literature, namely prospective ACE collection and longitudinal study design [3].

Previous studies have examined the effect of sexual identity on the relationship between ACEs and mental health and health-risk behaviours in a variety of ways. This has included testing for loss of estimate significance on introducing sexual identity as an additional variable in a model between ACEs and MH/HRB outcomes, stratification by sexual identity, and including interaction terms between sexual identity and ACEs [32–35]. We chose the latter technique and report results similar to the existing literature, including a higher risk for a variety of adverse mental health outcomes and certain health-risk behaviours in SM individuals with ACE exposure relative to heterosexual individuals [32–34]. Further, as with Clements-Nolle et al. we found substantial increases in risk for suicidality in SM individuals (specifically bisexual individuals in our sample) with exposure to ACEs compared to their heterosexual peers with analogous ACE burden [35]. A unique contribution of our study (and not previously reported) was to separate the eight ACEs into three types: parental, parenting, and bullying (which also corresponds to parent-reported and self-reported ACEs). As indicated by our findings, it is parental ACEs (like poor mental health or alcohol misuse) and bullying which were found to be detrimental for health in adolescents and sexual minority individuals.

The main strengths of this study include a large sample size drawn from a contemporary and nationally representative birth cohort, and self-reported sexual identities, adverse health and health-risk behaviours. A significant strength is the longitudinal study design which includes prospectively collected data on common ACEs, which is less likely than retrospective data to be affected by recall bias (and subsequent information bias as studies indicate that SM individuals are more likely to remember adverse experiences compared to heterosexual peers) or participants’ mental state [48–51]. However, reporting bias might still exist for some of the parent-reported ACEs as they might be less inclined to reveal certain sensitive information like drug and alcohol misuse, potentially resulting in misclassification and underestimation of some ACEs.

This study benefits from a wide range of health outcomes, especially multiple and diverse health and wellbeing indicators, and health-risk behaviours examined in the same sample. The consistent associations between ACEs and multiple mental health indicators among both heterosexual and SM groups reduces risk of chance findings. Despite significant attrition over follow-up (as common with most longitudinal studies of this kind), the MCS remains nationally representative at age 17.

At age 17, missing data on most outcomes and all confounders was relatively low (<10% for most outcomes and only three outcome variables had missing data ranging from 35% to 37%), but higher numbers of participants were missing data on ACEs (ranging from 15% to 57%). Nonetheless, missing data was addressed using the well-established and robust multiple imputation technique. While imputation relies on the MAR assumption which is not empirically verifiable, we increased the plausibility of the MAR assumption by including a varied set of auxiliary variables in the imputation model such as parental education level and birth weight [52, 53]. These variables contribute information which helps predict missing data with greater precision and minimises non-random variation in the values [54]. This is further strengthened by the longitudinal data of some variables and rich data available from birth, improving reliability of the imputed data.

895 (10.3%) individuals identified as being exclusively SM which is actually substantially higher than national estimates (3.2% in the 2021 UK census). However, with respect to modelling, the relatively small proportion of SM individuals results in loss of statistical power affecting the precision of some observed estimates (for e.g., the wide and/or overlapping confidence intervals related to interaction coefficients and predicted probabilities). This is in contrast to the narrower confidence intervals observed for heterosexual individuals compared to SM peers. However, the lack of statistical significance related to the interaction estimates does not necessarily imply that effect modification by sexual identity does not exist. In fact, we observed that individuals with ACEs consistently reported worse mental health and certain health-risk behaviours compared to peers without ACEs and these numbers were larger in relation to both SM-identity and the number of ACEs. Despite the relatively large sample size of 8700 individuals, we did not have the necessary power to examine associations between individual ACEs and each outcome or test for potential differences by sex. These are important limitations that should be explored in future studies.

We used two measurements of ACEs; total/cumulative ACE score and grouped ACEs, with both operationalisations having their strengths and weaknesses. Using total ACE scores (the most widely adopted methodology to study ACEs) assumes that each constituent ACE is equally important for outcomes, which is unlikely although this approach facilitates comparisons with similar studies as total ACE scores are commonly used [55]. Further, total ACE scores acknowledge the high probability of co-occurring ACEs and are relatively simple tools to implement in clinical and policy settings to help identify individuals with higher risk for adverse outcomes [13]. We grouped ACEs into parental vs. parenting types for two main reasons: 1) this gives some indication of those ACEs that are potentially more detrimental to health in later adolescence 2) provides an indication of which ACEs can be prioritised for targeted public health intervention. We examined bullying as a separate ACE on its own as the short- and long-term impact of bullying on health, especially in adolescence, is well established, and further, SM adolescents are more likely to be bullied compared to heterosexual peers [56]. We acknowledge that going beyond the ACE score is essential to inform public policy and practice so must be studied where data allows.

There were also limitations related to the collection of ACE data in the MCS. We were unable to include relevant ACEs like sexual abuse, sexual- and ethnic-identity related abuse, and discrimination which are higher in prevalence in minoritised individuals. Adversities experienced by parents outside the family such as bullying were also uncaptured. Further, since data collection on all ACEs was far from homogenous through the sweeps, we were unable to assess the temporal aspect of ACE effects on subsequent health. Hence, deeper investigation into the temporality and potential weighting of ACEs is required as research progresses. It is also possible that individuals with more severe ACEs are more likely to be lost to follow-up which can lead to an underestimation of individual ACEs and the ACE scores. Adolescents with dual ethnic and sexual minority identities can be differentially impacted by ACEs compared to White SM peers and must be examined in future studies. Finally, our study examined associations, and the results cannot derive any causal inferences.

We excluded individuals who identified as ‘mainly heterosexual’ due to ongoing discussion on whether these individuals should be in a separate sexual identity category or combined with bisexual individuals. In fact, a recent study using the same data as ours combined mainly heterosexual and bisexual individuals in the same group, an approach we do not recommend [57]. The term ‘mainly heterosexual’ is not commonly used in surveys and questionnaires in the UK and there is strong likelihood for it to be confused with ‘bisexual’. Further, individuals unsure of their sexual identity (more likely in adolescence) may be unsure of which category to choose. We thus decided for the purpose of this study to include those who identified as completely heterosexual, bisexual, or gay/lesbian as they are more likely to be sure about their sexual identity. Nonetheless, adolescents identifying as mainly heterosexual have increased risk for poorer mental and general health compared to heterosexual peers and we need to further examine health in this group while also understanding the similarities and differences with bisexual individuals [1].

Our findings clearly demonstrate that ACEs have a deleterious impact on health and health-risk behaviours in late adolescence. The associations between ACEs and adverse health are substantially strong in size/effect and have been shown in multiple populations across countries, indicating causal associations. SM individuals are likely differently impacted by both their higher prevalence of ACEs and the additional stigma, discrimination and SM-identity specific issues they face (like disclosure of sexual identity, internalised homophobia, finding supportive networks). Further, many of these issues are potentially heightened in adolescence, a period when individuals are exploring their sexual and other identities, while simultaneously also dealing with entering adulthood and making important decisions like pursuing higher education. Nonetheless, our findings indicate that primary prevention or minimising some ACEs is crucial. While it is not realistic to prevent all ACEs from occurring, the deleterious impact of ACEs needs to be highlighted in wider society by raising awareness, which may reduce the prevalence of some ACEs. However, certain ACEs can be targeted with prevention as a goal such as bullying in schools and elsewhere, alongside racism and discrimination. Our findings clearly indicate that SM individuals are more likely to experience bullying compared to heterosexual peers, and the deleterious impact of bullying on health is well established. Further, effective policies need to be developed to 1) Reduce the impact of some ACEs such as providing timely intervention and care for poor mental health to lessen the consequences of living with family members with mental health problems and engaging in health-risk behaviours, 2) Increase awareness among healthcare service providers, community leaders and school teachers about both the adverse consequences of ACEs on individuals in general and the additional added impact of ACEs on health in SM individuals in particular. Further, policies on treatment can be tailored according to the number and type of ACEs for different demographic groups as our findings suggest both increased risk for adverse health in relation to the number of ACEs and the type of ACEs. The latter is currently not part of any national health policy in the UK. A recent report on a qualitative study on lived experiences and mental health consequences in SM minority young people in the UK highlighted the severe lack of mental health support in general and specifically for SM individuals [58].

There is substantial scope for future research in this area. Relatively little is known about how ACEs impact health in ethnic minority youth and individuals living with multiple minority identities (for example, sexual-, ethnic- and religious-minority identities). Further, studies have not been able to comprehensively examine the interplay between ACEs, SM identity-related issues and health across all SM identities as such data requires substantial statistical power and longitudinal data which is largely lacking.

## Conclusions

In summary, our findings confirm previous research showing ACEs increase risk for adverse mental health and health-risk behaviours. We also confirm prior but limited research on the deleterious impact of ACEs on adverse health being more profound in SM individuals compared to heterosexual peers. This study is the first to examine the potential effect modification by sexual identity in the associations of ACEs and health in late adolescence using a nationally representative sample. Further, our findings are consistent with a wide range of health indicators. While there is considerable scope for targeted public health interventions to reduce the impact of ACEs on health in adolescents, our findings need to be further examined in larger studies.

## Supporting information

S1 Appendix Supplementary results and Tables A to W are in S1 Appendix. (DOCX)
