# Supplementary material for: Sexual identity-related inequalities in associations between adverse childhood experiences and health in late adolescence–A national cohort study
Source: PLoS One. 2024 Dec 11;19(12):e0312161. doi: 10.1371/journal.pone.0312161 (PMC11633990; doi:10.1371/journal.pone.0312161)
Supplement: S1 Appendix — (DOCX) [file pone.0312161.s001.docx]

**Supplementary file: Sexual identity-related inequalities in associations between adverse childhood experiences and health in late adolescence – A national cohort study**

Rahul Chandrasekar^1^, Alexis Karamanos^2^, Annastazia Learoyd^2^ and Amal R. Khanolkar^2*^

1. University College London Medical School, London, UK

2. Department of Population Health Sciences, King’s College London, London, UK

***** Correspondence: [amal.khanolkar@kcl.ac.uk](mailto:amal.khanolkar@kcl.ac.uk)

**Table of contents**

Supplementary Results …..……………………………….…………………………………….…………………………………….…………………………………….…………………………………….…………………………………….…………………………………….…………………………………….……………………………………………….3

[Table A - Information on grouped ACE scores used in this analysis 4](#_Toc137225639)

[Table B - Information on mental health, general health, and Health-Risk behaviour outcomes used in this analysis. 5](#_Toc137225640)

[Table C - Associations between sexual identity and types of Adverse Childhood Experience (ACE) exposure in 8,686 adolescents aged 17 years from the Millennium Cohort Study (estimates are from multivariable multinomial logistic regression with adjustment for sex at birth, ethnicity, and childhood socioeconomic status) 7](#_Toc137225641)

[Table D - Associations between Adverse Childhood Experiences (ACEs) and Mental Health, General Health, and Health-Risk Behaviours in 8686 adolescents from the Millennium Cohort Study (estimates are from logistic regression analysis adjusted for sex, ethnicity, and childhood socioeconomic status) 8](#_Toc137225642)

[Table E - Associations between Adverse Childhood Experiences (ACEs) and Mental Health, General Health, and Health-Risk Behaviour outcomes in 8686 adolescents from the Millennium Cohort Study (estimates are predictive margins of interactions between ACEs and Sexuality from logistic regression analysis adjusted for sex, ethnicity, and childhood socioeconomic status) 10](#_Toc137225643)

[Table F - Associations between Adverse Childhood Experiences (ACEs) and Mental Health in 8686 adolescents from the Millennium Cohort Study (estimates are from linear regression analysis adjusted for sex, ethnicity, and childhood socioeconomic status) 12](#_Toc137225644)

[Table G - Associations between Adverse Childhood Experiences (ACEs) and Mental Health outcomes in 8686 adolescents from the Millennium Cohort Study (estimates are predictive margins of interactions between ACEs and Sexuality from linear regression analysis adjusted for sex, ethnicity, and childhood socioeconomic status) 13](#_Toc137225645)

[Table H - Associations between Parental Adverse Childhood Experiences (ACEs) and Mental Health, General Health, and Health-Risk Behaviours in 8686 adolescents from the Millennium Cohort Study (estimates are from logistic regression analysis adjusted for sex, ethnicity, and childhood socioeconomic status) 14](#_Toc137225646)

[Table I - Associations between Parental Adverse Childhood Experiences (ACEs) and Mental Health, General Health, and Health-Risk Behaviour outcomes in 8686 adolescents from the Millennium Cohort Study (estimates are predictive margins of interactions between Parenting ACEs and Sexuality from logistic regression analysis adjusted for sex, ethnicity, and childhood socioeconomic status) 16](#_Toc137225647)

[Table J – Associations between Parental Adverse Childhood Experiences (ACEs) and Mental Health in 8686 adolescents from the Millennium Cohort Study (estimates are from linear regression analysis adjusted for sex, ethnicity, and childhood socioeconomic status) 18](#_Toc137225648)

[Table K - Associations between Parental Adverse Childhood Experiences (ACEs) and Mental Health outcomes in 8686 adolescents from the Millennium Cohort Study (estimates are predictive margins of interactions between Parental ACEs and Sexuality from linear regression analysis adjusted for sex, ethnicity, and childhood socioeconomic status) 19](#_Toc137225649)

[Table L - Associations between Parenting Adverse Childhood Experiences (ACEs) and Mental Health, General Health, and Health-Risk Behaviours in 8686 participants from the Millennium Cohort Study (estimates are from logistic regression analysis adjusted for sex, ethnicity, and childhood socioeconomic status) 20](#_Toc137225650)

[Table M - Associations between Parenting Adverse Childhood Experiences (ACEs) and Mental Health, General Health, and Health-Risk Behaviour outcomes in 8686 participants from the Millennium Cohort Study (estimates are predictive margins of interactions between Parenting ACEs and Sexuality from logistic regression analysis adjusted for sex, ethnicity, and childhood socioeconomic status) 22](#_Toc137225651)

[Table N - Associations between Parenting Adverse Childhood Experiences (ACEs) and Mental Health in 8686 participants from the Millennium Cohort Study (estimates are from linear regression analysis adjusted for sex, ethnicity, and childhood socioeconomic status) 24](#_Toc137225652)

[Table O -Associations between Parenting Adverse Childhood Experiences (ACEs) and Mental Health outcomes in 8686 participants from the Millennium Cohort Study (estimates are predictive margins of interactions between Parenting ACEs and Sexuality from linear regression analysis adjusted for sex, ethnicity, and childhood socioeconomic status) 25](#_Toc137225653)

[Table P - Associations between Experience of bullying (ACEs) and Mental Health, General Health, and Health-Risk Behaviours in 8686 participants from the Millennium Cohort Study (estimates are from logistic regression analysis adjusted for sex, ethnicity, and childhood socioeconomic status) 26](#_Toc137225654)

[Table Q - Associations between Experience of bullying and Mental Health, General Health, and Health-Risk Behaviour outcomes in 8686 adolescents from the Millennium Cohort Study (estimates are predictive margins of interactions between Experience of bullying and Sexuality from logistic regression analysis adjusted for sex, ethnicity, and childhood socioeconomic status) 28](#_Toc137225655)

[Table R - Associations between Experience of bullying in childhood and Mental Health in 8686 adolescents from the Millennium Cohort Study (estimates are from linear regression analysis adjusted for sex, ethnicity, and childhood socioeconomic status) 30](#_Toc137225656)

[Table S - Associations between Experience of bullying and Mental Health outcomes in 8686 adolescents from the Millennium Cohort Study (estimates are predictive margins of interactions between Experience of bullying and Sexuality from linear regression analysis adjusted for sex, ethnicity, and childhood socioeconomic status) 31](#_Toc137225657)

Table T – Information on prevalence of mental health, general health, and health-risk behaviour outcomes in 8,686 adolescents from the Millennium Cohort Study…………………………………………………………………………………………………………..32

Table U – Associations between sexual identity and risk for Adverse Childhood Experiences (ACEs) in 8,686 adolescents aged 17 years from the Millennium Cohort Study (estimates are from multivariable multinomial logistic regression with adjustment for ethnicity and childhood socioeconomic position)………………………………………………………………………………………………………………………………………………………………………………………………………………………………………………………………………………………..33

Table V – Associations between Sexual identity and risk for Adverse Childhood Experiences (ACEs) in 8,686 adolescents aged 17 years from the Millennium Cohort Study (estimates are predictive margins of interactions between Sexual identity and Sex from multivariable multinomial logistic regression with adjustment for ethnicity and childhood socioeconomic position)……………………………………………………………………………………………………………………………………………………………………………….34

Table W - Comparison of the distribution of Adverse Childhood Experiences (ACEs) in 8,686 adolescents aged 17 years from the Millennium Cohort Study from pre- and post-imputation data…………………………………………………………………35

**Supplementary results**

*Sexual identity related differences in associations between grouped ACEs and health, and Health-Risk behaviours*

*Parenting Adverse Childhood Experiences (Tables H, I, J and K in S1 Appendix)*

Parenting ACEs (maternal smacking and harsh parenting) were not found to be associated with adverse health and Health-Risk behaviours in their children. There was some indication that more bisexual adolescents with these ACEs reported hyperactivity (45% vs 35% for those with and without these ACEs respectively) and victimisation (64% vs 57%). However, there were no observable differences in other sexual-identity groups.

*Parental Adverse Childhood Experiences (Tables L, M, N and O in S1 Appendix)*

Parental ACEs include maternal psychological distress, recreational drug use, problematic drinking, domestic violence, and parental separation. Greater numbers of adolescents with parental ACEs reported worse mental health compared to their peers without ACEs with findings stronger in heterosexual group (for example, 9% [9-10] of individuals with 0 ACEs had parents with psychological distress, which increased to 14% [13-16] and 16% [13-18] among those with 1 and ≥2 ACEs respectively). Similarly, 15% [95% CI 14-17], 20% [18-22] and 25% [22-29] of heterosexual individuals reported self-harm among those with 0, 1 and ≥2ACEs, respectively). Differences between the numbers of SM adolescents with and without parental ACEs reporting adverse mental health was more limited but observed for psychological distress and poor quality of sleep.

*Bullying (Tables P, Q, R and S in S1 Appendix )*

In general, and irrespective of sexual identity, adolescents who experienced bullying self-reported worse mental health compared to those with no bullying expereinces. However, among those who experienced bullying, higher proportions of SM individuals reported worse mental health compared to heterosexual peers, with findings consistent with multiple indicators of mental health (SDQ emotional symptoms, hyperactivity and peer problems subscales, doctor diagnosed depression, suicidality, self-harm and poor quality of sleep). For example, among adolescents with no ACEs, 10% of heterosexual, 33% bisexual and 18% gay/lesbian individuals reported psychological distress, which increased to 17%, 44% and 47% respectively, among those who experienced bullying. Similarly, almost twice the number of heterosexual (28%, 25-30) and gay/lesbian (65%, 54-76) adolescents who experienced bullying reported self-harm compared to peers who did not experience bullying (15%, 14-17 in heterosexual and 38%, 29-47 in gay/lesbian individuals). Further, those who experienced bullying were also more likely to report adverse health-risk behaviours with differences more pertinent among heterosexual individuals. For example, among adolescents who did not experience bullying, 57% (56-59) of heterosexual and 65% (60-70) gay/lesbians individuals reported attempting to change weight which increased to 65% (63-68) and 78% (68-88) respectively, among those who did experience bullying.

Table A - Information on mental health, general health, and Health-Risk behaviour outcomes used in this analysis.

| **Outcome** | **Question(s) in cohort member computer-assisted personal interview (CAPI), self-completion interview (CASI) or online questionnaire (CAWI)** | **Binary or continuous** | **Comments** |
| --- | --- | --- | --- |
| **Mental health, wellbeing, and general health** | | | |
| General health description | How would you describe your health generally? Would you say it is: | Excellent/very good/good vs fair/poor |  |
| Warwick-Edinburg wellbeing scale (short version) | I’ve been feeling:  -optimistic about the future  -feeling useful  -feeling relaxed  -dealing with problems well  -thinking clearly  -feeling close to other people  -able to make up my own mind about things | Continuous | Each item coded 0-4 add total summed (/28)  4. All of the time  3. Often  2. Some of the time  1. Rarely  0. None of the time |
| Self-reported Kessler (6 item) | During the last 30 days about how often:  - did you feel so depressed that nothing could cheer you up?  - did you feel hopeless?  - did you feel restless or fidgety?  - did you feel that everything was an effort?  - did you feel worthless?  - did you feel nervous? | Binary (0-12 vs. 13-24) and Continuous | Each item coded 0-4 add total summed (/24)  4. All of the time  3. Most of the time  2. Some of the time  1. A little of the time  0. None of the time |
| Self-reported Strengths and Difficulties Questionnaire (SDQ)  -emotional subscale | Complains of headaches/stomach aches/sickness  Often seems worried  Often unhappy  Nervous or clingy in new situations  Many fears, easily scared. | Binary (0-5 vs. 6-10) and Continuous | Each item coded 0-2 and total summed (/10)  0. Not true  1. Somewhat true  2. Certainly true. |
| -conduct subscale | Often has temper tantrums  Generally obedient  Fights with or bullies other children  Steals from home, school or elsewhere  Often lies or cheats | Binary (0-4 vs. 5-10) and Continuous | Each item coded 0-2 and total summed (/10)  0. Not true  1. Somewhat true  2. Certainly true. |
| -hyperactivity subscale | Restless, overactive, cannot stay still for long  Constantly fidgeting  Easily distracted  Can stop and think before acting  Sees tasks through to the end | Binary (0-6 vs. 7-10) and Continuous | Each item coded 0-2 and total summed (/10)  0. Not true  1. Somewhat true  2. Certainly true. |
| -peer problems | I am usually on my own. I generally play alone or keep to myself  I have one good friend or more  Other people my age generally like me  Other children or young people pick on me or bully me  I get on better with adults than with people my own age | Binary (0-3 vs. 4-10) and Continuous | Each item coded 0-2 and total summed (/10)  0. Not true  1. Somewhat true  2. Certainly true. |
| -prosocial behaviour difficulty | I try to be nice to other people. I care about their feelings  Shares readily with others  Helpful if someone is hurt, upset or ill  Kind to younger children  Often volunteers to help others | Binary (6-10 vs. 0-5) and Continuous | Each item coded 0-2 and total summed (/10)  0. Not true  1. Somewhat true  2. Certainly true. |
| Rosenberg self-esteem scale  (5 item) | How much do you agree or disagree with the following statements about you?  On the whole, I am satisfied with myself  I feel I have a number of good qualities  I am able to do things as well as most other people  I am a person of value  I feel good about myself | Continuous | Each item coded 0-3 and total summed (/15)  0. Strongly disagree  1. Disagree  2. Agree  3. Strongly agree |
| Suicidality | Have you ever hurt yourself on purpose in an attempt to end your life? | No vs yes |  |
| Doctor diagnosed depression | Has a doctor ever told you that you suffer from depression or serious anxiety? | No vs yes |  |
| Mental or physical health condition in previous year | Do you have any physical or mental health conditions or illnesses lasting or expected to last 12 months or more? | No vs yes |  |
| Victimisation | In the past 12 months has anyone done any of these things to you?  Insulted/physical/hit/harassed/assaulted you. | No vs yes (any kind of victimisation) |  |
| Self-harm | During the last year, have you hurt yourself on purpose in any of the following ways?  Cut or stabbed yourself  Burned yourself  Bruised or pinched yourself  Taken an overdose of tablets  Pulled out your hair  Hurt yourself some other way | No vs yes (any kind of self-harm) |  |
| Sleep quality | During the past month, how would you rate your sleep quality overall? Would you say it has been…  1 …Very good  2 …Fairly good  3 …Fairly bad, or  4 …Very bad? | Very good/fairly good vs fairly bad/very bad |  |
| Weight perception (body image) | Which of these do you think you are?  1 Underweight  2 About the right weight  3 Slightly overweight  4 Very overweight | Right weight vs overweight/underweight | Which of these do you think you are? |
| Body Mass Index (kg/m^2^) |  | Continuous |  |
| Overweight/obesity |  | Normal vs. overweight/obesity |  |
| **Health and risky behaviours** | | | |
| Regular smoking habit | ** Participants asked to categorise themselves by frequency of cigarette smoking | Less than 1 per week vs. More than 1 per week |  |
| Frequent binge drinking | How many times have you had 5 or more alcoholic drinks at a time in the last year? | <10 times vs. ≥10 times |  |
| Recreational drug use (any drug use ever) | Have you ever taken any of the following?  Options included: Cannabis (Marijuana, Dope, Pot, Hash, Grass, Ganja, Weed)  Cocaine powder (Coke)  Acid or LSD  Ecstasy  Heroin  Crack  Speed or Amphetamines  Methamphetamine (crystal meth)  Ketamine  Mephedrone  Psychoactive substances | Never vs yes |  |
| Frequent cannabis use | In the past year how many times have you taken cannabis? | <10 times vs. ≥10 times |  |
| Risky sex (sex without use of contraception) | Do you or any partner regularly use any of these forms of contraception or protection when having sex together? | No vs yes |  |
| Lack of exercise in previous week | On how many days in the last week did you do a total of at least an hour of moderate to vigorous physical activity?  By moderate to vigorous we mean any physical activity that makes you get warmer, breathe harder and makes your heartbeat faster, e.g., riding a bike, running, playing football, swimming, dancing, etc. | Any amount vs. none |  |
| Attempting to change weight via exercise in past year | In the last 12 months, have you exercised to lose weight or to avoid gaining weight | No vs yes |  |
| Antisocial behaviour | Any antisocial behaviour in previous 12 months:  Pushed or shoved/hit/slapped/punched someone?  Hit someone with or used a weapon?  Stolen something from someone. e.g. a mobile phone, money etc.?  Harassed or bothered someone via mobile phone or email?  Sent pictures or spread rumours about someone via phone, email, social media or online?  Made an unwelcome sexual approach or assaulted someone sexually? | No vs. yes |  |

Table B - Information on grouped ACE scores used in the analysis including 8,686 adolescents from the Millennium Cohort Study

|  | | **ALL**  **N = 8,686** | **Heterosexual**  **N = 7,791** | **Bisexual**  **N = 648** | | **Lesbian or Gay**  **N = 247** | **Sexual minority**  **(bisexual + lesbian/gay combined)**  **N = 895** |
| --- | --- | --- | --- | --- | --- | --- | --- |
| **Prevalence of parental ACE score by sexual identity [% (95% CI)]** | | | | | | | |
| **Parental ACE score**  *(includes: Parental divorce. Parental psychological distress, Parental problem drinking, Parental recreational drug use, Domestic violence)* | **0** | 58.4  [57.2, 59.6] | 58.9 [57.6, 60.1] | 53.7 [49.5, 58.0] | 56.1 [49.4, 62.7] | | 54.4  [50.8, 57.9] |
|  | **1** | 27.8  [26.7, 28.9] | 27.6 [26.4, 28.8] | 29.3 [25.3, 33.3] | 29.4 [22.8,3 6.0] | | 29.3  [26.0, 32.6] |
|  | **≥2** | 13.8  [12.9, 14.7] | 13.5 [12.6, 14.4] | 17.0 [13.8, 20.3] | 14.6 [9.7, 19.4] | | 16.3  [13.6, 19.1] |
| **Prevalence of parenting ACE score by sexual identity [% (95% CI)]** | | | | | | | |
| **Parenting ACE score**  *(includes: Smacking, Harsh parenting)* | **0** | 37.7 [36.4, 38.9] | 37.7 [36.4, 38.9] | 40.5 [36.4, 44 5] | 36.1 [29.8, 42.4] | | 39.3 [35.9, 42.6] |
|  | **1** | 44.0 [42.7, 45.2] | 44.0 [42.7, 45.2] | 42.2 [38.1, 46.3] | 48.2 [41.7, 54.7] | | 43.9 [40.4, 47.4] |
|  | **2** | 18.4 [17.4, 19.3] | 18.4 [17.4, 19.3] | 17.3 [14.2, 20.4] | 15.7 [10.9, 20.5] | | 16.9 [14.3, 19.4] |

Table C - Associations between sexual identity and types of Adverse Childhood Experience (ACE) exposure in 8,686 adolescents aged 17 years from the Millennium Cohort Study (estimates are from multivariable multinomial logistic regression with adjustment for sex at birth, ethnicity, and childhood socioeconomic status)

| **Type of ACE** | | | | | |
| --- | --- | --- | --- | --- | --- |
| **Parenting ACEs** | **RRR [95% CI]** | **Parental ACEs** | **RRR [95% CI]** | **Bullying** | **RRR [95% CI]** |
| *Heterosexual* | *Reference* | *Heterosexual* | *Reference* | *Heterosexual* | *Reference* |
| Bisexual |  | Bisexual |  | Bisexual |  |
| 0 ACE | Reference | 0 ACE | Reference | No bullying | Reference |
| 1 ACE | 0.91 [0.72,1.14] | 1 ACE | **1.23 [1.00,1.53]** | Bullying | **2.11 [1.71,2.60]** |
|  |  |  |  |  |  |
| 2 ACEs | 0.97 [0.74,1.27] | 2 ACEs | **1.70 [1.26,2.29]** |  |  |
|  |  |  |  |  |  |
| Gay/Lesbian |  | Gay/Lesbian |  | Gay/Lesbian |  |
| 0 ACE | Reference | 0 ACE | Reference | No bullying | Reference |
| 1 ACE | 1.09 [0.79,1.51] | 1 ACE | 1.07 [0.75,1.53] | Bullying | **2.74 [1.97,3.80]** |
|  |  |  |  |  |  |
| 2 ACEs | 1.09 [0.72,1.67] | 2 ACEs | 1.33 [0.83,2.16] |  |  |

Estimates in bold indicate statistical significance i.e. 95% CIs does not include 1, RRR: Relative risk ratio.

**Table D - Associations between Adverse Childhood Experiences (ACEs) and mental health, general health, and health-risk behaviours in 8,686 adolescents from the Millennium Cohort Study (estimates are from logistic regression analysis adjusted for sex, ethnicity, and childhood socioeconomic status)**

|  | | **Odds ratio of outcome (95% CI)** | | | | | | | | | | | |
| --- | --- | --- | --- | --- | --- | --- | --- | --- | --- | --- | --- | --- | --- |
|  |  | (1) | (2) | (3) | (4) | (5) | (6) | (7) | (8) | (9) | (10) | (11) | (12) |
|  |  | **Psychological distress** | **SDQ-S** | | | | | **Doctor-diagnosed depression or anxiety** | **Self-harm** | **Suicidality** | **Victimization** | **Poor self-rated general health** | **Physical/mental health condition in past year** |
|  |  |  | **Conduct problems** | **Emotional symptoms** | **Hyperactivity/inattention** | **Peer problems** | **Prosocial behaviour difficulty** |  |  |  |  |  |  |
| Main effects – ACE score | 0 – Ref. | **-** | **-** | **-** | **-** | **-** | **-** | **-** | **-** | **-** |  |  |  |
|  | 1 | 1.30 [0.97,1.73] | 1.66 [0.99,2.78] | **1.27 [1.00,1.60]** | **1.41 [1.08,1.83]** | 1.22 [0.96,1.53] | 1.32 [0.99,1.75] | 1.14 [0.80,1.64] | 1.20 [0.93,1.56] | 1.38 [0.84,2.26] | **1.36 [1.15,1.60]** | 1.24 [0.83,1.86] | 1.05 [0.82,1.35] |
|  | 2 | **1.64 [1.21,2.23]** | **2.22 [1.34,3.69]** | **1.50 [1.19,1.90]** | **1.85 [1.41,2.42]** | **1.69 [1.32,2.17]** | **1.48 [1.11,1.97]** | **1.61 [1.14,2.26]** | **1.59 [1.23,2.05]** | **2.21 [1.38,3.54]** | **1.74 [1.47,2.06]** | **1.69 [1.09,2.62]** | **1.34 [1.05,1.73]** |
|  | ≥3 | **2.01 [1.51,2.68]** | **2.65 [1.64,4.30]** | **1.69 [1.32,2.16]** | **2.13 [1.65,2.76]** | **2.29 [1.83,2.87]** | 1.29 [0.96,1.75] | **2.54 [1.84,3.50]** | **2.29 [1.76,2.96]** | **3.86 [2.49,5.96]** | **2.01 [1.67,2.42]** | **2.26 [1.50,3.41]** | **1.80 [1.41,2.31]** |
| Main effects - sexuality | Heterosexual – Ref. | - | - | - | - | - | - | - | - | - |  |  |  |
|  | bisexual | **3.17 [1.76,5.70]** | 0.59 [0.10,3.61] | **3.69 [2.21,6.17]** | **2.23 [1.19,4.17]** | **2.39 [1.31,4.35]** | 0.48 [0.15,1.51] | **3.46 [1.71,7.01]** | **8.61 [5.17,14.34]** | **4.49 [1.79,11.24]** | **1.73 [1.07,2.80]** | **2.74 [1.13,6.66]** | 1.70 [0.86,3.34] |
|  | gay/lesbian | 2.60 [0.83,8.20] | 4.68 [0.77,28.45] | 2.02 [0.75,5.45] | 1.11 [0.27,4.51] | 1.98 [0.64,6.11] | 0.46 [0.08,2.78] | 2.43 [0.80,7.39] | **3.64 [1.53,8.67]** | **4.25 [1.16,15.53]** | 1.48 [0.61,3.56] | 1.79 [0.46,6.98] | 1.84 [0.67,5.00] |
| Interactions between ACE score and Sexuality | 1 # Heterosexual | - | - | - | - | - | - | - | - | - |  |  |  |
|  | 1 # bisexual | 1.56 [0.75,3.26] | 2.21 [0.26,18.48] | 0.81 [0.43,1.50] | 1.00 [0.48,2.07] | 1.37 [0.70,2.70] | 2.48 [0.64,9.57] | 1.20 [0.51,2.85] | 0.91 [0.49,1.69] | 1.36 [0.49,3.74] | 1.36 [0.75,2.46] | 0.66 [0.20,2.17] | 1.83 [0.82,4.10] |
|  | 1 # gay/lesb | 1.02 [0.26,3.96] | 0.31 [0.03,3.35] | 1.72 [0.52,5.63] | 1.37 [0.26,7.36] | 1.23 [0.34,4.42] | 2.19 [0.26,18.19] | 1.95 [0.54,7.08] | 1.45 [0.48,4.36] | 0.88 [0.19,4.15] | 1.16 [0.40,3.41] | 0.45 [0.07,2.95] | 1.31 [0.40,4.36] |
|  | 2 # Heterosexual | - | - | - | - | - | - | - | - | - |  |  |  |
|  | 2 # bisexual | 1.74 [0.81,3.73] | 5.00 [0.71,35.27] | 0.78 [0.39,1.56] | 1.10 [0.50,2.41] | 0.80 [0.38,1.71] | 3.00 [0.72,12.47] | 1.05 [0.45,2.43] | 0.66 [0.35,1.26] | 1.02 [0.37,2.84] | 1.02 [0.56,1.88] | 0.59 [0.19,1.80] | 2.19 [0.95,5.05] |
|  | 2 # gay/lesb | 1.01 [0.27,3.85] | 0.51 [0.07,3.98] | 1.60 [0.45,5.72] | 2.07 [0.46,9.39] | 1.08 [0.32,3.64] | 2.31 [0.29,18.73] | 1.31 [0.37,4.69] | 0.99 [0.33,2.99] | 0.64 [0.17,2.40] | 1.10 [0.41,2.90] | 2.01 [0.42,9.54] | 1.34 [0.40,4.51] |
|  | ≥3 # Heterosexual | - | - | - | - | - | - | - | - | - |  |  |  |
|  | ≥3 # bisexual | 1.33 [0.66,2.66] | 3.92 [0.60,25.52] | 0.74 [0.37,1.48] | 1.31 [0.65,2.63] | 1.02 [0.49,2.14] | 2.93 [0.87,9.86] | 0.76 [0.31,1.82] | 0.56 [0.30,1.03] | 0.77 [0.28,2.11] | 1.21 [0.66,2.21] | 0.72 [0.26,1.98] | 1.58 [0.70,3.55] |
|  | ≥3 # gay/lesb | 2.00 [0.53,7.53] | 0.31 [0.04,2.47] | 2.11 [0.64,6.97] | 2.56 [0.54,12.07] | 1.39 [0.40,4.85] | 2.67 [0.37,19.14] | 0.98 [0.27,3.55] | 1.23 [0.41,3.66] | 0.42 [0.10,1.84] | 2.34 [0.74,7.41] | 2.00 [0.42,9.47] | 0.71 [0.21,2.43] |
| Sex of study member | Male – Ref. | - | - | - | - | - | - | - | - | - |  |  |  |
|  | Female | **2.37 [2.00,2.81]** | **0.71 [0.57,0.90]** | **3.81 [3.29,4.40]** | 0.87 [0.74,1.02] | **1.17 [1.04,1.32]** | **0.27 [0.23,0.32]** | **2.53 [2.10,3.05]** | **1.70 [1.48,1.95]** | **2.60 [2.04,3.31]** | **0.83 [0.75,0.91]** | **1.30 [1.05,1.60]** | **1.17 [1.01,1.36]** |
| Childhood socioeconomic class (income class) | incomeq3=5 – Ref. | - | - | - | - | - | - | - | - | - |  |  |  |
|  | incomeq3=4 | 1.25 [0.98,1.61] | 1.33 [0.90,1.97] | 1.20 [0.98,1.48] | 0.96 [0.77,1.21] | **1.42 [1.15,1.75]** | 1.23 [0.94,1.61] | 1.22 [0.91,1.65] | 1.06 [0.86,1.31] | 1.28 [0.86,1.91] | 0.94 [0.80,1.11] | 1.33 [0.92,1.93] | 1.13 [0.90,1.42] |
|  | incomeq3=3 | **1.45 [1.12,1.87]** | 1.23 [0.85,1.79] | **1.29 [1.06,1.56]** | 1.07 [0.85,1.36] | **1.79 [1.44,2.23]** | 1.20 [0.91,1.59] | **1.57 [1.17,2.11]** | 1.08 [0.90,1.29] | 1.42 [0.95,2.12] | 0.93 [0.81,1.07] | **1.48 [1.04,2.10]** | 1.14 [0.91,1.43] |
|  | incomeq3=2 | **1.56 [1.21,2.01]** | **1.85 [1.32,2.59]** | **1.30 [1.04,1.62]** | 1.16 [0.91,1.49] | **2.31 [1.85,2.89]** | **1.58 [1.22,2.06]** | **1.45 [1.09,1.93]** | 1.21 [0.98,1.48] | **2.21 [1.55,3.14]** | 1.00 [0.85,1.16] | **1.76 [1.24,2.51]** | 1.16 [0.91,1.48] |
|  | incomeq3=1 | **1.90 [1.48,2.44]** | **2.18 [1.49,3.20]** | **1.37 [1.11,1.69]** | 1.03 [0.80,1.32] | **2.57 [2.06,3.20]** | **1.72 [1.32,2.24]** | **1.95 [1.49,2.56]** | **1.29 [1.02,1.63]** | **2.76 [1.92,3.97]** | 1.03 [0.88,1.21] | **2.99 [2.13,4.20]** | **1.54 [1.22,1.94]** |
| Ethnicity | White – Ref. | - | - | - | - | - | - | - | - | - |  |  |  |
|  | Ethnic minority | **0.69 [0.57,0.84]** | 0.80 [0.57,1.11] | **0.54 [0.43,0.67]** | **0.55 [0.43,0.70]** | **0.61 [0.52,0.72]** | 0.86 [0.69,1.06] | **0.41 [0.30,0.56]** | **0.63 [0.50,0.78]** | **0.57 [0.42,0.77]** | **0.60 [0.51,0.70]** | 1.00 [0.76,1.32] | **0.70 [0.56,0.87]** |

| Continuation of Supplementary Table 4 | | | | | | | | | | | | |
| --- | --- | --- | --- | --- | --- | --- | --- | --- | --- | --- | --- | --- |
|  | | Odds ratio of outcome (95% CI) | | | | | | | | | | |
|  |  | (13) | (14) | (15) | (16) | (17) | (18) | (19) | (20) | (21) | (22) | (23) |
|  |  | Poor quality of sleep in past month | Overweight/obese | Self-rated overweight/underweight | Regular smoking habit | Frequent binge drinking | Recreational drug use | Frequent cannabis use | Risky sex | Lack of exercise | Attempting to change weight | Anti-social behaviour |
| Main effects – ACE score | 0 – Ref. | - | - | - | - | - | - | - | - | - |  |  |
|  | 1 | 1.14 [0.92,1.41] | 1.10 [0.91,1.32] | 1.11 [0.93,1.33] | **1.40 [1.03,1.91]** | 1.13 [0.88,1.45] | **1.28 [1.07,1.54]** | 1.10 [0.82,1.46] | 1.20 [0.98,1.47] | 1.00 [0.80,1.24] | 1.01 [0.86,1.20] | **1.53 [1.27,1.84]** |
|  | 2 | **1.36 [1.10,1.69]** | 1.15 [0.93,1.41] | **1.35 [1.12,1.62]** | **1.84 [1.35,2.51]** | 1.18 [0.90,1.56] | **1.33 [1.11,1.59]** | 1.28 [0.92,1.77] | **1.35 [1.09,1.68]** | 1.08 [0.86,1.36] | **1.23 [1.03,1.47]** | **1.71 [1.39,2.11]** |
|  | ≥3 | **1.55 [1.23,1.95]** | **1.47 [1.18,1.82]** | **1.48 [1.21,1.81]** | **2.79 [2.08,3.75]** | 1.17 [0.87,1.58] | **1.62 [1.33,1.97]** | **1.70 [1.22,2.37]** | **1.82 [1.38,2.41]** | **1.26 [1.00,1.58]** | 1.22 [0.98,1.53] | **1.95 [1.57,2.42]** |
| Main effects - sexuality | Heterosexual – Ref. | - | - | - | - | - | - | - | - | - | - | **-** |
|  | bisexual | **2.53 [1.48,4.32]** | 1.31 [0.74,2.32] | 1.36 [0.79,2.34] | **3.15 [1.50,6.61]** | 0.94 [0.42,2.11] | 1.51 [0.88,2.61] | 1.14 [0.51,2.57] | 1.78 [0.96,3.29] | 0.96 [0.48,1.92] | 1.31 [0.78,2.21] | 1.36 [0.68,2.71] |
|  | gay/lesb | 0.76 [0.33,1.77] | 1.94 [0.83,4.51] | 1.96 [0.85,4.55] | 0.90 [0.20,4.05] | 0.52 [0.10,2.55] | 1.00 [0.39,2.59] | 0.67 [0.12,3.58] | 1.92 [0.65,5.69] | **2.95 [1.27,6.84]** | 0.93 [0.42,2.07] | 1.22 [0.40,3.75] |
| Interactions between ACE score and Sexuality | 1 # Heterosexual | - | - | - | - | - | - | - | - | - |  |  |
|  | 1 # bisexual | 0.76 [0.39,1.49] | 1.10 [0.54,2.24] | 1.34 [0.70,2.54] | 0.60 [0.22,1.61] | 1.32 [0.51,3.44] | 1.04 [0.52,2.08] | 1.25 [0.43,3.61] | 0.77 [0.36,1.67] | 1.30 [0.60,2.86] | 0.88 [0.45,1.72] | 0.92 [0.39,2.17] |
|  | 1 # gay/lesb | 2.03 [0.68,6.07] | 0.82 [0.26,2.52] | 1.45 [0.54,3.91] | 1.14 [0.20,6.47] | 2.15 [0.36,12.69] | 0.91 [0.29,2.88] | 1.04 [0.10,10.70] | 0.59 [0.16,2.12] | 0.41 [0.14,1.15] | 1.67 [0.56,4.96] | 0.66 [0.17,2.54] |
|  | 2 # Heterosexual | - | - | - | - | - | - | - | - | - |  |  |
|  | 2 # bisexual | 0.69 [0.34,1.42] | 1.02 [0.50,2.09] | 1.23 [0.58,2.59] | 0.53 [0.21,1.36] | 1.17 [0.43,3.22] | 1.23 [0.60,2.50] | 1.23 [0.48,3.19] | 0.71 [0.33,1.53] | 1.51 [0.66,3.43] | 0.97 [0.48,1.96] | 0.90 [0.38,2.12] |
|  | 2 # gay/lesb | 1.82 [0.61,5.44] | 0.68 [0.24,1.93] | 0.81 [0.27,2.40] | 1.56 [0.31,7.77] | 2.65 [0.44,16.10] | 1.34 [0.44,4.09] | 1.77 [0.26,12.10] | 0.80 [0.21,3.03] | 0.88 [0.29,2.66] | 1.23 [0.44,3.46] | 0.68 [0.17,2.65] |
|  | ≥3 # Heterosexual | - | - | - | - | - | - | - | - | - |  |  |
|  | ≥3 # bisexual | 0.83 [0.44,1.57] | 0.62 [0.30,1.29] | 1.08 [0.53,2.20] | 0.43 [0.18,1.04] | 1.35 [0.52,3.51] | 1.11 [0.57,2.16] | 1.37 [0.51,3.67] | 0.65 [0.29,1.43] | 1.62 [0.70,3.78] | 0.79 [0.41,1.51] | 1.36 [0.60,3.06] |
|  | ≥3 # gay/lesb | **3.43 [1.18,9.96]** | 1.00 [0.34,2.95] | 1.21 [0.40,3.69] | 1.30 [0.23,7.17] | 1.10 [0.12,10.11] | 1.63 [0.54,4.92] | 1.54 [0.22,10.98] | 0.88 [0.20,3.91] | 0.78 [0.27,2.27] | 2.49 [0.83,7.44] | 0.85 [0.22,3.31] |
| Sex of study member | Male – Ref. | - | - | - | - | - | - | - | - | - | **-** | **-** |
|  | Female | **1.24 [1.09,1.41]** | **1.31 [1.16,1.49]** | **1.33 [1.17,1.51]** | 0.98 [0.83,1.16] | 0.90 [0.77,1.06] | **0.74 [0.65,0.83]** | **0.58 [0.48,0.70]** | 0.90 [0.78,1.05] | **1.96 [1.74,2.20]** | **1.58 [1.39,1.78]** | **0.34 [0.30,0.39]** |
| Childhood socioeconomic class (income class) | incomeq3=5 – Ref. | - | - | - | - | - | - | - | - | - | - | - |
|  | incomeq3=4 | 1.11 [0.91,1.35] | **1.25 [1.01,1.55]** | 1.02 [0.85,1.22] | 1.04 [0.80,1.36] | 0.98 [0.77,1.25] | 0.88 [0.76,1.04] | 1.03 [0.79,1.33] | 1.05 [0.85,1.30] | 1.19 [0.97,1.44] | 0.93 [0.78,1.10] | 0.85 [0.71,1.03] |
|  | incomeq3=3 | 1.22 [1.00,1.48] | **1.51 [1.25,1.82]** | **1.20 [1.02,1.42]** | **1.37 [1.03,1.81]** | 0.96 [0.77,1.20] | 0.85 [0.71,1.02] | 0.95 [0.70,1.27] | 0.98 [0.80,1.21] | **1.44 [1.16,1.78]** | 1.11 [0.94,1.32] | 0.85 [0.71,1.01] |
|  | incomeq3=2 | **1.28 [1.05,1.56]** | **1.87 [1.55,2.26]** | **1.36 [1.14,1.63]** | **2.16 [1.67,2.79]** | **0.69 [0.53,0.89]** | 0.87 [0.71,1.07] | 1.06 [0.78,1.43] | 0.93 [0.73,1.18] | **1.86 [1.52,2.28]** | 1.07 [0.89,1.30] | 0.93 [0.77,1.13] |
|  | incomeq3=1 | **1.40 [1.12,1.73]** | **1.99 [1.61,2.46]** | **1.55 [1.27,1.88]** | **2.90 [2.28,3.70]** | **0.71 [0.54,0.94]** | **0.81 [0.67,0.98]** | 0.96 [0.71,1.30] | 0.97 [0.75,1.27] | **1.90 [1.55,2.33]** | 1.20 [0.97,1.49] | 0.89 [0.72,1.09] |
| Ethnicity | White – Ref. | - | - | - | - | - | - | - | - | - |  |  |
|  | Ethnic minority | **0.84 [0.71,0.99]** | 1.16 [0.97,1.37] | **1.18 [1.01,1.38]** | **0.28 [0.20,0.37]** | **0.64 [0.49,0.83]** | **0.60 [0.49,0.74]** | 0.85 [0.66,1.09] | **0.81 [0.67,0.98]** | 1.16 [0.97,1.38] | **1.26 [1.06,1.49]** | 1.08 [0.91,1.27] |

Text in bold: indicate 95% CIs that do not include 1.

Table E - Associations between Adverse Childhood Experiences (ACEs) and mental health, general health, and health-risk behaviour outcomes in 8,686 adolescents from the Millennium Cohort Study (estimates are predictive margins of interactions between ACEs and sexual identity from logistic regression models adjusted for sex, ethnicity, and childhood socioeconomic status)

|  | **Predictive margins* (95% CI)** | | | | | | | | | | |
| --- | --- | --- | --- | --- | --- | --- | --- | --- | --- | --- | --- |
|  | **Psychological distress** | **SDQ-S** | | | | | **Doctor-diagnosed depression or anxiety** | **Self-harm** | **Suicidality** | **Victimization** | **Self-rated general health** |
|  |  | **Conduct problems** | **Emotional symptoms** | **Hyperactivity/inattention** | **Peer problems** | **Prosocial behaviour difficulty** |  |  |  |  |  |
|  | (1) | (2) | (3) | (4) | (5) | (6) | (7) | (8) | (9) | (10) | (11) |
| 0 # Heterosexual | 0.08 [0.07,0.10] | 0.02 [0.02,0.03] | 0.15 [0.13,0.17] | 0.09 [0.07,0.10] | 0.12 [0.10,0.14] | 0.08 [0.06,0.09] | 0.06 [0.04,0.07] | 0.12 [0.10,0.15] | 0.03 [0.02,0.04] | 0.34 [0.31,0.36] | 0.04 [0.03,0.05] |
| 0 # Bisexual | 0.22 [0.14,0.30] | 0.03 [-0.00,0.05] | 0.36 [0.26,0.45] | 0.18 [0.10,0.25] | 0.24 [0.14,0.34] | 0.05 [0.01,0.08] | 0.14 [0.07,0.22] | 0.56 [0.45,0.67] | 0.10 [0.03,0.16] | 0.46 [0.34,0.57] | 0.08 [0.02,0.14] |
| 0 # Gay/Lesb | 0.17 [0.02,0.32] | 0.06 [-0.06,0.18] | 0.28 [0.12,0.45] | 0.06 [-0.01,0.13] | 0.23 [0.06,0.41] | 0.04 [-0.02,0.11] | 0.13 [0.01,0.25] | 0.35 [0.17,0.53] | 0.11 [-0.00,0.22] | 0.41 [0.21,0.60] | 0.07 [-0.01,0.14] |
| 1 # Heterosexual | 0.10 [0.09,0.12] | 0.04 [0.03,0.05] | 0.17 [0.15,0.19] | 0.12 [0.11,0.14] | 0.14 [0.12,0.15] | 0.11 [0.09,0.12] | 0.06 [0.05,0.07] | 0.16 [0.14,0.17] | 0.04 [0.03,0.05] | 0.41 [0.38,0.43] | 0.05 [0.04,0.06] |
| 1 # Bisexual | 0.35 [0.28,0.42] | 0.06 [0.01,0.10] | 0.35 [0.29,0.42] | 0.24 [0.17,0.31] | 0.35 [0.28,0.41] | 0.13 [0.07,0.19] | 0.21 [0.15,0.27] | 0.57 [0.50,0.64] | 0.19 [0.14,0.25] | 0.64 [0.56,0.71] | 0.09 [0.04,0.13] |
| 1 # Gay/Lesb | 0.24 [0.14,0.35] | 0.09 [0.01,0.16] | 0.37 [0.26,0.49] | 0.17 [0.07,0.28] | 0.30 [0.19,0.42] | 0.11 [0.03,0.19] | 0.25 [0.15,0.35] | 0.50 [0.38,0.63] | 0.14 [0.06,0.22] | 0.55 [0.42,0.68] | 0.03 [-0.01,0.06] |
| 2 # Heterosexual | 0.13 [0.11,0.14] | 0.05 [0.04,0.06] | 0.20 [0.18,0.22] | 0.15 [0.13,0.16] | 0.18 [0.16,0.20] | 0.11 [0.10,0.12] | 0.09 [0.08,0.11] | 0.20 [0.18,0.22] | 0.06 [0.04,0.07] | 0.48 [0.45,0.50] | 0.06 [0.05,0.07] |
| 2 # Bisexual | 0.44 [0.35,0.52] | 0.12 [0.06,0.18] | 0.42 [0.35,0.50] | 0.25 [0.17,0.32] | 0.29 [0.21,0.38] | 0.13 [0.05,0.20] | 0.29 [0.20,0.37] | 0.58 [0.49,0.67] | 0.23 [0.16,0.30] | 0.59 [0.50,0.67] | 0.11 [0.05,0.16] |
| 2 # Gay/Lesb | 0.28 [0.17,0.39] | 0.13 [0.04,0.21] | 0.42 [0.30,0.55] | 0.31 [0.18,0.43] | 0.28 [0.17,0.40] | 0.14 [0.05,0.22] | 0.19 [0.10,0.29] | 0.46 [0.33,0.59] | 0.14 [0.06,0.22] | 0.60 [0.49,0.72] | 0.20 [0.10,0.29] |
| ≥3 # Heterosexual | 0.16 [0.14,0.17] | 0.06 [0.05,0.08] | 0.22 [0.20,0.25] | 0.17 [0.15,0.19] | 0.24 [0.22,0.27] | 0.11 [0.09,0.12] | 0.13 [0.11,0.15] | 0.26 [0.23,0.28] | 0.10 [0.08,0.11] | 0.51 [0.47,0.54] | 0.08 [0.06,0.09] |
| ≥3 # Bisexual | 0.41 [0.34,0.48] | 0.14 [0.08,0.20] | 0.40 [0.32,0.48] | 0.40 [0.33,0.47] | 0.43 [0.35,0.51] | 0.15 [0.08,0.21] | 0.26 [0.19,0.33] | 0.62 [0.54,0.70] | 0.25 [0.18,0.32] | 0.69 [0.61,0.77] | 0.14 [0.09,0.19] |
| ≥3 # Gay/Lesb | 0.45 [0.31,0.60] | 0.08 [0.00,0.15] | 0.51 [0.36,0.67] | 0.36 [0.23,0.49] | 0.45 [0.31,0.60] | 0.10 [0.02,0.18] | 0.27 [0.15,0.39] | 0.58 [0.43,0.73] | 0.14 [0.05,0.24] | 0.76 [0.63,0.90] | 0.23 [0.10,0.35] |

|  | | | | | | | | | | | | |
| --- | --- | --- | --- | --- | --- | --- | --- | --- | --- | --- | --- | --- |
|  | **Predictive margins* (95% CI)** | | | | | | | | | | | |
|  | **Physical/mental health condition in past year** | **Poor quality of sleep in past month** | **Overweight/obese** | **Self-rated overweight/underweight** | **Regular smoking habit** | **Frequent binge drinking** | **Recreational drug use** | **Frequent cannabis use** | **Risky sex** | **Lack of exercise** | **Attempting to change weight** | **Anti-social behaviour** |
|  | (12) | (13) | (14) | (15) | (16) | (17) | (18) | (19) | (20) | (21) | (22) | (23) |
| 0 # Heterosexual | 0.13 [0.11,0.15] | 0.24 [0.22,0.26] | 0.24 [0.21,0.26] | 0.38 [0.35,0.41] | 0.07 [0.05,0.08] | 0.14 [0.12,0.16] | 0.24 [0.22,0.27] | 0.14 [0.11,0.16] | 0.31 [0.29,0.34] | 0.21 [0.18,0.23] | 0.56 [0.53,0.59] | 0.18 [0.16,0.20] |
| 0 # Bisexual | 0.21 [0.12,0.30] | 0.45 [0.34,0.57] | 0.30 [0.20,0.40] | 0.43 [0.31,0.55] | 0.17 [0.08,0.26] | 0.13 [0.06,0.20] | 0.31 [0.20,0.42] | 0.16 [0.07,0.25] | 0.49 [0.37,0.60] | 0.18 [0.09,0.27] | 0.62 [0.52,0.72] | 0.25 [0.13,0.36] |
| 0 # Gay/Lesb | 0.21 [0.05,0.37] | 0.19 [0.07,0.30] | 0.32 [0.17,0.48] | 0.50 [0.31,0.69] | 0.07 [-0.01,0.15] | 0.12 [-0.00,0.25] | 0.27 [0.11,0.44] | 0.04 [0.00,0.07] | 0.39 [0.19,0.59] | 0.42 [0.23,0.61] | 0.49 [0.33,0.66] | 0.22 [0.04,0.41] |
| 1 # Heterosexual | 0.12 [0.11,0.14] | 0.27 [0.25,0.28] | 0.25 [0.23,0.27] | 0.41 [0.39,0.43] | 0.09 [0.08,0.10] | 0.16 [0.14,0.17] | 0.30 [0.27,0.32] | 0.14 [0.12,0.16] | 0.37 [0.35,0.39] | 0.20 [0.18,0.22] | 0.57 [0.55,0.59] | 0.26 [0.24,0.27] |
| 1 # Bisexual | 0.33 [0.25,0.40] | 0.40 [0.33,0.47] | 0.35 [0.27,0.43] | 0.56 [0.48,0.65] | 0.17 [0.11,0.24] | 0.18 [0.12,0.24] | 0.42 [0.34,0.50] | 0.19 [0.12,0.27] | 0.49 [0.40,0.57] | 0.25 [0.18,0.32] | 0.59 [0.51,0.66] | 0.32 [0.24,0.41] |
| 1 # Gay/Lesb | 0.27 [0.16,0.38] | 0.39 [0.27,0.51] | 0.37 [0.25,0.50] | 0.63 [0.51,0.74] | 0.10 [0.04,0.16] | 0.19 [0.09,0.29] | 0.29 [0.18,0.40] | 0.20 [0.10,0.30] | 0.37 [0.25,0.49] | 0.25 [0.15,0.36] | 0.71 [0.59,0.82] | 0.22 [0.11,0.34] |
| 2 # Heterosexual | 0.16 [0.14,0.18] | 0.33 [0.31,0.35] | 0.26 [0.23,0.28] | 0.46 [0.44,0.48] | 0.11 [0.10,0.13] | 0.16 [0.14,0.17] | 0.31 [0.28,0.33] | 0.15 [0.13,0.17] | 0.40 [0.37,0.42] | 0.22 [0.19,0.24] | 0.62 [0.59,0.64] | 0.27 [0.25,0.29] |
| 2 # Bisexual | 0.37 [0.28,0.46] | 0.45 [0.36,0.54] | 0.30 [0.23,0.38] | 0.58 [0.49,0.67] | 0.19 [0.12,0.26] | 0.15 [0.09,0.21] | 0.46 [0.36,0.55] | 0.20 [0.13,0.27] | 0.46 [0.37,0.55] | 0.27 [0.18,0.35] | 0.69 [0.61,0.77] | 0.27 [0.20,0.35] |
| 2 # Gay/Lesb | 0.31 [0.19,0.43] | 0.40 [0.26,0.53] | 0.29 [0.18,0.40] | 0.60 [0.47,0.73] | 0.14 [0.07,0.22] | 0.16 [0.07,0.25] | 0.33 [0.22,0.45] | 0.16 [0.07,0.24] | 0.43 [0.30,0.56] | 0.40 [0.26,0.53] | 0.63 [0.49,0.76] | 0.26 [0.16,0.37] |
| ≥3 # Heterosexual | 0.21 [0.18,0.23] | 0.36 [0.33,0.39] | 0.32 [0.29,0.35] | 0.48 [0.45,0.51] | 0.16 [0.14,0.18] | 0.15 [0.13,0.18] | 0.34 [0.30,0.37] | 0.19 [0.17,0.22] | 0.44 [0.41,0.47] | 0.24 [0.21,0.26] | 0.62 [0.59,0.65] | 0.29 [0.26,0.32] |
| ≥3 # Bisexual | 0.43 [0.35,0.52] | 0.55 [0.47,0.63] | 0.26 [0.18,0.34] | 0.59 [0.49,0.68] | 0.18 [0.12,0.25] | 0.17 [0.10,0.25] | 0.43 [0.35,0.52] | 0.25 [0.18,0.32] | 0.46 [0.37,0.54] | 0.33 [0.25,0.41] | 0.68 [0.61,0.75] | 0.42 [0.35,0.50] |
| ≥3 # Gay/Lesb | 0.26 [0.14,0.37] | 0.59 [0.45,0.73] | 0.47 [0.33,0.61] | 0.70 [0.56,0.84] | 0.18 [0.07,0.29] | 0.14 [0.04,0.24] | 0.47 [0.32,0.62] | 0.25 [0.13,0.38] | 0.62 [0.48,0.75] | 0.40 [0.26,0.54] | 0.80 [0.68,0.91] | 0.26 [0.13,0.40] |

*Predictive margins (or adjusted predictive margins or the predicted probabilities) for each outcome in each category generated by interactions between the sexual identity and ACE variables.

Table F - Associations between Adverse Childhood Experiences (ACEs) and mental health in 8,686 adolescents from the Millennium Cohort Study (estimates are from linear regression analysis adjusted for sex, ethnicity, and childhood socioeconomic status)

Warwick–Edinburgh Mental Well-being Scale (WEMWBS)

Rosenberg Self-Esteem Scale (5-item). Text in bold: indicate 95% CIs that do not include 0.

|  | | **Change in score [β (95% CI)]** | | | | | | | | |
| --- | --- | --- | --- | --- | --- | --- | --- | --- | --- | --- |
|  |  | **(1)** | **(2)** | **(3)** | **(4)** | **(5)** | **(6)** | **(7)** | **(8)** | **(9)** |
|  |  | **Psychological distress**  **(score/24)** | **SDQ-Subscales (score/10)** | | | | | **Mental wellbeing** | **Self-esteem**  **(score/15)** | **BMI** |
|  |  |  | **Emotional symptoms** | **Conduct problems** | **Hyperactivity/inattention** | **Peer problems** | **Prosocial behaviour difficulty** |  |  |  |
| Main effects – ACE score | 0 – Ref. | - | - | - | - | - | - | - | - | - |
|  | 1 | **0.44 [0.09,0.79]** | 0.17 [-0.01,0.35] | **0.23 [0.13,0.32]** | **0.32 [0.15,0.49]** | **0.13 [0.00,0.26]** | **-0.18 [-0.31,-0.05]** | **-0.48 [-0.80,-0.17]** | -0.16 [-0.40,0.08] | 0.20 [-0.12,0.52] |
|  | 2 | **1.07 [0.66,1.49]** | **0.34 [0.14,0.53]** | **0.43 [0.31,0.54]** | **0.64 [0.46,0.83]** | **0.35 [0.22,0.47]** | **-0.23 [-0.37,-0.09]** | **-0.90 [-1.26,-0.55]** | **-0.47 [-0.71,-0.22]** | **0.42 [0.06,0.78]** |
|  | ≥3 | **1.46 [1.09,1.84]** | **0.58 [0.38,0.79]** | **0.57 [0.45,0.70]** | **0.73 [0.55,0.92]** | **0.67 [0.53,0.81]** | **-0.32 [-0.47,-0.18]** | **-1.14 [-1.47,-0.81]** | **-0.76 [-1.02,-0.51]** | **1.02 [0.63,1.42]** |
| Main effects - sexuality | Heterosexual – Ref. | - | - | - | - | - | - | - | - | - |
|  | Bisexual | **3.25 [2.25,4.25]** | **1.45 [0.95,1.94]** | 0.11 [-0.23,0.44] | **0.66 [0.13,1.19]** | **0.6 [0.27,1.06]** | -0.14 [-0.52,0.23] | **-1.56 [-2.38,-0.75]** | **-1.01 [-1.61,-0.41]** | 0.34 [-0.55,1.24] |
|  | Gay/Lesbian | 1.89 [-0.32,4.11] | 0.85 [-0.11,1.80] | 0.18 [-0.66,1.03] | 0.11 [-0.84,1.05] | 0.54 [-0.26,1.35] | -0.28 [-0.81,0.25] | **-1.76 [-3.18,-0.34]** | -1.12 [-2.59,0.36] | 1.27 [-1.04,3.57] |
| Interactions between ACE score and Sexuality | 1 # Hetero – Ref. | - | - | - | - | - | - | - | - | - |
|  | 1 # Bisexual | 0.66 [-0.72,2.03] | 0.04 [-0.61,0.69] | 0.20 [-0.24,0.64] | 0.07 [-0.65,0.79] | 0.34 [-0.13,0.81] | 0.03 [-0.47,0.53] | -0.19 [-1.25,0.88] | -0.78 [-1.65,0.09] | 0.26 [-1.01,1.54] |
|  | 1 # Gay/Lesb | 1.50 [-1.15,4.15] | 0.69 [-0.50,1.89] | -0.11 [-1.05,0.83] | 0.80 [-0.38,1.98] | 0.23 [-0.75,1.22] | 0.36 [-0.35,1.07] | -0.37 [-2.11,1.37] | -0.42 [-2.26,1.42] | -0.20 [-3.20,2.79] |
|  | 2 # Hetero – Ref. | - | - | - | - | - | - | - | - | - |
|  | 2 # Bisexual | 1.13 [-0.39,2.65] | 0.01 [-0.72,0.73] | **0.57 [0.05,1.09]** | 0.54 [-0.18,1.26] | 0.14 [-0.40,0.69] | -0.02 [-0.61,0.56] | -0.36 [-1.55,0.83] | -0.38 [-1.31,0.56] | -0.21 [-1.48,1.05] |
|  | 2 # Gay/Lesb | 0.78 [-1.83,3.39] | 0.67 [-0.56,1.89] | 0.46 [-0.62,1.53] | 0.54 [-0.69,1.76] | 0.08 [-0.88,1.04] | 0.11 [-0.77,0.99] | 0.39 [-1.66,2.44] | 0.01 [-1.98,2.00] | -0.84 [-3.59,1.91] |
|  | ≥3 # Hetero – Ref. | - | - | - | - | - | - | - | - | - |
|  | ≥3 # Bisexual | 1.15 [-0.21,2.51] | 0.11 [-0.60,0.83] | **0.69 [0.19,1.20]** | **0.73 [0.03,1.42]** | 0.36 [-0.15,0.88] | -0.01 [-0.49,0.48] | -0.26 [-1.29,0.77] | -0.67 [-1.49,0.16] | -0.78 [-2.16,0.61] |
|  | ≥3 # Gay/Lesb | **3.30 [0.67,5.92]** | 1.04 [-0.12,2.20] | 0.18 [-0.79,1.15] | **1.27 [0.04,2.50]** | 0.64 [-0.34,1.61] | -0.14 [-0.86,0.58] | -0.62 [-2.34,1.11] | -1.36 [-3.20,0.48] | 0.31 [-2.69,3.31] |
| Sex of study member | Male – Ref. | - | - | - | - | - | - | - | - | - |
|  | Female | **1.80 [1.58,2.02]** | **1.52 [1.39,1.65]** | **-0.29 [-0.37,-0.21]** | **-0.28 [-0.40,-0.16]** | **0.10 [0.02,0.18]** | **0.98 [0.89,1.06]** | **-1.21 [-1.40,-1.01]** | **-0.87 [-1.02,-0.72]** | **0.73 [0.48,0.97]** |
| Childhood socioeconomic class (income class) | IncomeQ3=5 – Ref. | - | - | - | - | - | - | - | - | - |
|  | IncomeQ3=4 | 0.28 [-0.08,0.63] | **0.27 [0.09,0.45]** | 0.02 [-0.09,0.13] | 0.05 [-0.14,0.24] | **0.28 [0.17,0.38]** | -0.06 [-0.19,0.08] | -0.26 [-0.55,0.04] | -0.16 [-0.37,0.04] | **0.47 [0.11,0.84]** |
|  | IncomeQ3=3 | **0.39 [0.06,0.72]** | **0.30 [0.14,0.46]** | 0.03 [-0.07,0.13] | 0.15 [-0.03,0.33] | **0.42 [0.31,0.54]** | -0.11 [-0.24,0.01] | **-0.42 [-0.73,-0.11]** | **-0.60 [-0.84,-0.36]** | **0.76 [0.41,1.11]** |
|  | IncomeQ3=2 | **0.43 [0.07,0.80]** | **0.30 [0.12,0.48]** | **0.25 [0.13,0.37]** | **0.37 [0.18,0.55]** | **0.71 [0.58,0.84]** | **-0.19 [-0.32,-0.05]** | **-0.37 [-0.67,-0.07]** | **-0.32 [-0.55,-0.09]** | **1.14 [0.73,1.55]** |
|  | IncomeQ3=1 | **0.71 [0.31,1.11]** | **0.47 [0.29,0.66]** | **0.30 [0.18,0.42]** | **0.31 [0.11,0.50]** | **0.82 [0.69,0.96]** | **-0.18 [-0.32,-0.05]** | **-0.70 [-1.04,-0.36]** | **-0.59 [-0.83,-0.35]** | **1.19 [0.75,1.62]** |
| Ethnicity | White – Ref. | - | - | - | - | - | - | - | - | - |
|  | Ethnic minority | **-0.70 [-1.00,-0.40]** | **-0.66 [-0.81,-0.51]** | -0.05 [-0.16,0.05] | **-0.59 [-0.75,-0.43]** | **-0.40 [-0.50,-0.29]** | **0.18 [0.07,0.30]** | **0.48 [0.22,0.74]** | **0.53 [0.32,0.74]** | 0.19 [-0.20,0.58] |

**Table G - Associations between Adverse Childhood Experiences (ACEs) and mental health outcomes in 8,686 adolescents from the Millennium Cohort Study (estimates are predictive margins of interactions between ACEs and Sexuality from linear regression analysis adjusted for sex, ethnicity, and childhood socioeconomic status)**

|  | **Predictive margins [95% CI]** | | | | | | | | |
| --- | --- | --- | --- | --- | --- | --- | --- | --- | --- |
|  | **(1)** | **(2)** | **(3)** | **(4)** | **(5)** | **(6)** | **(7)** | **(8)** | **(9)** |
|  | **Psychological distress**  **(score/24)** | **SDQ-Subscales (score/10)** | | | | | **Mental wellbeing** | **Self-esteem**  **(score/15)** | **BMI** |
|  |  | **Emotional symptoms** | **Conduct problems** | **Hyperactivity/inattention** | **Peer problems** | **Prosocial behaviour difficulty** |  |  |  |
| 0 # Heterosexual | 5.83 [5.55,6.11] | 2.96 [2.83,3.09] | 1.29 [1.22,1.37] | 3.37 [3.24,3.51] | 1.71 [1.62,1.80] | 8.07 [7.97,8.17] | 16.44 [16.19,16.68] | 10.59 [10.40,10.78] | 22.79 [22.55,23.03] |
| 0 # Bisexual | 9.08 [8.14,10.02] | 4.41 [3.93,4.88] | 1.40 [1.08,1.73] | 4.03 [3.52,4.55] | 2.37 [1.98,2.76] | 7.93 [7.56,8.29] | 14.87 [14.09,15.65] | 9.58 [8.99,10.17] | 23.13 [22.27,24.00] |
| 0 # Gay/Lesbian | 7.72 [5.53,9.92] | 3.81 [2.86,4.75] | 1.48 [0.64,2.32] | 3.48 [2.55,4.41] | 2.25 [1.46,3.05] | 7.79 [7.27,8.31] | 14.68 [13.28,16.08] | 9.48 [8.03,10.93] | 24.06 [21.78,26.33] |
| 1 # Heterosexual | 6.28 [6.07,6.49] | 3.13 [3.02,3.24] | 1.52 [1.45,1.58] | 3.69 [3.58,3.80] | 1.84 [1.76,1.92] | 7.89 [7.81,7.97] | 15.95 [15.75,16.15] | 10.43 [10.28,10.58] | 22.99 [22.78,23.19] |
| 1 # Bisexual | 10.18 [9.32,11.04] | 4.61 [4.18,5.05] | 1.83 [1.57,2.08] | 4.42 [4.01,4.83] | 2.84 [2.54,3.15] | 7.78 [7.50,8.05] | 14.20 [13.56,14.84] | 8.64 [8.04,9.24] | 23.59 [22.66,24.52] |
| 1 # Gay/Lesbian | 9.67 [8.35,10.99] | 4.67 [4.00,5.34] | 1.59 [1.21,1.97] | 4.59 [4.01,5.18] | 2.62 [2.07,3.17] | 7.97 [7.51,8.43] | 13.82 [12.91,14.74] | 8.89 [7.90,9.88] | 24.05 [22.40,25.71] |
| 2 # Heterosexual | 6.91 [6.65,7.17] | 3.30 [3.16,3.44] | 1.72 [1.63,1.80] | 4.02 [3.89,4.14] | 2.06 [1.96,2.15] | 7.84 [7.74,7.93] | 15.53 [15.31,15.76] | 10.12 [9.97,10.28] | 23.21 [22.95,23.47] |
| 2 # Bisexual | 11.29 [10.30,12.28] | 4.75 [4.29,5.21] | **2.40 [2.04,2.76]** | 5.22 [4.78,5.65] | 2.86 [2.48,3.24] | 7.67 [7.26,8.07] | 13.61 [12.83,14.39] | 8.74 [8.03,9.44] | 23.34 [22.41,24.26] |
| 2 # Gay/Lesbian | 9.58 [8.30,10.86] | 4.81 [4.13,5.49] | 2.36 [1.76,2.96] | 4.66 [3.87,5.45] | 2.68 [2.16,3.20] | 7.67 [7.11,8.22] | 14.16 [12.92,15.40] | 9.02 [7.96,10.07] | 23.64 [21.89,25.38] |
| ≥3 # Heterosexual | 7.30 [7.03,7.56] | 3.54 [3.40,3.69] | 1.87 [1.77,1.96] | 4.11 [3.97,4.25] | 2.38 [2.27,2.49] | 7.75 [7.64,7.85] | 15.30 [15.07,15.52] | 9.83 [9.64,10.02] | 23.81 [23.50,24.12] |
| ≥3 # Bisexual | 11.70 [10.86,12.54] | 5.10 [4.64,5.56] | **2.67 [2.29,3.05]** | **5.50 [5.10,5.89]** | 3.41 [3.07,3.74] | 7.60 [7.27,7.93] | 13.47 [12.82,14.13] | 8.15 [7.61,8.70] | 23.38 [22.41,24.35] |
| ≥3 # Gay/Lesbian | **12.48 [11.01,13.95]** | 5.43 [4.68,6.18] | 2.23 [1.70,2.76] | **5.48 [4.73,6.24]** | 3.56 [3.01,4.11] | 7.33 [6.85,7.82] | 12.92 [11.97,13.87] | 7.36 [6.28,8.43] | 25.39 [23.68,27.10] |

Warwick–Edinburgh Mental Well-being Scale (WEMWBS)

Rosenberg Self-Esteem Scale (5-item)

Table H - Associations between Parental Adverse Childhood Experiences (ACEs) and mental health, general health, and health-risk behaviours in 8,686 adolescents from the Millennium Cohort Study (estimates are from logistic regression analysis adjusted for sex, ethnicity, and childhood socioeconomic status)

|  | | **Odds ratio of outcome (95% CI)** | | | | | | | | | | | |
| --- | --- | --- | --- | --- | --- | --- | --- | --- | --- | --- | --- | --- | --- |
|  |  | **(1)** | **(2)** | **(3)** | **(4)** | **(5)** | **(6)** | **(7)** | **(8)** | **(9)** | **(10)** | **(11)** | **(12)** |
|  |  | **Psychological distress** | **SDQ-S** | | | | | **Doctor-diagnosed depression or anxiety** | **Self-harm** | **Suicidality** | **Victimization** | **Poor self-rated general health** | **Physical/mental health condition in past year** |
|  |  |  | **Conduct problems** | **Emotional symptoms** | **Hyperactivity/inattention** | **Peer problems** | **Prosocial behaviour difficulty** |  |  |  |  |  |  |
| Main effects – Parental ACE score | 0 – Ref. | - | - | - | - | - | - | - | - | - |  |  |  |
|  | 1 | **1.53 [1.26,1.86]** | **1.62 [1.17,2.23]** | **1.34 [1.12,1.59]** | 1.14 [0.93,1.41] | **1.37 [1.15,1.63]** | 0.96 [0.78,1.19] | **1.75 [1.37,2.24]** | **1.39 [1.17,1.65]** | **1.72 [1.30,2.27]** | **1.24 [1.09,1.41]** | **1.53 [1.13,2.07]** | **1.41 [1.17,1.70]** |
|  | ≥2 | **1.72 [1.36,2.17]** | 1.51 [1.00,2.29] | **1.37 [1.08,1.74]** | 1.25 [0.96,1.62] | **1.63 [1.31,2.04]** | 0.93 [0.67,1.29] | **2.56 [1.94,3.37]** | **1.88 [1.53,2.29]** | **2.73 [1.96,3.79]** | **1.36 [1.14,1.63]** | **2.09 [1.47,2.97]** | **1.57 [1.20,2.05]** |
| Main effects - sexuality | Heterosexual – Ref. | - | - | - | - | - | - | - | - | - |  |  |  |
|  | bisexual | **5.15 [3.98,6.67]** | **2.08 [1.21,3.57]** | **3.90 [2.96,5.15]** | **2.41 [1.73,3.35]** | **3.11 [2.33,4.16]** | 1.07 [0.66,1.73] | **4.33 [3.19,5.88]** | **7.63 [5.74,10.14]** | **5.43 [3.56,8.29]** | **1.95 [1.50,2.54]** | **2.51 [1.60,3.94]** | **2.67 [1.97,3.61]** |
|  | gay/lesbian | **2.84 [1.81,4.44]** | **3.10 [1.47,6.54]** | **3.52 [2.38,5.21]** | **1.86 [1.11,3.14]** | **2.09 [1.32,3.30]** | 1.13 [0.60,2.14] | **3.68 [2.25,6.02]** | **4.72 [3.24,6.87]** | **2.98 [1.51,5.88]** | **1.65 [1.12,2.44]** | **2.13 [1.12,4.04]** | **2.53 [1.63,3.93]** |
| Interactions between Parental ACE score and Sexuality | 1 # Heterosexual | - | - | - | - | - | - | - | - | - |  |  |  |
|  | 1 # bisexual | 0.74 [0.47,1.16] | 1.00 [0.40,2.46] | 0.48 [0.28,0.83] | 0.94 [0.54,1.63] | 0.61 [0.36,1.03] | 1.25 [0.54,2.88] | 0.70 [0.40,1.23] | 0.68 [0.43,1.08] | 0.79 [0.43,1.45] | 1.07 [0.67,1.71] | 0.61 [0.29,1.31] | 1.29 [0.78,2.15] |
|  | 1 # gay/lesb | 1.23 [0.52,2.89] | 0.41 [0.10,1.72] | 0.88 [0.38,2.05] | 1.37 [0.58,3.23] | 1.34 [0.61,2.95] | 1.09 [0.34,3.44] | 0.90 [0.38,2.15] | 0.94 [0.45,1.96] | 1.02 [0.37,2.82] | 1.29 [0.61,2.75] | 1.47 [0.51,4.24] | 0.67 [0.30,1.49] |
|  | ≥2 # Heterosexual | - | - | - | - | - | - | - | - | - |  |  |  |
|  | ≥2 # bisexual | 0.90 [0.49,1.63] | 1.06 [0.37,2.98] | 0.77 [0.41,1.47] | 1.44 [0.79,2.64] | 0.79 [0.42,1.49] | 1.52 [0.66,3.52] | 0.56 [0.31,1.02] | 0.76 [0.43,1.33] | 0.66 [0.34,1.30] | 1.21 [0.69,2.12] | 0.65 [0.28,1.52] | 1.02 [0.56,1.87] |
|  | ≥2 # gay/lesb | 1.94 [0.69,5.48] | 0.43 [0.06,2.92] | 1.11 [0.39,3.22] | 1.47 [0.52,4.15] | 1.62 [0.60,4.32] | 0.53 [0.11,2.48] | 0.70 [0.23,2.13] | 0.79 [0.31,1.98] | 0.77 [0.19,3.09] | 2.93 [0.87,9.85] | 1.65 [0.48,5.61] | 0.51 [0.15,1.66] |
| Sex of study member | Male – Ref. | - | - | - | - | - | - | - | - | - |  |  |  |
|  | Female | **2.29 [1.94,2.70]** | **0.68 [0.54,0.86]** | **3.69 [3.20,4.27]** | **0.83 [0.71,0.98]** | 1.12 [1.00,1.26] | **0.27 [0.23,0.32]** | **2.42 [2.00,2.91]** | **1.63 [1.42,1.87]** | **2.44 [1.92,3.09]** | **0.80 [0.73,0.88]** | **1.24 [1.01,1.53]** | 1.13 [0.98,1.31] |
| Childhood socioeconomic class (income class) | incomeq3=5 – Ref. | - | - | - | - | - | - | - | - | - |  |  |  |
|  | incomeq3=4 | **1.28 [1.00,1.63]** | 1.37 [0.93,2.02] | 1.22 [1.00,1.50] | 0.99 [0.79,1.24] | **1.45 [1.17,1.78]** | 1.24 [0.95,1.63] | 1.24 [0.92,1.67] | 1.07 [0.86,1.31] | 1.31 [0.88,1.97] | 0.95 [0.81,1.12] | 1.34 [0.93,1.94] | 1.15 [0.91,1.44] |
|  | incomeq3=3 | **1.46 [1.13,1.88]** | 1.27 [0.88,1.84] | **1.29 [1.06,1.57]** | 1.10 [0.87,1.40] | **1.82 [1.46,2.27]** | 1.22 [0.92,1.60] | **1.57 [1.17,2.11]** | 1.08 [0.90,1.29] | 1.45 [0.97,2.17] | 0.94 [0.82,1.08] | **1.48 [1.05,2.10]** | 1.15 [0.92,1.45] |
|  | incomeq3=2 | **1.56 [1.21,2.00]** | **1.95 [1.39,2.74]** | **1.32 [1.06,1.63]** | 1.24 [0.96,1.59] | **2.39 [1.90,3.01]** | **1.63 [1.25,2.13]** | **1.43 [1.07,1.90]** | 1.21 [0.98,1.49] | **2.25 [1.58,3.20]** | 1.02 [0.88,1.19] | **1.77 [1.24,2.53]** | 1.18 [0.93,1.51] |
|  | incomeq3=1 | **1.88 [1.47,2.40]** | **2.28 [1.56,3.32]** | **1.37 [1.12,1.69]** | 1.09 [0.85,1.40] | **2.62 [2.09,3.29]** | **1.77 [1.35,2.33]** | **1.88 [1.43,2.48]** | **1.28 [1.01,1.61]** | **2.76 [1.91,3.99]** | 1.05 [0.89,1.23] | **2.92 [2.06,4.12]** | **1.55 [1.23,1.96]** |
| Ethnicity | White – Ref. | - | - | - | - | - | - | - | - | - |  |  |  |
|  | Ethnic minority | **0.69 [0.56,0.84]** | 0.80 [0.57,1.11] | **0.54 [0.44,0.67]** | **0.55 [0.43,0.71]** | **0.61 [0.52,0.72]** | 0.87 [0.70,1.07] | **0.41 [0.30,0.56]** | **0.63 [0.50,0.78]** | **0.57 [0.42,0.78]** | **0.60 [0.52,0.70]** | 0.99 [0.76,1.30] | **0.70 [0.56,0.87]** |

| Continuation of Supplementary Table 8 | | | | | | | | | | | | |
| --- | --- | --- | --- | --- | --- | --- | --- | --- | --- | --- | --- | --- |
|  | | Odds ratio of outcome (95% CI) | | | | | | | | | | |
|  |  | (13) | (14) | (15) | (16) | (17) | (18) | (19) | (20) | (21) | (22) | (23) |
|  |  | Poor quality of sleep in past month | Overweight/obese | Self-rated overweight/underweight | Regular smoking habit | Frequent binge drinking | Recreational drug use | Frequent cannabis use | Risky sex | Lack of exercise | Attempting to change weight | Anti-social behaviour |
| Main effects – Parental ACE score | 0 – Ref. | - | - | - | - | - | - | - | - | - |  |  |
|  | 1 | **1.30 [1.11,1.51]** | 1.01 [0.86,1.19] | 1.14 [0.99,1.32] | **1.58 [1.28,1.96]** | 1.09 [0.87,1.37] | 1.16 [1.00,1.35] | 1.24 [0.99,1.54] | **1.27 [1.06,1.52]** | 1.11 [0.94,1.30] | 1.05 [0.90,1.22] | 1.08 [0.92,1.26] |
|  | ≥2 | **1.46 [1.15,1.84]** | **1.30 [1.06,1.58]** | **1.36 [1.11,1.65]** | **2.41 [1.85,3.13]** | 1.18 [0.87,1.60] | **1.64 [1.36,1.99]** | **1.48 [1.10,1.99]** | **1.70 [1.33,2.17]** | **1.28 [1.04,1.58]** | 1.07 [0.86,1.34] | 1.18 [0.95,1.46] |
| Main effects - sexuality | Heterosexual – Ref. | - | - | - | - | - | - | - | - | - | - | **-** |
|  | bisexual | **2.05 [1.56,2.70]** | 1.26 [0.91,1.75] | **1.57 [1.18,2.10]** | **2.40 [1.66,3.47]** | 1.20 [0.79,1.82] | **1.58 [1.21,2.06]** | 1.45 [0.96,2.17] | **1.52 [1.06,2.19]** | 1.34 [0.95,1.88] | 1.16 [0.89,1.50] | 1.29 [0.93,1.80] |
|  | gay/lesb | 1.29 [0.85,1.95] | **1.78 [1.14,2.76]** | **2.15 [1.40,3.30]** | 0.98 [0.49,1.94] | 1.02 [0.54,1.94] | 1.09 [0.69,1.74] | 0.73 [0.32,1.67] | 1.37 [0.79,2.37] | 2.04 [1.30,3.22] | 1.25 [0.80,1.95] | 0.80 [0.46,1.38] |
| Interactions between Parental ACE score and Sexuality | 1 # Heterosexual | - | - | - | - | - | - | - | - | - |  |  |
|  | 1 # bisexual | 0.84 [0.51,1.38] | 1.11 [0.65,1.89] | 1.21 [0.74,1.99] | 0.53 [0.27,1.04] | 0.83 [0.42,1.64] | 0.97 [0.59,1.59] | 0.91 [0.45,1.83] | 0.71 [0.39,1.32] | 0.86 [0.49,1.50] | 1.04 [0.64,1.67] | 1.10 [0.60,2.01] |
|  | 1 # gay/lesb | 1.57 [0.72,3.40] | 0.75 [0.34,1.66] | 1.08 [0.47,2.52] | 1.70 [0.58,4.93] | 0.81 [0.25,2.59] | 1.49 [0.64,3.48] | 1.23 [0.34,4.45] | 1.25 [0.50,3.12] | 1.18 [0.55,2.52] | 1.15 [0.46,2.85] | 1.55 [0.62,3.85] |
|  | ≥2ACEs / Heterosexual | - | - | - | - | - | - | - | - | - |  |  |
|  | ≥2 # bisexual | 1.24 [0.69,2.23] | 0.66 [0.35,1.27] | 0.85 [0.44,1.63] | 0.62 [0.31,1.25] | 1.10 [0.50,2.43] | 1.38 [0.76,2.52] | 1.13 [0.52,2.44] | 0.87 [0.45,1.67] | 1.26 [0.66,2.42] | 1.05 [0.55,1.99] | 1.56 [0.83,2.94] |
|  | ≥2 # gay/lesb | 2.03 [0.76,5.47] | 1.11 [0.42,2.96] | 1.09 [0.37,3.17] | 1.21 [0.31,4.69] | 0.98 [0.22,4.36] | 1.14 [0.40,3.26] | 2.86 [0.73,11.22] | 1.31 [0.39,4.42] | 0.85 [0.31,2.35] | 2.94 [0.84,10.33] | 1.34 [0.40,4.47] |
| Sex of study member | Male – Ref. | - | - | - | - | - | - | - | - | - | **-** | **-** |
|  | Female | **1.21 [1.07,1.37]** | **1.29 [1.14,1.47]** | **1.30 [1.15,1.48]** | 0.93 [0.79,1.10] | 0.90 [0.76,1.05] | **0.72 [0.64,0.81]** | **0.56 [0.47,0.69]** | 0.88 [0.76,1.02] | **1.93 [1.73,2.16]** | **1.56 [1.38,1.76]** | **0.33 [0.29,0.38]** |
| Childhood socioeconomic class (income class) | incomeq3=5 – Ref. | - | - | - | - | - | - | - | - | - | - | - |
|  | incomeq3=4 | 1.10 [0.91,1.35] | **1.25 [1.01,1.55]** | 1.02 [0.85,1.23] | 1.05 [0.81,1.37] | 0.99 [0.77,1.26] | 0.89 [0.76,1.04] | 1.04 [0.80,1.35] | 1.05 [0.85,1.30] | 1.19 [0.98,1.46] | 0.93 [0.79,1.10] | 0.86 [0.72,1.04] |
|  | incomeq3=3 | 1.20 [0.98,1.47] | **1.52 [1.26,1.82]** | **1.21 [1.03,1.43]** | **1.35 [1.02,1.79]** | 0.96 [0.77,1.20] | 0.84 [0.70,1.01] | 0.95 [0.71,1.28] | 0.98 [0.79,1.21] | **1.45 [1.17,1.79]** | 1.12 [0.94,1.33] | 0.86 [0.72,1.02] |
|  | incomeq3=2 | **1.26 [1.03,1.54]** | **1.89 [1.56,2.28]** | **1.37 [1.15,1.63]** | **2.12 [1.63,2.75]** | **0.69 [0.53,0.89]** | 0.85 [0.70,1.04] | 1.07 [0.79,1.45] | 0.92 [0.72,1.17] | **1.88 [1.54,2.29]** | 1.08 [0.90,1.31] | 0.97 [0.80,1.17] |
|  | incomeq3=1 | **1.36 [1.09,1.69]** | **2.01 [1.62,2.48]** | **1.54 [1.27,1.87]** | **2.76 [2.17,3.53]** | **0.70 [0.52,0.93]** | **0.77 [0.64,0.93]** | 0.96 [0.71,1.30] | 0.95 [0.72,1.24] | **1.90 [1.55,2.33]** | 1.22 [0.99,1.51] | 0.92 [0.75,1.13] |
| Ethnicity | White – Ref. | - | - | - | - | - | - | - | - | - |  |  |
|  | Ethnic minority | **0.84 [0.71,0.99]** | 1.16 [0.97,1.37] | **1.18 [1.01,1.38]** | **0.27 [0.20,0.37]** | **0.64 [0.49,0.83]** | **0.60 [0.48,0.74]** | 0.85 [0.66,1.09] | **0.81 [0.66,0.98]** | 1.15 [0.97,1.38] | **1.26 [1.07,1.50]** | 1.08 [0.91,1.28] |

Text in bold: indicate 95% CIs that do not include 1.

Table I - Associations between Parental Adverse Childhood Experiences (ACEs) and mental health, general health, and health-risk behaviour outcomes in 8,686 adolescents from the Millennium Cohort Study (estimates are predictive margins of interactions between Parenting ACEs and Sexuality from logistic regression analysis adjusted for sex, ethnicity, and childhood socioeconomic status)

|  | **Predictive margins (95% CI)** | | | | | | | | | | |
| --- | --- | --- | --- | --- | --- | --- | --- | --- | --- | --- | --- |
|  | **Psychological distress** | **SDQ-S** | | | | | **Doctor-diagnosed depression or anxiety** | **Self-harm** | **Suicidality** | **Victimization** | **Self-rated general health** |
|  |  | **Conduct problems** | **Emotional symptoms** | **Hyperactivity/inattention** | **Peer problems** | **Prosocial behaviour difficulty** |  |  |  |  |  |
|  | (1) | (2) | (3) | (4) | (5) | (6) | (7) | (8) | (9) | (10) | (11) |
| 0 # Heterosexual | 0.09 [0.09,0.10] | 0.04 [0.03,0.04] | 0.16 [0.15,0.17] | 0.12 [0.11,0.14] | 0.14 [0.13,0.15] | 0.10 [0.09,0.11] | 0.06 [0.05,0.07] | 0.15 [0.14,0.17] | 0.04 [0.03,0.05] | 0.40 [0.39,0.42] | 0.05 [0.04,0.05] |
| 0 # Bisexual | 0.34 [0.29,0.40] | 0.07 [0.04,0.11] | 0.40 [0.35,0.46] | 0.25 [0.20,0.31] | 0.35 [0.29,0.40] | 0.11 [0.07,0.15] | 0.20 [0.16,0.25] | 0.57 [0.51,0.64] | 0.17 [0.13,0.22] | 0.58 [0.51,0.64] | 0.10 [0.07,0.14] |
| 0 # Gay/Lesb | 0.24 [0.16,0.31] | 0.11 [0.05,0.18] | 0.40 [0.32,0.48] | 0.21 [0.13,0.29] | 0.27 [0.19,0.35] | 0.12 [0.06,0.18] | 0.19 [0.12,0.26] | 0.46 [0.38,0.55] | 0.10 [0.05,0.15] | 0.54 [0.45,0.64] | 0.08 [0.04,0.13] |
| 1 # Heterosexual | 0.14 [0.13,0.16] | 0.06 [0.05,0.07] | 0.21 [0.19,0.23] | 0.13 [0.11,0.15] | 0.20 [0.18,0.22] | 0.10 [0.09,0.12] | 0.10 [0.09,0.12] | 0.20 [0.18,0.22] | 0.06 [0.05,0.08] | 0.46 [0.44,0.49] | 0.07 [0.05,0.08] |
| 1 # Bisexual | 0.38 [0.30,0.45] | 0.13 [0.07,0.18] | 0.33 [0.26,0.40] | 0.26 [0.20,0.33] | 0.31 [0.24,0.38] | 0.12 [0.05,0.19] | 0.27 [0.20,0.34] | 0.59 [0.52,0.66] | 0.23 [0.16,0.29] | 0.63 [0.56,0.71] | 0.11 [0.06,0.16] |
| 1 # Gay/Lesb | 0.37 [0.24,0.50] | 0.07 [0.01,0.14] | 0.44 [0.30,0.57] | 0.29 [0.15,0.42] | 0.40 [0.28,0.53] | 0.10 [0.02,0.18] | 0.28 [0.17,0.39] | 0.56 [0.42,0.69] | 0.20 [0.10,0.31] | 0.64 [0.52,0.76] | 0.20 [0.10,0.30] |
| ≥2 # Heterosexual | 0.16 [0.13,0.18] | 0.05 [0.04,0.07] | 0.21 [0.18,0.24] | 0.15 [0.12,0.18] | 0.22 [0.19,0.25] | 0.10 [0.08,0.12] | 0.14 [0.11,0.16] | 0.25 [0.22,0.29] | 0.09 [0.07,0.11] | 0.49 [0.45,0.52] | 0.08 [0.06,0.10] |
| ≥2 # Bisexual | 0.43 [0.33,0.52] | 0.10 [0.03,0.16] | 0.43 [0.33,0.53] | 0.37 [0.27,0.47] | 0.38 [0.29,0.48] | 0.15 [0.07,0.24] | 0.26 [0.18,0.34] | 0.63 [0.53,0.72] | 0.26 [0.17,0.35] | 0.68 [0.58,0.78] | 0.12 [0.05,0.18] |
| ≥2 # Gay/Lesb | 0.42 [0.20,0.63] | 0.05 [-0.05,0.15] | 0.41 [0.19,0.63] | 0.33 [0.14,0.52] | 0.41 [0.20,0.62] | 0.08 [-0.00,0.16] | 0.24 [0.08,0.41] | 0.47 [0.25,0.70] | 0.13 [0.00,0.27] | 0.77 [0.60,0.93] | 0.20 [0.05,0.36] |

| Continuation of Supplementary Table 9 | | | | | | | | | | | | |
| --- | --- | --- | --- | --- | --- | --- | --- | --- | --- | --- | --- | --- |
|  | Predictive margins (95% CI) | | | | | | | | | | | |
|  | Physical/mental health condition in past year | Poor quality of sleep in past month | Overweight/obese | Self-rated overweight/underweight | Regular smoking habit | Frequent binge drinking | Recreational drug use | Frequent cannabis use | Risky sex | Lack of exercise | Attempting to change weight | Anti-social behaviour |
|  | (12) | (13) | (14) | (15) | (16) | (17) | (18) | (19) | (20) | (21) | (22) | (23) |
| 0 # Heterosexual | 0.13 [0.12,0.14] | 0.27 [0.25,0.28] | 0.25 [0.24,0.27] | 0.41 [0.39,0.43] | 0.08 [0.07,0.09] | 0.15 [0.14,0.16] | 0.28 [0.26,0.29] | 0.14 [0.13,0.15] | 0.35 [0.34,0.37] | 0.20 [0.19,0.22] | 0.58 [0.57,0.60] | 0.25 [0.23,0.26] |
| 0 # Bisexual | 0.29 [0.24,0.35] | 0.42 [0.37,0.47] | 0.32 [0.26,0.38] | 0.53 [0.46,0.59] | 0.18 [0.13,0.22] | 0.16 [0.11,0.21] | 0.38 [0.32,0.44] | 0.18 [0.13,0.24] | 0.50 [0.43,0.56] | 0.26 [0.21,0.32] | 0.62 [0.57,0.68] | 0.30 [0.24,0.36] |
| 0 # Gay/Lesb | 0.28 [0.19,0.37] | 0.32 [0.23,0.41] | 0.36 [0.27,0.45] | 0.59 [0.49,0.69] | 0.09 [0.04,0.13] | 0.16 [0.10,0.23] | 0.30 [0.21,0.39] | 0.11 [0.06,0.17] | 0.40 [0.30,0.50] | 0.32 [0.23,0.41] | 0.63 [0.53,0.72] | 0.22 [0.13,0.30] |
| 1 # Heterosexual | 0.18 [0.16,0.20] | 0.33 [0.31,0.35] | 0.26 [0.24,0.28] | 0.45 [0.42,0.47] | 0.13 [0.11,0.14] | 0.15 [0.14,0.17] | 0.31 [0.28,0.33] | 0.17 [0.15,0.19] | 0.42 [0.39,0.44] | 0.22 [0.20,0.24] | 0.60 [0.57,0.63] | 0.26 [0.24,0.28] |
| 1 # Bisexual | 0.42 [0.34,0.51] | 0.46 [0.39,0.54] | 0.30 [0.23,0.37] | 0.56 [0.48,0.65] | 0.15 [0.10,0.21] | 0.13 [0.08,0.18] | 0.41 [0.33,0.48] | 0.21 [0.14,0.28] | 0.40 [0.32,0.49] | 0.21 [0.15,0.27] | 0.63 [0.55,0.70] | 0.33 [0.26,0.40] |
| 1 # Gay/Lesb | 0.31 [0.20,0.43] | 0.51 [0.37,0.64] | 0.35 [0.22,0.47] | 0.66 [0.54,0.79] | 0.18 [0.09,0.27] | 0.15 [0.05,0.24] | 0.42 [0.28,0.56] | 0.18 [0.08,0.29] | 0.53 [0.41,0.66] | 0.46 [0.33,0.59] | 0.73 [0.61,0.84] | 0.30 [0.18,0.42] |
| ≥2 # Heterosexual | 0.19 [0.16,0.22] | 0.37 [0.34,0.41] | 0.31 [0.28,0.35] | 0.49 [0.45,0.53] | 0.16 [0.14,0.19] | 0.17 [0.14,0.21] | 0.38 [0.34,0.41] | 0.19 [0.16,0.22] | 0.44 [0.40,0.48] | 0.24 [0.21,0.27] | 0.60 [0.57,0.64] | 0.27 [0.24,0.31] |
| ≥2 # Bisexual | 0.39 [0.28,0.49] | 0.59 [0.49,0.69] | 0.29 [0.17,0.40] | 0.62 [0.51,0.73] | 0.23 [0.14,0.31] | 0.23 [0.13,0.32] | 0.55 [0.44,0.66] | 0.27 [0.18,0.35] | 0.51 [0.40,0.62] | 0.37 [0.26,0.48] | 0.73 [0.65,0.82] | 0.39 [0.30,0.49] |
| ≥2 # Gay/Lesb | 0.15 [0.02,0.28] | 0.60 [0.38,0.82] | 0.43 [0.25,0.62] | 0.67 [0.47,0.86] | 0.21 [0.05,0.37] | 0.19 [0.03,0.36] | 0.41 [0.22,0.60] | 0.42 [0.25,0.60] | 0.54 [0.36,0.73] | 0.30 [0.12,0.47] | 0.80 [0.66,0.93] | 0.26 [0.07,0.45] |

Table J – Associations between Parental Adverse Childhood Experiences (ACEs) and mental health in 8,686 adolescents from the Millennium Cohort Study (estimates are from linear regression analysis adjusted for sex, ethnicity, and childhood socioeconomic status)

|  | | **Change in score [β (95% CI)]** | | | | | | | | |
| --- | --- | --- | --- | --- | --- | --- | --- | --- | --- | --- |
|  |  | **(1)** | **(2)** | **(3)** | **(4)** | **(5)** | **(6)** | **(7)** | **(8)** | **(9)** |
|  |  | **Psychological distress**  **(score/24)** | **SDQ-Subscales (score/10)** | | | | | **Mental wellbeing** | **Self-esteem**  **(score/15)** | **BMI** |
|  |  |  | **Emotional symptoms** | **Conduct problems** | **Hyperactivity/inattention** | **Peer problems** | **Prosocial behaviour difficulty** |  |  |  |
| Main effects – Parental ACE score | 0 – Ref. | - | - | - | - | - | - | - | - | - |
|  | 1 | **0.67 [0.39,0.96]** | **0.30 [0.15,0.45]** | **0.19 [0.09,0.30]** | **0.17 [0.01,0.33]** | **0.23 [0.13,0.34]** | -0.03 [-0.14,0.08] | **-0.33 [-0.60,-0.06]** | **-0.33 [-0.54,-0.12]** | 0.16 [-0.13,0.46] |
|  | 2 | **0.91 [0.45,1.38]** | **0.38 [0.15,0.61]** | **0.24 [0.06,0.41]** | 0.21 [-0.02,0.44] | **0.41 [0.24,0.57]** | -0.12 [-0.31,0.07] | **-0.67 [-1.07,-0.28]** | **-0.42 [-0.73,-0.10]** | **0.74 [0.18,1.29]** |
|  | ≥3 | **1.41 [0.64,2.18]** | **0.51 [0.14,0.89]** | **0.27 [0.00,0.53]** | 0.20 [-0.15,0.55] | **0.53 [0.22,0.84]** | -0.05 [-0.32,0.21] | **-0.76 [-1.36,-0.16]** | **-0.52 [-1.01,-0.02]** | 0.76 [-0.17,1.70] |
| Main effects - sexuality | Heterosexual – Ref. | - | - | - | - | - | - | - | - | - |
|  | Bisexual | **4.04 [3.46,4.61]** | **1.68 [1.37,1.99]** | **0.42 [0.21,0.63]** | **0.97 [0.66,1.28]** | **0.98 [0.73,1.22]** | -0.14 [-0.37,0.09] | **-1.84 [-2.32,-1.35]** | **-1.65 [-2.08,-1.22]** | 0.27 [-0.38,0.92] |
|  | Gay/Lesbian | **2.86 [1.96,3.76]** | **1.56 [1.10,2.02]** | **0.45 [0.05,0.85]** | **0.69 [0.19,1.20]** | **0.69 [0.30,1.07]** | -0.20 [-0.54,0.14] | **-1.94 [-2.61,-1.27]** | **-1.31 [-1.94,-0.68]** | **1.33 [0.09,2.57]** |
| Interactions between Parental ACE score and Sexuality | 1 # Hetero – Ref. | - | - | - | - | - | - | - | - | - |
|  | 1 # Bisexual | -0.32 [-1.44,0.80] | **-0.60 [-1.20,-0.01]** | 0.17 [-0.28,0.62] | -0.03 [-0.57,0.51] | -0.25 [-0.69,0.20] | 0.06 [-0.39,0.52] | 0.18 [-0.71,1.07] | 0.49 [-0.25,1.24] | 0.30 [-0.91,1.50] |
|  | 1 # Gay/Lesb | 1.36 [-0.66,3.38] | -0.05 [-1.07,0.98] | -0.22 [-0.96,0.53] | 0.36 [-0.53,1.26] | 0.32 [-0.44,1.08] | 0.01 [-0.67,0.69] | -0.02 [-1.45,1.40] | -0.65 [-2.11,0.81] | -0.81 [-3.08,1.46] |
|  | 2 # Hetero – Ref. | - | - | - | - | - | - | - | - | - |
|  | 2 # Bisexual | 0.58 [-1.23,2.38] | -0.15 [-1.00,0.70] | 0.39 [-0.27,1.04] | 0.39 [-0.49,1.28] | 0.09 [-0.57,0.75] | -0.04 [-0.65,0.57] | -0.04 [-1.29,1.22] | -0.18 [-1.30,0.94] | -0.64 [-2.57,1.29] |
|  | 2 # Gay/Lesb | 2.19 [-0.84,5.22] | 0.19 [-1.50,1.87] | -0.03 [-1.00,0.93] | 0.50 [-0.80,1.81] | 0.53 [-0.58,1.64] | 0.15 [-0.66,0.96] | -0.58 [-2.58,1.42] | -1.30 [-3.42,0.81] | 0.63 [-2.51,3.76] |
|  | ≥3 # Hetero – Ref. | - | - | - | - | - | - | - | - | - |
|  | ≥3 # Bisexual | 0.58 [-1.23,2.38] | -0.15 [-1.00,0.70] | 0.39 [-0.27,1.04] | 0.39 [-0.49,1.28] | 0.09 [-0.57,0.75] | -0.04 [-0.65,0.57] | -0.04 [-1.29,1.22] | -0.18 [-1.30,0.94] | -0.64 [-2.57,1.29] |
|  | ≥3 # Gay/Lesb | 2.19 [-0.84,5.22] | 0.19 [-1.50,1.87] | -0.03 [-1.00,0.93] | 0.50 [-0.80,1.81] | 0.53 [-0.58,1.64] | 0.15 [-0.66,0.96] | -0.58 [-2.58,1.42] | -1.30 [-3.42,0.81] | 0.63 [-2.51,3.76] |
| Sex of study member | Male – Ref. | - | - | - | - | - | - | - | - | - |
|  | Female | **1.72 [1.51,1.94]** | **1.49 [1.36,1.62]** | **-0.33 [-0.40,-0.25]** | **-0.32 [-0.44,-0.20]** | 0.07 [-0.02,0.15] | **0.99 [0.91,1.08]** | **-1.15 [-1.34,-0.95]** | **-0.83 [-0.97,-0.68]** | **0.68 [0.44,0.92]** |
| Childhood socioeconomic class (income class) | IncomeQ3=5 – Ref. | - | - | - | - | - | - | - | - | - |
|  | IncomeQ3=4 | 0.30 [-0.05,0.66] | **0.28 [0.10,0.46]** | 0.04 [-0.07,0.15] | 0.08 [-0.12,0.27] | **0.29 [0.18,0.39]** | -0.06 [-0.20,0.07] | -0.28 [-0.57,0.01] | -0.18 [-0.39,0.03] | **0.48 [0.11,0.85]** |
|  | IncomeQ3=3 | **0.40 [0.07,0.72]** | **0.30 [0.14,0.47]** | 0.05 [-0.05,0.15] | 0.18 [-0.00,0.36] | **0.43 [0.32,0.55]** | -0.12 [-0.25,0.00] | **-0.44 [-0.75,-0.13]** | **-0.61 [-0.85,-0.37]** | **0.78 [0.44,1.13]** |
|  | IncomeQ3=2 | **0.44 [0.08,0.81]** | **0.31 [0.13,0.49]** | **0.29 [0.17,0.41]** | **0.43 [0.24,0.61]** | **0.73 [0.60,0.87]** | **-0.21 [-0.34,-0.07]** | **-0.40 [-0.71,-0.10]** | **-0.35 [-0.58,-0.12]** | **1.17 [0.76,1.58]** |
|  | IncomeQ3=1 | **0.69 [0.28,1.09]** | **0.47 [0.29,0.65]** | **0.34 [0.22,0.46]** | **0.37 [0.17,0.57]** | **0.84 [0.70,0.97]** | **-0.20 [-0.34,-0.06]** | **-0.71 [-1.05,-0.37]** | **-0.61 [-0.85,-0.36]** | **1.21 [0.77,1.65]** |
| Ethnicity | White – Ref. | - | - | - | - | - | - | - | - | - |
|  | Ethnic minority | **-0.69 [-0.99,-0.40]** | **-0.66 [-0.81,-0.51]** | -0.05 [-0.16,0.05] | **-0.58 [-0.74,-0.42]** | **-0.40 [-0.50,-0.30]** | **0.18 [0.07,0.30]** | **0.47 [0.21,0.73]** | **0.53 [0.32,0.74]** | 0.20 [-0.19,0.59] |

Table K - Associations between Parental Adverse Childhood Experiences (ACEs) and mental health outcomes in 8,686 adolescents from the Millennium Cohort Study (estimates are predictive margins of interactions between Parental ACEs and Sexuality from linear regression analysis adjusted for sex, ethnicity, and childhood socioeconomic status)

|  | **Predictive margins [95% CI]** | | | | | | | | |
| --- | --- | --- | --- | --- | --- | --- | --- | --- | --- |
|  | **(1)** | **(2)** | **(3)** | **(4)** | **(5)** | **(6)** | **(7)** | **(8)** | **(9)** |
|  | **Psychological distress**  **(score/24)** | **SDQ-Subscales (score/10)** | | | | | **Mental wellbeing** | **Self-esteem**  **(score/15)** | **BMI** |
|  |  | **Emotional symptoms** | **Conduct problems** | **Hyperactivity/inattention** | **Peer problems** | **Prosocial behaviour difficulty** |  |  |  |
| 0 # Heterosexual | 6.24 [6.10,6.39] | 3.09 [3.01,3.17] | 1.51 [1.46,1.56] | 3.72 [3.64,3.81] | 1.86 [1.80,1.92] | 7.90 [7.85,7.96] | 15.98 [15.85,16.12] | 10.41 [10.29,10.52] | 23.04 [22.89,23.18] |
| 0 # Bisexual | 10.28 [9.72,10.84] | 4.77 [4.46,5.08] | 1.94 [1.73,2.14] | 4.69 [4.40,4.99] | 2.84 [2.61,3.07] | 7.76 [7.54,7.98] | 14.15 [13.69,14.61] | 8.76 [8.34,9.17] | 23.30 [22.66,23.94] |
| 0 # Gay/Lesbian | 9.10 [8.24,9.97] | 4.65 [4.20,5.10] | 1.96 [1.56,2.36] | 4.42 [3.92,4.92] | 2.55 [2.16,2.94] | 7.70 [7.36,8.04] | 14.05 [13.38,14.71] | 9.09 [8.47,9.72] | 24.37 [23.12,25.61] |
| 1 # Heterosexual | 6.92 [6.69,7.15] | 3.39 [3.27,3.51] | 1.71 [1.62,1.79] | 3.90 [3.77,4.03] | 2.10 [2.00,2.19] | 7.87 [7.79,7.96] | 15.65 [15.44,15.86] | 10.08 [9.91,10.25] | 23.20 [22.95,23.44] |
| 1 # Bisexual | 10.63 [9.78,11.49] | **4.47 [4.05,4.89]** | 2.30 [1.95,2.65] | 4.84 [4.45,5.23] | 2.82 [2.49,3.16] | 7.80 [7.45,8.15] | 13.99 [13.28,14.71] | 8.92 [8.36,9.48] | 23.76 [22.79,24.73] |
| 1 # Gay/Lesbian | 11.14 [9.46,12.83] | 4.90 [4.06,5.75] | 1.93 [1.41,2.46] | 4.95 [4.21,5.69] | 3.10 [2.46,3.75] | 7.68 [7.19,8.18] | 13.69 [12.56,14.82] | 8.11 [6.87,9.36] | 23.72 [22.02,25.42] |
| 2 # Heterosexual | 7.16 [6.72,7.60] | 3.47 [3.26,3.69] | 1.75 [1.58,1.91] | 3.93 [3.72,4.15] | 2.27 [2.11,2.43] | 7.78 [7.60,7.97] | 15.31 [14.94,15.68] | 9.99 [9.70,10.28] | 23.77 [23.25,24.29] |
| 2 # Bisexual | 11.77 [10.17,13.37] | 5.00 [4.24,5.77] | 2.56 [2.00,3.11] | 5.30 [4.57,6.03] | 3.34 [2.74,3.93] | 7.60 [7.06,8.15] | 13.44 [12.35,14.52] | 8.16 [7.22,9.11] | 23.40 [21.59,25.20] |
| 2 # Gay/Lesbian | 12.21 [9.29,15.13] | 5.22 [3.55,6.88] | 2.16 [1.29,3.03] | 5.13 [3.92,6.34] | 3.48 [2.42,4.55] | 7.73 [6.99,8.47] | 12.79 [10.92,14.66] | 7.38 [5.34,9.42] | 25.73 [22.94,28.53] |
| ≥3 # Heterosexual | 7.65 [6.87,8.43] | 3.60 [3.23,3.98] | 1.78 [1.52,2.04] | 3.92 [3.58,4.27] | 2.39 [2.09,2.70] | 7.85 [7.59,8.11] | 15.23 [14.63,15.82] | 9.89 [9.39,10.39] | 23.80 [22.86,24.74] |
| ≥3 # Bisexual | 12.66 [10.04,15.28] | 5.47 [4.33,6.61] | 2.22 [1.31,3.13] | 5.47 [4.38,6.55] | 3.48 [2.72,4.24] | 7.21 [6.40,8.03] | 13.12 [11.36,14.88] | 7.84 [6.29,9.40] | 22.59 [20.67,24.52] |
| ≥3 # Gay/Lesbian | 12.46 [8.40,16.51] | 5.04 [3.33,6.74] | 1.79 [0.80,2.78] | 5.97 [3.60,8.34] | 3.51 [2.34,4.68] | 7.38 [6.26,8.50] | 13.56 [10.97,16.15] | 8.02 [4.96,11.07] | 23.45 [20.49,26.42] |

Warwick–Edinburgh Mental Well-being Scale (WEMWBS)

Rosenberg Self-Esteem Scale (5-item)

Table L - Associations between Parenting Adverse Childhood Experiences (ACEs) and mental health, general health, and health-risk behaviours in 8,686 participants from the Millennium Cohort Study (estimates are from logistic regression analysis adjusted for sex, ethnicity, and childhood socioeconomic status)

|  | | **Odds ratio of outcome (95% CI)** | | | | | | | | | | | |
| --- | --- | --- | --- | --- | --- | --- | --- | --- | --- | --- | --- | --- | --- |
|  |  | **(1)** | **(2)** | **(3)** | **(4)** | **(5)** | **(6)** | **(7)** | **(8)** | **(9)** | **(10)** | **(11)** | **(12)** |
|  |  | **Psychological distress** | **SDQ-S** | | | | | **Doctor-diagnosed depression or anxiety** | **Self-harm** | **Suicidality** | **Victimization** | **Poor self-rated general health** | **Physical/mental health condition in past year** |
|  |  |  | **Conduct problems** | **Emotional symptoms** | **Hyperactivity/inattention** | **Peer problems** | **Prosocial behaviour difficulty** |  |  |  |  |  |  |
| **Main effects – Parenting ACE score** | **0 – Ref.** | - | - | - | - | - | - | - | - | - |  |  |  |
|  | **1** | 1.12 [0.92,1.35] | 1.36 [0.99,1.87] | 1.06 [0.90,1.25] | **1.31 [1.10,1.55]** | 1.07 [0.90,1.28] | **1.27 [1.02,1.57]** | 1.06 [0.84,1.34] | 1.12 [0.95,1.31] | 1.12 [0.82,1.51] | **1.15 [1.00,1.31]** | 1.01 [0.77,1.33] | 1.03 [0.86,1.24] |
|  | **2** | 0.99 [0.77,1.27] | 1.26 [0.88,1.79] | 1.00 [0.81,1.25] | **1.62 [1.31,2.01]** | 1.11 [0.88,1.41] | 1.19 [0.93,1.51] | 1.01 [0.73,1.40] | 1.20 [0.96,1.51] | 1.36 [0.94,1.97] | **1.25 [1.08,1.46]** | 1.02 [0.73,1.40] | 1.18 [0.95,1.45] |
| **Main effects - sexuality** | **Heterosexual – Ref.** | - | - | - | - | - | - | - | - | - |  |  |  |
|  | **bisexual** | **3.79 [2.72,5.27]** | 1.43 [0.72,2.83] | **2.90 [2.05,4.11]** | **2.92 [2.08,4.10]** | **2.70 [1.88,3.90]** | 1.02 [0.54,1.92] | **3.09 [2.06,4.62]** | **7.09 [4.98,10.10]** | **4.51 [2.88,7.06]** | **1.99 [1.48,2.69]** | **1.97 [1.20,3.21]** | **2.63 [1.86,3.71]** |
|  | **gay/lesbian** | **3.49 [2.01,6.07]** | 1.79 [0.59,5.45] | **2.93 [1.64,5.23]** | **2.07 [1.06,4.01]** | **2.36 [1.24,4.48]** | 0.66 [0.25,1.75] | **3.33 [1.85,6.00]** | **4.95 [3.05,8.05]** | **4.46 [2.09,9.54]** | **2.67 [1.58,4.53]** | 1.28 [0.51,3.18] | **1.86 [1.02,3.37]** |
| **Interactions between Parenting ACE score and Sexuality** | **1 # Heterosexual** | - | - | - | - | - | - | - | - | - |  |  |  |
|  | **1 # bisexual** | 1.39 [0.88,2.19] | 1.54 [0.64,3.68] | 1.05 [0.64,1.71] | 0.71 [0.45,1.13] | 0.92 [0.56,1.50] | 1.02 [0.43,2.40] | 1.23 [0.71,2.16] | 0.93 [0.57,1.50] | 1.13 [0.63,2.02] | 1.17 [0.76,1.79] | 1.00 [0.50,2.00] | 1.36 [0.86,2.16] |
|  | **1 # gay/lesb** | 0.90 [0.44,1.86] | 1.57 [0.43,5.81] | 1.30 [0.61,2.79] | 1.23 [0.52,2.91] | 1.09 [0.51,2.36] | 2.49 [0.80,7.78] | 1.00 [0.46,2.17] | 0.79 [0.40,1.55] | 0.45 [0.16,1.24] | 0.66 [0.34,1.28] | 2.79 [0.90,8.60] | 1.33 [0.61,2.88] |
|  | **2 # Heterosexual** | - | - | - | - | - | - | - | - | - |  |  |  |
|  | **2 # bisexual** | 1.67 [0.92,3.03] | 2.58 [0.97,6.89] | 1.26 [0.67,2.36] | 1.13 [0.64,1.99] | 1.07 [0.57,2.01] | 2.11 [0.82,5.39] | 1.41 [0.68,2.92] | 0.83 [0.48,1.45] | 1.07 [0.51,2.25] | 0.92 [0.55,1.56] | 1.21 [0.56,2.60] | 0.97 [0.52,1.81] |
|  | **2 # gay/lesb** | 1.14 [0.41,3.16] | 0.43 [0.05,3.40] | 1.32 [0.44,3.98] | 0.80 [0.28,2.27] | 1.12 [0.40,3.12] | 0.50 [0.09,2.59] | 1.03 [0.34,3.07] | 1.07 [0.41,2.76] | 0.56 [0.17,1.92] | 0.62 [0.24,1.63] | 3.36 [0.91,12.50] | 0.79 [0.28,2.28] |
| **Sex of study member** | **Male – Ref.** | - | - | - | - | - | - | - | - | - |  |  |  |
|  | **Female** | **2.28 [1.93,2.70]** | **0.71 [0.56,0.89]** | **3.69 [3.20,4.26]** | 0.86 [0.73,1.01] | **1.13 [1.00,1.27]** | **0.28 [0.23,0.33]** | **2.40 [1.99,2.89]** | **1.63 [1.42,1.87]** | **2.45 [1.92,3.12]** | **0.81 [0.74,0.89]** | **1.24 [1.01,1.54]** | 1.15 [0.99,1.32] |
| **Childhood socioeconomic class (income class)** | **incomeq3=5 – Ref.** | - | - | - | - | - | - | - | - | - |  |  |  |
|  | **incomeq3=4** | **1.28 [1.01,1.63]** | 1.38 [0.93,2.04] | 1.22 [1.00,1.50] | 0.99 [0.79,1.24] | **1.45 [1.18,1.79]** | 1.24 [0.95,1.63] | 1.25 [0.94,1.68] | 1.07 [0.87,1.32] | 1.32 [0.89,1.96] | 0.96 [0.81,1.13] | 1.37 [0.94,1.97] | 1.16 [0.93,1.45] |
|  | **incomeq3=3** | **1.52 [1.17,1.97]** | 1.30 [0.90,1.88] | **1.32 [1.09,1.60]** | 1.13 [0.89,1.43] | **1.89 [1.51,2.35]** | 1.20 [0.91,1.59] | **1.66 [1.23,2.25]** | 1.12 [0.94,1.35] | **1.56 [1.04,2.33]** | 0.97 [0.84,1.11] | **1.57 [1.11,2.23]** | 1.20 [0.96,1.50] |
|  | **incomeq3=2** | **1.71 [1.33,2.20]** | **2.06 [1.47,2.90]** | **1.37 [1.11,1.70]** | **1.28 [1.00,1.64]** | **2.57 [2.05,3.22]** | **1.61 [1.23,2.09]** | **1.63 [1.22,2.19]** | **1.33 [1.08,1.63]** | **2.62 [1.85,3.70]** | 1.08 [0.93,1.26] | **2.00 [1.41,2.82]** | **1.28 [1.01,1.62]** |
|  | **incomeq3=1** | **2.13 [1.67,2.72]** | **2.50 [1.73,3.63]** | **1.46 [1.19,1.79]** | 1.16 [0.90,1.48] | **2.90 [2.31,3.63]** | **1.76 [1.34,2.30]** | **2.26 [1.72,2.97]** | **1.45 [1.15,1.83]** | **3.39 [2.37,4.85]** | 1.14 [0.97,1.33] | **3.39 [2.44,4.71]** | **1.72 [1.38,2.16]** |
| **Ethnicity** | **White – Ref.** | - | - | - | - | - | - | - | - | - |  |  |  |
|  | **Ethnic minority** | **0.70 [0.57,0.85]** | 0.80 [0.58,1.11] | **0.54 [0.44,0.67]** | **0.54 [0.42,0.70]** | **0.62 [0.52,0.73]** | 0.86 [0.70,1.07] | **0.42 [0.31,0.57]** | **0.63 [0.51,0.79]** | **0.57 [0.42,0.77]** | **0.60 [0.52,0.70]** | 1.01 [0.77,1.33] | **0.70 [0.56,0.87]** |

| Continuation of Supplementary Table 12 | | | | | | | | | | | | |
| --- | --- | --- | --- | --- | --- | --- | --- | --- | --- | --- | --- | --- |
|  | | **Odds ratio of outcome (95% CI)** | | | | | | | | | | |
|  |  | **(13)** | **(14)** | **(15)** | **(16)** | **(17)** | **(18)** | **(19)** | **(20)** | **(21)** | **(22)** | **(23)** |
|  |  | **Poor quality of sleep in past month** | **Overweight/obese** | **Self-rated overweight/underweight** | **Regular smoking habit** | **Frequent binge drinking** | **Recreational drug use** | **Frequent cannabis use** | **Risky sex** | **Lack of exercise** | **Attempting to change weight** | **Anti-social behaviour** |
| **Main effects – Parenting ACE score** | **0 – Ref.** | - | - | - | - | - | - | - | - | - |  |  |
|  | **1** | 1.07 [0.92,1.24] | 1.05 [0.90,1.23] | 1.07 [0.92,1.25] | 1.28 [1.04,1.57] | 1.07 [0.88,1.29] | **1.17 [1.02,1.35]** | 1.10 [0.87,1.40] | 1.11 [0.95,1.30] | 0.98 [0.84,1.14] | 1.05 [0.91,1.20] | **1.33 [1.15,1.54]** |
|  | **2** | 0.95 [0.78,1.16] | **1.24 [1.03,1.48]** | 1.14 [0.95,1.35] | **1.40 [1.10,1.78]** | 1.14 [0.91,1.44] | 1.16 [0.96,1.39] | **1.34 [1.01,1.77]** | 1.18 [0.95,1.48] | 1.02 [0.83,1.25] | 1.19 [0.98,1.44] | **1.50 [1.27,1.78]** |
| **Main effects - sexuality** | **Heterosexual – Ref.** | - | - | - | - | - | - | - | - | - | - | **-** |
|  | **bisexual** | **2.25 [1.61,3.15]** | 1.28 [0.89,1.84] | 1.41 [1.02,1.95] | **1.79 [1.17,2.76]** | 1.14 [0.71,1.84] | **1.49 [1.08,2.03]** | 1.34 [0.85,2.12] | **1.46 [1.04,2.05]** | 1.15 [0.79,1.67] | 1.19 [0.87,1.64] | **1.57 [1.10,2.24]** |
|  | **gay/lesb** | 1.50 [0.88,2.55] | 2.02 [1.25,3.26] | **2.89 [1.70,4.92]** | 0.87 [0.39,1.93] | 0.81 [0.31,2.08] | 1.33 [0.76,2.30] | 0.98 [0.37,2.64] | 1.88 [1.02,3.47] | 1.56 [0.92,2.66] | 1.71 [0.96,3.05] | 1.44 [0.82,2.52] |
| **Interactions between Parenting ACE score and Sexuality** | **1 # Heterosexual** | - | - | - | - | - | - | - | - | - |  |  |
|  | **1 # bisexual** | 0.82 [0.50,1.35] | 1.02 [0.62,1.67] | 1.36 [0.84,2.20] | 1.03 [0.57,1.87] | 0.94 [0.47,1.88] | 1.37 [0.87,2.15] | 1.22 [0.67,2.25] | 0.93 [0.57,1.52] | 1.14 [0.67,1.92] | 1.06 [0.67,1.69] | 0.91 [0.55,1.53] |
|  | **1 # gay/lesb** | 1.08 [0.50,2.31] | 0.69 [0.33,1.42] | 0.64 [0.31,1.33] | 1.89 [0.70,5.07] | 1.60 [0.45,5.65] | 0.83 [0.39,1.76] | 1.12 [0.33,3.86] | 0.65 [0.28,1.52] | 1.43 [0.69,2.96] | 0.75 [0.34,1.65] | 0.63 [0.30,1.34] |
|  | **2 # Heterosexual** | - | - | - | - | - | - | - | - | - |  |  |
|  | **2 # bisexual** | 0.99 [0.52,1.91] | 0.70 [0.39,1.25] | 1.18 [0.65,2.16] | 1.20 [0.60,2.41] | 1.34 [0.61,2.94] | 1.06 [0.57,1.94] | 1.07 [0.51,2.25] | 0.90 [0.49,1.66] | 1.91 [1.01,3.63] | 0.83 [0.45,1.55] | 0.88 [0.48,1.63] |
|  | **2 # gay/lesb** | 1.45 [0.52,4.06] | 0.94 [0.40,2.22] | 0.76 [0.28,2.03] | 1.18 [0.35,3.94] | 0.67 [0.12,3.62] | 1.25 [0.48,3.23] | 0.81 [0.17,3.92] | 0.96 [0.29,3.20] | 2.09 [0.79,5.57] | 0.97 [0.33,2.84] | 0.36 [0.11,1.13] |
| **Sex of study member** | **Male – Ref.** | - | - | - | - | - | - | - | - | - | **-** | **-** |
|  | **Female** | **1.21 [1.06,1.37]** | **1.30 [1.15,1.48]** | **1.31 [1.15,1.49]** | 0.96 [0.81,1.13] | 0.91 [0.77,1.07] | **0.73 [0.64,0.82]** | **0.57 [0.47,0.70]** | 0.89 [0.76,1.03] | **1.94 [1.73,2.18]** | **1.57 [1.39,1.77]** | **0.34 [0.30,0.38]** |
| **Childhood socioeconomic class (income class)** | **incomeq3=5 – Ref.** | - | - | - | - | - | - | - | - | - | - | - |
|  | **incomeq3=4** | 1.12 [0.92,1.36] | **1.25 [1.01,1.55]** | 1.02 [0.85,1.23] | 1.06 [0.82,1.37] | 0.99 [0.78,1.26] | 0.89 [0.76,1.05] | 1.04 [0.80,1.35] | 1.05 [0.85,1.30] | 1.20 [0.98,1.46] | 0.93 [0.78,1.10] | 0.86 [0.72,1.04] |
|  | **incomeq3=3** | **1.25 [1.02,1.53]** | **1.53 [1.27,1.84]** | **1.23 [1.04,1.45]** | **1.42 [1.08,1.87]** | 0.96 [0.77,1.20] | 0.88 [0.73,1.05] | 0.98 [0.73,1.32] | 1.01 [0.82,1.24] | **1.48 [1.20,1.83]** | 1.13 [0.95,1.34] | 0.87 [0.73,1.04] |
|  | **incomeq3=2** | **1.37 [1.12,1.67]** | **1.93 [1.60,2.32]** | **1.42 [1.19,1.70]** | **2.39 [1.85,3.09]** | **0.70 [0.54,0.90]** | 0.92 [0.76,1.13] | 1.14 [0.84,1.54] | 1.00 [0.79,1.26] | **1.94 [1.59,2.37]** | 1.10 [0.91,1.32] | 1.00 [0.82,1.20] |
|  | **incomeq3=1** | **1.50 [1.20,1.87]** | **2.08 [1.69,2.56]** | **1.64 [1.35,1.98]** | **3.30 [2.61,4.17]** | **0.72 [0.55,0.95]** | 0.87 [0.72,1.05] | 1.05 [0.78,1.42] | 1.06 [0.82,1.37] | **1.99 [1.63,2.43]** | **1.25 [1.01,1.53]** | 0.97 [0.79,1.19] |
| **Ethnicity** | **White – Ref.** | - | - | - | - | - | - | - | - | - |  |  |
|  | **Ethnic minority** | **0.85 [0.72,1.00]** | 1.15 [0.97,1.36] | **1.18 [1.01,1.38]** | **0.28 [0.20,0.37]** | **0.64 [0.49,0.83]** | **0.60 [0.49,0.74]** | 0.84 [0.66,1.09] | **0.81 [0.67,0.98]** | 1.16 [0.97,1.38] | **1.26 [1.06,1.49]** | 1.07 [0.90,1.27] |

Text in bold: indicate 95% CIs that do not include 1.

Table M - Associations between Parenting Adverse Childhood Experiences (ACEs) and mental health, general health, and health-risk behaviour outcomes in 8,686 participants from the Millennium Cohort Study (estimates are predictive margins of interactions between Parenting ACEs and Sexuality from logistic regression analysis adjusted for sex, ethnicity, and childhood socioeconomic status)

|  | **Predictive margins (95% CI)** | | | | | | | | | | |
| --- | --- | --- | --- | --- | --- | --- | --- | --- | --- | --- | --- |
|  | **Psychological distress** | **SDQ-S** | | | | | **Doctor-diagnosed depression or anxiety** | **Self-harm** | **Suicidality** | **Victimization** | **Self-rated general health** |
|  |  | **Conduct problems** | **Emotional symptoms** | **Hyperactivity/inattention** | **Peer problems** | **Prosocial behaviour difficulty** |  |  |  |  |  |
|  | (1) | (2) | (3) | (4) | (5) | (6) | (7) | (8) | (9) | (10) | (11) |
| 0 # Heterosexual | 0.11 [0.10,0.12] | 0.04 [0.03,0.05] | 0.18 [0.16,0.19] | 0.11 [0.10,0.12] | 0.16 [0.14,0.18] | 0.09 [0.08,0.10] | 0.08 [0.07,0.09] | 0.17 [0.15,0.18] | 0.05 [0.04,0.06] | 0.41 [0.38,0.43] | 0.06 [0.05,0.07] |
| 0 # Bisexual | 0.31 [0.25,0.37] | 0.06 [0.03,0.09] | 0.35 [0.29,0.42] | 0.26 [0.20,0.32] | 0.33 [0.26,0.40] | 0.10 [0.05,0.15] | 0.21 [0.15,0.26] | 0.57 [0.50,0.64] | 0.19 [0.14,0.24] | 0.57 [0.50,0.63] | 0.10 [0.06,0.14] |
| 0 # Gay/Lesb | 0.29 [0.19,0.40] | 0.06 [0.00,0.13] | 0.35 [0.24,0.47] | 0.21 [0.11,0.30] | 0.30 [0.18,0.42] | 0.06 [0.01,0.11] | 0.22 [0.13,0.32] | 0.50 [0.38,0.61] | 0.18 [0.08,0.27] | 0.63 [0.52,0.74] | 0.07 [0.02,0.13] |
| 1 # Heterosexual | 0.12 [0.11,0.13] | 0.05 [0.04,0.06] | 0.19 [0.17,0.20] | 0.13 [0.12,0.15] | 0.17 [0.16,0.19] | 0.11 [0.10,0.12] | 0.09 [0.08,0.10] | 0.18 [0.17,0.20] | 0.05 [0.04,0.06] | 0.44 [0.42,0.46] | 0.06 [0.05,0.06] |
| 1 # Bisexual | 0.39 [0.34,0.45] | 0.09 [0.05,0.13] | 0.40 [0.35,0.46] | 0.23 [0.18,0.29] | 0.34 [0.28,0.40] | 0.11 [0.06,0.15] | 0.25 [0.19,0.30] | 0.60 [0.53,0.67] | 0.20 [0.15,0.26] | 0.64 [0.58,0.71] | 0.10 [0.06,0.14] |
| 1 # Gay/Lesb | 0.29 [0.20,0.37] | 0.13 [0.06,0.19] | 0.45 [0.35,0.54] | 0.27 [0.18,0.37] | 0.34 [0.24,0.44] | 0.17 [0.10,0.24] | 0.22 [0.14,0.30] | 0.47 [0.37,0.56] | 0.10 [0.04,0.15] | 0.59 [0.49,0.69] | 0.17 [0.09,0.24] |
| 2 # Heterosexual | 0.11 [0.09,0.13] | 0.04 [0.03,0.06] | 0.18 [0.16,0.20] | 0.16 [0.14,0.18] | 0.17 [0.15,0.20] | 0.10 [0.09,0.12] | 0.08 [0.06,0.10] | 0.20 [0.18,0.23] | 0.06 [0.05,0.08] | 0.46 [0.43,0.49] | 0.06 [0.04,0.07] |
| 2 # Bisexual | 0.45 [0.35,0.55] | 0.17 [0.09,0.26] | 0.42 [0.32,0.53] | 0.42 [0.32,0.52] | 0.39 [0.29,0.48] | 0.20 [0.11,0.29] | 0.28 [0.18,0.38] | 0.59 [0.49,0.70] | 0.25 [0.16,0.34] | 0.64 [0.54,0.73] | 0.14 [0.07,0.21] |
| 2 # Gay/Lesb | 0.34 [0.18,0.50] | 0.07 [-0.01,0.15] | 0.45 [0.29,0.61] | 0.27 [0.11,0.43] | 0.37 [0.21,0.53] | 0.04 [0.00,0.07] | 0.23 [0.09,0.37] | 0.54 [0.37,0.72] | 0.14 [0.03,0.24] | 0.59 [0.42,0.75] | 0.19 [0.06,0.33] |

| Continuation of Supplementary Table 13 | | | | | | | | | | | | |
| --- | --- | --- | --- | --- | --- | --- | --- | --- | --- | --- | --- | --- |
|  | Predictive margins (95% CI) | | | | | | | | | | | |
|  | Physical/mental health condition in past year | Poor quality of sleep in past month | Overweight/obese | Self-rated overweight/underweight | Regular smoking habit | Frequent binge drinking | Recreational drug use | Frequent cannabis use | Risky sex | Lack of exercise | Attempting to change weight | Anti-social behaviour |
|  | (12) | (13) | (14) | (15) | (16) | (17) | (18) | (19) | (20) | (21) | (22) | (23) |
| 0 # Heterosexual | 0.14 [0.13,0.16] | 0.29 [0.27,0.30] | 0.25 [0.23,0.27] | 0.41 [0.40,0.43] | 0.09 [0.08,0.10] | 0.15 [0.13,0.16] | 0.27 [0.25,0.30] | 0.15 [0.13,0.16] | 0.36 [0.33,0.38] | 0.22 [0.20,0.24] | 0.57 [0.54,0.59] | 0.22 [0.20,0.23] |
| 0 # Bisexual | 0.31 [0.25,0.38] | 0.45 [0.38,0.52] | 0.31 [0.24,0.38] | 0.49 [0.42,0.56] | 0.16 [0.11,0.21] | 0.16 [0.11,0.21] | 0.37 [0.30,0.45] | 0.20 [0.14,0.26] | 0.48 [0.41,0.55] | 0.22 [0.16,0.28] | 0.61 [0.55,0.67] | 0.31 [0.24,0.38] |
| 0 # Gay/Lesb | 0.24 [0.13,0.34] | 0.38 [0.27,0.49] | 0.41 [0.30,0.51] | 0.64 [0.54,0.75] | 0.10 [0.04,0.15] | 0.15 [0.06,0.23] | 0.36 [0.24,0.48] | 0.17 [0.08,0.26] | 0.43 [0.31,0.56] | 0.31 [0.21,0.41] | 0.67 [0.57,0.78] | 0.28 [0.18,0.38] |
| 1 # Heterosexual | 0.15 [0.13,0.16] | 0.31 [0.29,0.33] | 0.26 [0.25,0.28] | 0.44 [0.42,0.46] | 0.11 [0.10,0.13] | 0.15 [0.14,0.17] | 0.31 [0.29,0.33] | 0.16 [0.14,0.17] | 0.40 [0.38,0.42] | 0.21 [0.19,0.22] | 0.59 [0.57,0.61] | 0.27 [0.25,0.29] |
| 1 # Bisexual | 0.38 [0.31,0.45] | 0.47 [0.41,0.54] | 0.32 [0.25,0.39] | 0.59 [0.52,0.67] | 0.18 [0.13,0.23] | 0.14 [0.09,0.19] | 0.46 [0.39,0.53] | 0.21 [0.15,0.27] | 0.44 [0.37,0.50] | 0.27 [0.20,0.33] | 0.65 [0.59,0.72] | 0.33 [0.26,0.39] |
| 1 # Gay/Lesb | 0.31 [0.22,0.41] | 0.45 [0.34,0.56] | 0.32 [0.22,0.41] | 0.60 [0.49,0.71] | 0.17 [0.09,0.24] | 0.21 [0.12,0.29] | 0.30 [0.21,0.40] | 0.20 [0.11,0.29] | 0.41 [0.30,0.52] | 0.35 [0.25,0.46] | 0.66 [0.56,0.76] | 0.26 [0.16,0.35] |
| 2 # Heterosexual | 0.17 [0.15,0.19] | 0.29 [0.26,0.31] | 0.29 [0.26,0.32] | 0.44 [0.41,0.47] | 0.12 [0.10,0.14] | 0.16 [0.14,0.19] | 0.31 [0.27,0.34] | 0.16 [0.14,0.18] | 0.38 [0.35,0.41] | 0.22 [0.19,0.24] | 0.63 [0.60,0.66] | 0.29 [0.26,0.31] |
| 2 # Bisexual | 0.37 [0.27,0.47] | 0.48 [0.38,0.58] | 0.28 [0.19,0.36] | 0.63 [0.52,0.73] | 0.22 [0.13,0.31] | 0.22 [0.13,0.31] | 0.41 [0.31,0.52] | 0.21 [0.12,0.30] | 0.54 [0.43,0.66] | 0.39 [0.29,0.49] | 0.70 [0.60,0.79] | 0.36 [0.27,0.45] |
| 2 # Gay/Lesb | 0.24 [0.10,0.37] | 0.42 [0.26,0.58] | 0.42 [0.25,0.59] | 0.63 [0.46,0.81] | 0.11 [0.02,0.20] | 0.08 [-0.01,0.17] | 0.44 [0.25,0.63] | 0.16 [0.03,0.28] | 0.61 [0.42,0.81] | 0.46 [0.28,0.65] | 0.73 [0.60,0.87] | 0.16 [0.03,0.29] |

Table N - Associations between Parenting Adverse Childhood Experiences (ACEs) and mental health in 8,686 participants from the Millennium Cohort Study (estimates are from linear regression analysis adjusted for sex, ethnicity, and childhood socioeconomic status)

|  | | Change in score [β (95% CI)] | | | | | | | | |
| --- | --- | --- | --- | --- | --- | --- | --- | --- | --- | --- |
|  |  | (1) | (2) | (3) | (4) | (5) | (6) | (7) | (8) | (9) |
|  |  | Psychological distress  (score/24) | SDQ-Subscales (score/10) | | | | | Mental wellbeing | Self-esteem  (score/15) | BMI |
|  |  |  | Emotional symptoms | Conduct problems | Hyperactivity/inattention | Peer problems | Prosocial behaviour difficulty |  |  |  |
| Main effects – Parenting ACE score | 0 – Ref. | - | - | - | - | - | - | - | - | - |
|  | 1 | 0.17 [-0.12,0.45] | 0.03 [-0.11,0.17] | **0.15^***^ [0.06,0.24]** | **0.30^***^ [0.16,0.44]** | 0.05 [-0.05,0.15] | **-0.16^**^ [-0.26,-0.05]** | **-0.35^**^ [-0.61,-0.10]** | -0.15 [-0.33,0.04] | 0.19 [-0.09,0.47] |
|  | 2 | 0.16 [-0.17,0.49] | -0.01 [-0.19,0.16] | **0.31^***^ [0.20,0.43]** | **0.55^***^ [0.37,0.72]** | **0.15^*^ [0.01,0.28]** | **-0.20^**^ [-0.32,-0.07]** | **-0.42^**^ [-0.71,-0.13]** | -0.20 [-0.44,0.04] | **0.62^***^ [0.27,0.98]** |
| Main effects - sexuality | Heterosexual – Ref. | - | - | - | - | - | - | - | - | - |
|  | Bisexual | **3.54^***^ [2.88,4.19]** | **1.33^***^ [0.97,1.70]** | **0.26^*^ [0.05,0.47]** | **0.89^***^ [0.55,1.22]** | **0.88^***^ [0.60,1.16]** | -0.18 [-0.42,0.06] | **-1.69^***^ [-2.22,-1.16]** | **-1.39^***^ [-1.84,-0.94]** | 0.63 [-0.07,1.34] |
|  | Gay/Lesbian | **3.38^***^ [2.04,4.72]** | **1.30^***^ [0.57,2.03]** | 0.20 [-0.19,0.59] | **0.93^**^ [0.35,1.50]** | **0.65^*^ [0.11,1.18]** | 0.09 [-0.24,0.43] | **-2.03^***^ [-2.93,-1.13]** | **-1.62^***^ [-2.56,-0.69]** | 1.43 [-0.00,2.87] |
| Interactions between Parenting ACE score and Sexuality | 1 # Hetero – Ref. | - | - | - | - | - | - | - | - | - |
|  | 1 # Bisexual | **1.00^*^ [0.02,1.97]** | 0.23 [-0.32,0.78] | **0.38^*^ [0.04,0.72]** | 0.14 [-0.35,0.62] | 0.07 [-0.33,0.46] | 0.14 [-0.24,0.52] | -0.20 [-0.90,0.50] | -0.34 [-1.00,0.32] | -0.52 [-1.55,0.51] |
|  | 1 # Gay/Lesb | 0.03 [-1.69,1.76] | 0.34 [-0.55,1.23] | 0.33 [-0.25,0.91] | -0.07 [-0.90,0.77] | 0.23 [-0.40,0.86] | -0.48 [-1.05,0.09] | 0.04 [-1.30,1.39] | 0.10 [-1.22,1.41] | -0.87 [-2.90,1.16] |
|  | 2 # Hetero – Ref. | - | - | - | - | - | - | - | - | - |
|  | 2 # Bisexual | 1.21 [-0.15,2.57] | 0.61 [-0.03,1.24] | **0.75^**^ [0.19,1.31]** | **0.74^*^ [0.08,1.39]** | 0.26 [-0.26,0.78] | -0.24 [-0.77,0.29] | -0.55 [-1.65,0.55] | -0.33 [-1.24,0.58] | -1.08 [-2.35,0.20] |
|  | 2 # Gay/Lesb | 1.12 [-1.37,3.60] | 0.66 [-0.52,1.83] | 0.08 [-0.64,0.80] | 0.03 [-1.02,1.08] | 0.62 [-0.31,1.54] | -0.37 [-1.00,0.25] | -0.03 [-1.45,1.39] | -0.53 [-1.95,0.89] | 0.54 [-2.03,3.12] |
| Sex of study member | Male – Ref. | - | - | - | - | - | - | - | - | - |
|  | Female | **1.74^***^ [1.52,1.95]** | **1.49^***^ [1.37,1.62]** | **-0.30^***^ [-0.38,-0.22]** | **-0.28^***^ [-0.40,-0.16]** | 0.07 [-0.01,0.16] | **0.98^***^ [0.89,1.06]** | **-1.18^***^ [-1.37,-0.99]** | **-0.84^***^ [-0.99,-0.69]** | **0.71^***^ [0.46,0.95]** |
| Childhood socioeconomic class (income class) | IncomeQ3=5 – Ref. | - | - | - | - | - | - | - | - | - |
|  | IncomeQ3=4 | 0.32 [-0.04,0.67] | **0.28^**^ [0.11,0.46]** | 0.04 [-0.07,0.15] | 0.07 [-0.12,0.26] | **0.29^***^ [0.19,0.40]** | -0.06 [-0.20,0.07] | -0.28 [-0.57,0.01] | -0.18 [-0.39,0.02] | **0.48^*^ [0.10,0.85]** |
|  | IncomeQ3=3 | **0.49^**^ [0.16,0.83]** | **0.33^***^ [0.17,0.50]** | 0.07 [-0.04,0.17] | **0.20^*^ [0.01,0.38]** | **0.47^***^ [0.35,0.58]** | **-0.13^*^ [-0.25,-0.00]** | **-0.48^**^ [-0.79,-0.17]** | **-0.65^***^ [-0.90,-0.41]** | **0.81^***^ [0.46,1.16]** |
|  | IncomeQ3=2 | **0.64^***^ [0.27,1.01]** | **0.38^***^ [0.20,0.56]** | **0.32^***^ [0.20,0.45]** | **0.46^***^ [0.27,0.64]** | **0.80^***^ [0.66,0.93]** | **-0.21^**^ [-0.35,-0.08]** | **-0.50^**^ [-0.80,-0.20]** | **-0.43^***^ [-0.66,-0.20]** | **1.25^***^ [0.84,1.65]** |
|  | IncomeQ3=1 | **0.96^***^ [0.56,1.36]** | **0.56^***^ [0.38,0.74]** | **0.39^***^ [0.27,0.51]** | **0.42^***^ [0.23,0.62]** | **0.93^***^ [0.80,1.07]** | **-0.22^**^ [-0.36,-0.08]** | **-0.86^***^ [-1.20,-0.53]** | **-0.72^***^ [-0.97,-0.48]** | **1.32^***^ [0.89,1.76]** |
| Ethnicity | White – Ref. | - | - | - | - | - | - | - | - | - |
|  | Ethnic minority | **-0.69^***^ [-0.98,-0.39]** | **-0.65^***^ [-0.80,-0.50]** | -0.06 [-0.16,0.05] | **-0.59^***^ [-0.75,-0.44]** | **-0.39^***^ [-0.49,-0.29]** | **0.18^**^ [0.07,0.30]** | **0.47^***^ [0.21,0.73]** | **0.53^***^ [0.32,0.74]** | 0.19 [-0.21,0.58] |

Warwick–Edinburgh Mental Well-being Scale (WEMWBS)

Rosenberg Self-Esteem Scale (5-item) Text in bold: indicate 95% CIs that do not include 0.

Table O - Associations between Parenting Adverse Childhood Experiences (ACEs) and mental health outcomes in 8,686 participants from the Millennium Cohort Study (estimates are predictive margins of interactions between Parenting ACEs and Sexuality from linear regression analysis adjusted for sex, ethnicity, and childhood socioeconomic status)

|  | **Predictive margins [95% CI]** | | | | | | | | |
| --- | --- | --- | --- | --- | --- | --- | --- | --- | --- |
|  | **(1)** | **(2)** | **(3)** | **(4)** | **(5)** | **(6)** | **(7)** | **(8)** | **(9)** |
|  | **Psychological distress**  **(score/24)** | **SDQ-Subscales (score/10)** | | | | | **Mental wellbeing** | **Self-esteem**  **(score/15)** | **BMI** |
|  |  | **Emotional symptoms** | **Conduct problems** | **Hyperactivity/inattention** | **Peer problems** | **Prosocial behaviour difficulty** |  |  |  |
| 0 # Heterosexual | 6.46 [6.26,6.65] | 3.21 [3.11,3.31] | 1.47 [1.41,1.54] | 3.57 [3.46,3.67] | 1.93 [1.85,2.01] | 7.99 [7.91,8.06] | 16.04 [15.87,16.21] | 10.36 [10.23,10.50] | 22.98 [22.80,23.16] |
| 0 # Bisexual | 9.99 [9.35,10.63] | 4.55 [4.20,4.89] | 1.73 [1.54,1.93] | 4.45 [4.15,4.76] | 2.81 [2.55,3.08] | 7.80 [7.57,8.04] | 14.35 [13.84,14.86] | 8.97 [8.54,9.40] | 23.61 [22.92,24.30] |
| 0 # Gay/Lesbian | 9.83 [8.49,11.17] | 4.51 [3.78,5.24] | 1.68 [1.29,2.06] | 4.50 [3.94,5.05] | 2.58 [2.05,3.12] | 8.08 [7.75,8.41] | 14.01 [13.12,14.90] | 8.74 [7.81,9.66] | 24.41 [23.01,25.81] |
| 1 # Heterosexual | 6.62 [6.45,6.80] | 3.24 [3.15,3.34] | 1.62 [1.56,1.68] | 3.87 [3.78,3.96] | 1.98 [1.91,2.05] | 7.83 [7.76,7.90] | 15.69 [15.52,15.85] | 10.21 [10.08,10.35] | 23.17 [22.98,23.35] |
| 1 # Bisexual | 11.16 [10.53,11.79] | 4.81 [4.47,5.15] | 2.26 [2.03,2.49] | 4.89 [4.56,5.22] | 2.93 [2.68,3.19] | 7.79 [7.51,8.06] | 13.80 [13.33,14.27] | 8.48 [8.04,8.93] | 23.28 [22.56,24.00] |
| 1 # Gay/Lesbian | 10.03 [8.97,11.10] | 4.89 [4.39,5.38] | 2.16 [1.74,2.57] | 4.73 [4.14,5.32] | 2.86 [2.46,3.27] | 7.44 [7.04,7.85] | 13.70 [12.87,14.53] | 8.69 [7.86,9.51] | 23.73 [22.54,24.93] |
| 2 # Heterosexual | 6.62 [6.35,6.89] | 3.20 [3.05,3.35] | 1.79 [1.69,1.88] | 4.11 [3.96,4.27] | 2.08 [1.97,2.19] | 7.79 [7.68,7.90] | 15.62 [15.37,15.87] | 10.16 [9.96,10.36] | 23.60 [23.30,23.90] |
| 2 # Bisexual | 11.37 [10.21,12.53] | 5.14 [4.63,5.65] | 2.80 [2.30,3.29] | 5.73 [5.23,6.24] | 3.22 [2.81,3.63] | 7.37 [6.92,7.82] | 13.38 [12.47,14.29] | 8.44 [7.74,9.14] | 23.16 [22.12,24.20] |
| 2 # Gay/Lesbian | 11.11 [9.21,13.01] | 5.16 [4.30,6.01] | 2.07 [1.45,2.68] | 5.07 [4.15,6.00] | 3.34 [2.63,4.06] | 7.51 [6.99,8.03] | 13.56 [12.57,14.55] | 8.01 [7.00,9.02] | 25.58 [23.26,27.91] |

Warwick–Edinburgh Mental Well-being Scale (WEMWBS)

Rosenberg Self-Esteem Scale (5-item)

Table P - Associations between experiencee of bullying (ACEs) and mental health, general health, and health-risk Behaviours in 8,686 participants from the Millennium Cohort Study (estimates are from logistic regression analysis adjusted for sex, ethnicity, and childhood socioeconomic status)

|  | | **Odds ratio of outcome (95% CI)** | | | | | | | | | | | |
| --- | --- | --- | --- | --- | --- | --- | --- | --- | --- | --- | --- | --- | --- |
|  |  | (1) | **(2)** | **(3)** | **(4)** | **(5)** | **(6)** | **(7)** | **(8)** | **(9)** | **(10)** | **(11)** | **(12)** |
|  |  | **Psychological distress** | **SDQ-S** | | | | | **Doctor-diagnosed depression or anxiety** | **Self-harm** | **Suicidality** | **Victimization** | **Poor self-rated general health** | **Physical/mental health condition in past year** |
|  |  |  | **Conduct problems** | **Emotional symptoms** | **Hyperactivity/inattention** | **Peer problems** | **Prosocial behaviour difficulty** |  |  |  |  |  |  |
| Main effects – Bullying | Absent – Ref. | - | - | - | - | - | - | - | - | - |  |  |  |
|  | Present | **1.88 [1.54,2.29]** | **2.04 [1.56,2.67]** | **1.79 [1.49,2.14]** | **1.81 [1.51,2.16]** | **2.76 [2.35,3.24]** | **1.23 [1.01,1.51]** | **1.98 [1.61,2.44]** | **2.12 [1.81,2.49]** | **2.89 [2.26,3.69]** | **2.31 [2.01,2.66]** | **1.99 [1.52,2.61]** | **1.71 [1.43,2.06]** |
| Main effects - sexuality | Heterosexual – Ref. | - | - | - | - | - | - | - | - | - |  |  |  |
|  | bisexual | **4.70 [3.71,5.95]** | **1.72 [1.04,2.83]** | **2.87 [2.25,3.67]** | **2.41 [1.84,3.15]** | **2.36 [1.83,3.05]** | 1.34 [0.91,1.98] | **3.89 [2.96,5.11]** | **7.07 [5.41,9.25]** | **5.37 [3.70,7.80]** | **2.08 [1.64,2.64]** | **1.75 [1.19,2.58]** | **2.69 [2.06,3.50]** |
|  | gay/lesbian | **2.08 [1.26,3.41]** | 1.58 [0.62,4.04] | **2.44 [1.62,3.66]** | 1.42 [0.88,2.31] | **2.13 [1.37,3.31]** | 0.65 [0.31,1.38] | **3.13 [1.96,5.02]** | **3.44 [2.29,5.17]** | **2.88 [1.58,5.26]** | 1.34 [0.93,1.95] | **2.42 [1.33,4.41]** | **1.63 [1.05,2.52]** |
| Interactions between Ex-Bullying and Sexuality | 1 # Heterosexual | - | - | - | - | - | - | - | - | - |  |  |  |
|  | 1 # bisexual | 0.83 [0.53,1.30] | 1.30 [0.65,2.61] | 0.98 [0.61,1.59] | 0.98 [0.63,1.52] | 0.98 [0.61,1.56] | 0.74 [0.38,1.42] | 0.64 [0.38,1.07] | 0.66 [0.43,1.02] | **0.57 [0.34,0.97]** | 0.74 [0.47,1.17] | 1.11 [0.62,2.00] | 1.10 [0.70,1.72] |
|  | 1 # gay/lesb | **2.13 [1.01,4.52]** | 1.29 [0.38,4.41] | 1.77 [0.86,3.63] | 1.87 [0.90,3.87] | 0.95 [0.50,1.83] | 2.33 [0.88,6.19] | 0.87 [0.39,1.95] | 1.42 [0.73,2.79] | 0.68 [0.31,1.52] | **2.21 [1.09,4.50]** | 1.00 [0.41,2.46] | 1.28 [0.60,2.77] |
| Sex of study member | Male – Ref. | - | - | - | - | - | - | - | - | - |  |  |  |
|  | Female | **2.37 [2.00,2.80]** | **0.71 [0.56,0.90]** | **3.86 [3.35,4.46]** | 0.86 [0.73,1.01] | **1.19 [1.06,1.34]** | **0.27 [0.23,0.32]** | **2.49 [2.08,2.98]** | **1.70 [1.49,1.95]** | **2.57 [2.02,3.27]** | **0.83 [0.76,0.92]** | **1.28 [1.04,1.58]** | **1.17 [1.02,1.36]** |
| Childhood socioeconomic class (income class) | incomeq3=5 – Ref. | - | - | - | - | - | - | - | - | - |  |  |  |
|  | incomeq3=4 | **1.29 [1.02,1.64]** | 1.38 [0.93,2.03] | 1.21 [0.99,1.49] | 0.99 [0.79,1.23] | **1.45 [1.17,1.79]** | 1.24 [0.95,1.63] | 1.25 [0.93,1.69] | 1.07 [0.87,1.31] | 1.33 [0.89,1.97] | 0.95 [0.80,1.13] | 1.34 [0.93,1.94] | 1.16 [0.92,1.45] |
|  | incomeq3=3 | **1.50 [1.15,1.95]** | 1.28 [0.88,1.87] | **1.28 [1.05,1.56]** | 1.11 [0.87,1.41] | **1.83 [1.46,2.29]** | 1.21 [0.92,1.60] | **1.63 [1.20,2.21]** | 1.09 [0.91,1.32] | **1.50 [1.00,2.24]** | 0.94 [0.82,1.09] | **1.52 [1.07,2.15]** | 1.17 [0.94,1.47] |
|  | incomeq3=2 | **1.64 [1.28,2.11]** | **1.98 [1.40,2.79]** | **1.30 [1.05,1.62]** | 1.24 [0.97,1.59] | **2.40 [1.91,3.02]** | **1.61 [1.24,2.08]** | **1.56 [1.16,2.09]** | **1.25 [1.02,1.53]** | **2.41 [1.71,3.41]** | 1.02 [0.87,1.19] | **1.88 [1.33,2.65]** | 1.22 [0.97,1.55] |
|  | incomeq3=1 | **2.00 [1.58,2.54]** | **2.33 [1.61,3.39]** | **1.37 [1.12,1.67]** | 1.09 [0.85,1.41] | **2.66 [2.12,3.35]** | **1.73 [1.33,2.25]** | **2.11 [1.60,2.78]** | **1.34 [1.07,1.69]** | **3.04 [2.15,4.29]** | 1.05 [0.90,1.23] | **3.17 [2.27,4.42]** | **1.63 [1.30,2.04]** |
| Ethnicity | White – Ref. | - | - | - | - | - | - | - | - | - |  |  |  |
|  | Ethnic minority | **0.73 [0.60,0.89]** | 0.86 [0.62,1.19] | **0.56 [0.45,0.70]** | **0.57 [0.45,0.73]** | **0.67 [0.56,0.79]** | 0.87 [0.71,1.08] | **0.44 [0.32,0.60]** | **0.66 [0.54,0.82]** | **0.64 [0.47,0.86]** | **0.63 [0.54,0.74]** | 1.07 [0.81,1.41] | **0.74 [0.59,0.91]** |

| Continuation of Supplementary Table 16 | | | | | | | | | | | | |
| --- | --- | --- | --- | --- | --- | --- | --- | --- | --- | --- | --- | --- |
|  | | **Odds ratio of outcome (95% CI)** | | | | | | | | | | |
|  |  | **(13)** | **(14)** | **(15)** | **(16)** | **(17)** | **(18)** | **(19)** | **(20)** | **(21)** | **(22)** | **(23)** |
|  |  | **Poor quality of sleep in past month** | **Overweight/obese** | **Self-rated overweight/underweight** | **Regular smoking habit** | **Frequent binge drinking** | **Recreational drug use** | **Frequent cannabis use** | **Risky sex** | **Lack of exercise** | **Attempting to change weight** | **Anti-social behaviour** |
| Main effects – Bullying | Absent – Ref. | - | - | - | - | - | - | - | - | - |  |  |
|  | Present | **1.55 [1.31,1.84]** | **1.40 [1.21,1.62]** | **1.46 [1.23,1.73]** | **1.45 [1.19,1.76]** | 0.83 [0.66,1.04] | 1.03 [0.89,1.19] | 1.25 [1.00,1.56] | **1.38 [1.13,1.68]** | **1.20 [1.03,1.41]** | **1.33 [1.13,1.57]** | **1.56 [1.35,1.81]** |
| Main effects - sexuality | Heterosexual – Ref. | - | - | - | - | - | - | - | - | - | - | **-** |
|  | bisexual | **2.10 [1.60,2.76]** | **1.36 [1.04,1.76]** | **1.71 [1.32,2.23]** | **2.32 [1.72,3.14]** | 1.18 [0.86,1.63] | **1.85 [1.49,2.31]** | 1.33 [0.93,1.90] | **1.40 [1.04,1.88]** | **1.31 [1.01,1.69]** | **1.34 [1.03,1.74]** | **1.32 [1.00,1.75]** |
|  | gay/lesb | 1.33 [0.91,1.95] | **1.48 [1.04,2.12]** | **2.02 [1.32,3.09]** | **1.59 [1.02,2.50]** | 1.06 [0.60,1.86] | 1.10 [0.74,1.62] | 1.18 [0.67,2.08] | 1.42 [0.91,2.20] | **2.34 [1.55,3.52]** | 1.15 [0.76,1.75] | 0.77 [0.45,1.32] |
| Interactions between Bullying and Sexuality | 1 # Heterosexual | - | - | - | - | - | - | - | - | - |  |  |
|  | 1 # bisexual | 0.81 [0.50,1.31] | **0.64 [0.41,1.00]** | 0.76 [0.47,1.23] | **0.48 [0.27,0.84]** | 1.06 [0.58,1.94] | 0.77 [0.49,1.21] | 1.23 [0.64,2.34] | 0.86 [0.50,1.47] | 1.05 [0.67,1.62] | **0.62 [0.39,0.99]** | 1.12 [0.67,1.84] |
|  | 1 # gay/lesb | 1.36 [0.71,2.62] | 1.15 [0.61,2.14] | 1.09 [0.52,2.27] | 0.51 [0.21,1.23] | 0.90 [0.36,2.27] | 1.40 [0.77,2.57] | 0.63 [0.23,1.67] | 1.05 [0.49,2.27] | 0.71 [0.35,1.44] | 1.72 [0.78,3.81] | 1.32 [0.59,2.93] |
| Sex of study member | Male – Ref. | - | - | - | - | - | - | - | - | - | **-** | **-** |
|  | Female | **1.24 [1.09,1.40]** | **1.31 [1.16,1.49]** | **1.33 [1.17,1.51]** | 0.94 [0.80,1.12] | 0.89 [0.75,1.04] | **0.72 [0.64,0.81]** | **0.57 [0.47,0.69]** | 0.89 [0.77,1.03] | **1.95 [1.74,2.18]** | **1.58 [1.40,1.78]** | **0.34 [0.30,0.38]** |
| Childhood socioeconomic class (income class) | incomeq3=5 – Ref. | - | - | - | - | - | - | - | - | - | - | - |
|  | incomeq3=4 | 1.11 [0.91,1.35] | **1.25 [1.01,1.55]** | 1.03 [0.86,1.23] | 1.07 [0.82,1.38] | 0.99 [0.78,1.27] | 0.90 [0.77,1.05] | 1.04 [0.80,1.35] | 1.05 [0.85,1.30] | 1.20 [0.98,1.46] | 0.93 [0.79,1.10] | 0.86 [0.72,1.04] |
|  | incomeq3=3 | **1.23 [1.01,1.51]** | **1.51 [1.26,1.82]** | **1.22 [1.04,1.44]** | **1.43 [1.09,1.89]** | 0.97 [0.78,1.22] | 0.88 [0.73,1.06] | 0.98 [0.73,1.31] | 1.00 [0.81,1.23] | **1.47 [1.19,1.82]** | 1.12 [0.95,1.33] | 0.86 [0.73,1.03] |
|  | incomeq3=2 | **1.32 [1.08,1.60]** | **1.89 [1.57,2.28]** | **1.39 [1.17,1.66]** | **2.38 [1.84,3.09]** | **0.72 [0.55,0.93]** | 0.93 [0.76,1.14] | 1.13 [0.84,1.53] | 0.98 [0.78,1.23] | **1.93 [1.58,2.36]** | 1.08 [0.90,1.31] | 0.97 [0.80,1.17] |
|  | incomeq3=1 | **1.44 [1.15,1.79]** | **2.02 [1.64,2.50]** | **1.59 [1.31,1.93]** | **3.23 [2.55,4.08]** | **0.74 [0.56,0.97]** | 0.87 [0.72,1.05] | 1.04 [0.77,1.39] | 1.03 [0.79,1.33] | **1.97 [1.62,2.41]** | 1.22 [0.99,1.50] | 0.92 [0.76,1.13] |
| Ethnicity | White – Ref. | - | - | - | - | - | - | - | - | - |  |  |
|  | Ethnic minority | 0.86 [0.73,1.02] | 1.18 [0.99,1.41] | **1.21 [1.03,1.42]** | **0.29 [0.21,0.39]** | **0.63 [0.48,0.83]** | **0.60 [0.49,0.74]** | 0.87 [0.68,1.11] | 0.83 [0.68,1.01] | 1.17 [0.98,1.40] | **1.28 [1.08,1.53]** | 1.12 [0.94,1.32] |

Text in bold: indicate 95% CIs that do not include 1.

Table Q - Associations between experience of bullying and mental health, general health, and health-risk behaviour outcomes in 8,686 adolescents from the Millennium Cohort Study (estimates are predictive margins of interactions between experiences of bullying and sexual identity from logistic regression analysis adjusted for sex, ethnicity, and childhood socioeconomic status)

|  | **Predictive margins (95% CI)** | | | | | | | | | | |
| --- | --- | --- | --- | --- | --- | --- | --- | --- | --- | --- | --- |
|  | **Psychological distress** | **SDQ-S** | | | | | **Doctor-diagnosed depression or anxiety** | **Self-harm** | **Suicidality** | **Victimization** | **Self-rated general health** |
|  |  | **Conduct problems** | **Emotional symptoms** | **Hyperactivity/inattention** | **Peer problems** | **Prosocial behaviour difficulty** |  |  |  |  |  |
|  | **(1)** | **(2)** | **(3)** | **(4)** | **(5)** | **(6)** | **(7)** | **(8)** | **(9)** | **(10)** | **(11)** |
| 0 # Heterosexual | 0.10 [0.09,0.11] | 0.04 [0.03,0.04] | 0.16 [0.15,0.18] | 0.11 [0.10,0.12] | 0.13 [0.12,0.14] | 0.10 [0.09,0.11] | 0.07 [0.06,0.08] | 0.15 [0.14,0.17] | 0.04 [0.03,0.04] | 0.39 [0.37,0.40] | 0.05 [0.04,0.05] |
| 0 # Bisexual | 0.33 [0.28,0.38] | 0.06 [0.04,0.09] | 0.34 [0.30,0.39] | 0.23 [0.19,0.27] | 0.26 [0.22,0.31] | 0.12 [0.08,0.16] | 0.21 [0.17,0.25] | 0.56 [0.50,0.61] | 0.17 [0.13,0.21] | 0.57 [0.51,0.62] | 0.08 [0.06,0.11] |
| 0 # Gay/Lesb | 0.18 [0.12,0.25] | 0.05 [0.01,0.09] | 0.32 [0.24,0.39] | 0.14 [0.09,0.20] | 0.25 [0.17,0.32] | 0.06 [0.02,0.11] | 0.19 [0.12,0.26] | 0.38 [0.29,0.47] | 0.11 [0.06,0.16] | 0.45 [0.36,0.55] | 0.10 [0.05,0.16] |
| 1 # Heterosexual | 0.17 [0.16,0.19] | 0.08 [0.06,0.09] | 0.26 [0.23,0.28] | 0.19 [0.17,0.21] | 0.30 [0.27,0.32] | 0.12 [0.10,0.13] | 0.13 [0.11,0.15] | 0.28 [0.25,0.30] | 0.10 [0.09,0.12] | 0.59 [0.56,0.62] | 0.09 [0.07,0.10] |
| 1 # Bisexual | 0.44 [0.37,0.52] | 0.15 [0.10,0.20] | 0.46 [0.38,0.54] | 0.37 [0.29,0.44] | 0.49 [0.41,0.57] | 0.12 [0.07,0.18] | 0.27 [0.21,0.34] | 0.64 [0.57,0.71] | **0.26 [0.20,0.33]** | 0.69 [0.62,0.76] | 0.15 [0.10,0.20] |
| 1 # Gay/Lesb | **0.47 [0.36,0.58]** | 0.15 [0.07,0.24] | 0.55 [0.44,0.66] | 0.40 [0.27,0.52] | 0.45 [0.33,0.56] | 0.17 [0.09,0.25] | 0.27 [0.16,0.38] | 0.65 [0.54,0.76] | 0.17 [0.08,0.25] | **0.82 [0.73,0.90]** | 0.18 [0.09,0.27] |

| Continuation of Supplementary Table 17 | | | | | | | | | | | | |
| --- | --- | --- | --- | --- | --- | --- | --- | --- | --- | --- | --- | --- |
|  | Predictive margins (95% CI) | | | | | | | | | | | |
|  | Physical/mental health condition in past year | Poor quality of sleep in past month | Overweight/obese | Self-rated overweight/underweight | Regular smoking habit | Frequent binge drinking | Recreational drug use | Frequent cannabis use | Risky sex | Lack of exercise | Attempting to change weight | Anti-social behaviour |
|  | (12) | (13) | (14) | (15) | (16) | (17) | (18) | (19) | (20) | (21) | (22) | (23) |
| 0 # Heterosexual | 0.13 [0.12,0.14] | 0.27 [0.26,0.28] | 0.25 [0.24,0.26] | 0.41 [0.40,0.42] | 0.10 [0.09,0.11] | 0.16 [0.15,0.17] | 0.30 [0.28,0.31] | 0.15 [0.13,0.16] | 0.36 [0.35,0.38] | 0.21 [0.19,0.22] | 0.57 [0.56,0.59] | 0.24 [0.22,0.25] |
| 0 # Bisexual | 0.30 [0.25,0.35] | 0.44 [0.39,0.50] | 0.32 [0.26,0.37] | 0.54 [0.49,0.60] | 0.20 [0.15,0.24] | 0.18 [0.13,0.22] | 0.43 [0.38,0.49] | 0.20 [0.15,0.25] | 0.46 [0.41,0.52] | 0.24 [0.20,0.28] | 0.65 [0.60,0.70] | 0.29 [0.24,0.34] |
| 0 # Gay/Lesb | 0.21 [0.14,0.28] | 0.34 [0.26,0.42] | 0.33 [0.25,0.40] | 0.57 [0.47,0.66] | 0.15 [0.09,0.20] | 0.18 [0.12,0.24] | 0.31 [0.23,0.39] | 0.20 [0.13,0.26] | 0.42 [0.32,0.52] | 0.38 [0.29,0.47] | 0.61 [0.52,0.70] | 0.19 [0.11,0.27] |
| 1 # Heterosexual | 0.21 [0.19,0.23] | 0.39 [0.36,0.42] | 0.32 [0.29,0.35] | 0.51 [0.48,0.54] | 0.14 [0.12,0.15] | 0.13 [0.11,0.15] | 0.30 [0.27,0.32] | 0.18 [0.16,0.20] | 0.45 [0.42,0.47] | 0.24 [0.21,0.26] | 0.65 [0.63,0.68] | 0.32 [0.29,0.34] |
| 1 # Bisexual | 0.45 [0.37,0.52] | 0.50 [0.42,0.57] | **0.29 [0.22,0.36]** | 0.57 [0.49,0.65] | **0.14 [0.09,0.19]** | 0.14 [0.08,0.20] | 0.38 [0.30,0.46] | 0.22 [0.14,0.30] | 0.49 [0.40,0.57] | 0.31 [0.24,0.38] | **0.63 [0.55,0.70]** | 0.40 [0.32,0.48] |
| 1 # Gay/Lesb | 0.36 [0.24,0.48] | 0.53 [0.41,0.64] | 0.43 [0.31,0.54] | 0.70 [0.60,0.80] | 0.11 [0.04,0.19] | 0.14 [0.06,0.21] | 0.40 [0.29,0.51] | 0.16 [0.06,0.25] | 0.51 [0.39,0.62] | 0.33 [0.21,0.45] | 0.78 [0.68,0.88] | 0.33 [0.22,0.44] |

Table R - Associations between experiences of bullying in childhood and mental health in 8,686 adolescents from the Millennium Cohort Study (estimates are from linear regression analysis adjusted for sex, ethnicity, and childhood socioeconomic status)

|  | | **Change in score [β (95% CI)]** | | | | | | | | |
| --- | --- | --- | --- | --- | --- | --- | --- | --- | --- | --- |
|  |  | **(1)** | **(2)** | **(3)** | **(4)** | **(5)** | **(6)** | **(7)** | **(8)** | **(9)** |
|  |  | **Psychological distress**  **(score/24)** | **SDQ-Subscales (score/10)** | | | | | **Mental wellbeing** | **Self-esteem**  **(score/15)** | **BMI** |
|  |  |  | **Emotional symptoms** | **Conduct problems** | **Hyperactivity/inattention** | **Peer problems** | **Prosocial behaviour difficulty** |  |  |  |
| Main effects – Bullying | Absent – Ref. | - | - | - | - | - | - | - | - | - |
|  | Present | **1.75 [1.43,2.08]** | **0.78 [0.62,0.94]** | **0.45 [0.35,0.55]** | **0.63 [0.48,0.78]** | **0.83 [0.70,0.96]** | **-0.21 [-0.32,-0.09]** | **-1.06 [-1.34,-0.78]** | **-0.81 [-1.01,-0.61]** | **0.74 [0.43,1.05]** |
| Main effects - sexuality | Heterosexual – Ref. | - | - | - | - | - | - | - | - | - |
|  | Bisexual | **4.03 [3.52,4.53]** | **1.49 [1.23,1.75]** | **0.39 [0.22,0.56]** | **0.89 [0.63,1.15]** | **0.81 [0.61,1.01]** | -0.18 [-0.38,0.01] | **-1.81 [-2.22,-1.39]** | **-1.41 [-1.76,-1.06]** | 0.18 [-0.31,0.68] |
|  | Gay/Lesbian | **2.49 [1.68,3.29]** | **1.14 [0.74,1.55]** | 0.11 [-0.21,0.42] | **0.53 [0.12,0.94]** | **0.70 [0.38,1.03]** | -0.11 [-0.38,0.16] | **-1.66 [-2.24,-1.08]** | **-1.22 [-1.79,-0.65]** | 0.65 [-0.39,1.69] |
| Interactions between Bullying and Sexuality | 1 # Hetero – Ref. | - | - | - | - | - | - | - | - | - |
|  | 1 # Bisexual | -0.37 [-1.34,0.59] | -0.21 [-0.73,0.31] | 0.25 [-0.13,0.63] | 0.23 [-0.25,0.71] | 0.09 [-0.28,0.45] | 0.15 [-0.21,0.51] | 0.30 [-0.45,1.06] | -0.18 [-0.79,0.43] | -0.15 [-1.21,0.90] |
|  | 1 # Gay/Lesb | **1.81 [0.31,3.30]** | 0.66 [-0.08,1.40] | 0.41 [-0.14,0.95] | 0.61 [-0.19,1.41] | -0.02 [-0.66,0.63] | -0.10 [-0.59,0.39] | -0.33 [-1.52,0.85] | -0.69 [-1.94,0.55] | 0.81 [-1.01,2.62] |
| Sex of study member | Male – Ref. | - | - | - | - | - | - | - | - | - |
|  | Female | **1.81 [1.60,2.02]** | **1.53 [1.41,1.66]** | **-0.30 [-0.38,-0.22]** | **-0.29 [-0.41,-0.17]** | **0.11 [0.03,0.19]** | **0.98 [0.90,1.07]** | **-1.20 [-1.39,-1.01]** | **-0.87 [-1.02,-0.72]** | **0.72 [0.48,0.96]** |
| Childhood socioeconomic class (income class) | IncomeQ3=5 – Ref. | - | - | - | - | - | - | - | - | - |
|  | IncomeQ3=4 | 0.31 [-0.03,0.66] | **0.28 [0.10,0.45]** | 0.04 [-0.07,0.15] | 0.08 [-0.11,0.26] | **0.29 [0.18,0.39]** | -0.06 [-0.20,0.07] | -0.28 [-0.57,0.00] | -0.18 [-0.38,0.02] | **0.48 [0.11,0.85]** |
|  | IncomeQ3=3 | **0.44 [0.11,0.78]** | **0.31 [0.15,0.48]** | 0.06 [-0.05,0.16] | 0.18 [-0.00,0.36] | **0.44 [0.32,0.56]** | -0.12 [-0.25,0.00] | **-0.46 [-0.77,-0.14]** | **-0.63 [-0.87,-0.39]** | **0.78 [0.44,1.13]** |
|  | IncomeQ3=2 | **0.52 [0.16,0.88]** | **0.32 [0.14,0.50]** | **0.30 [0.18,0.43]** | **0.42 [0.24,0.61]** | **0.74 [0.60,0.87]** | **-0.21 [-0.34,-0.07]** | **-0.43 [-0.73,-0.14]** | **-0.37 [-0.60,-0.14]** | **1.19 [0.79,1.59]** |
|  | IncomeQ3=1 | **0.80 [0.41,1.19]** | **0.50 [0.32,0.67]** | **0.35 [0.23,0.47]** | **0.37 [0.18,0.56]** | **0.86 [0.73,0.99]** | **-0.21 [-0.34,-0.07]** | **-0.77 [-1.10,-0.43]** | **-0.64 [-0.89,-0.40]** | **1.25 [0.82,1.69]** |
| Ethnicity | White – Ref. | - | - | - | - | - | - | - | - | - |
|  | Ethnic minority | **-0.58 [-0.87,-0.28]** | **-0.61 [-0.76,-0.46]** | -0.02 [-0.12,0.09] | **-0.54 [-0.69,-0.38]** | **-0.34 [-0.44,-0.24]** | **0.17 [0.05,0.28]** | **0.40 [0.14,0.66]** | **0.47 [0.26,0.69]** | 0.25 [-0.15,0.65] |

Warwick–Edinburgh Mental Well-being Scale (WEMWBS)

Rosenberg Self-Esteem Scale (5-item)

Table S - Associations between experiences of bullying and mental health outcomes in 8,686 adolescents from the Millennium Cohort Study (estimates are predictive margins of interactions between Experience of bullying and Sexuality from linear regression analysis adjusted for sex, ethnicity, and childhood socioeconomic status)

|  | Predictive margins [95% CI] | | | | | | | | |
| --- | --- | --- | --- | --- | --- | --- | --- | --- | --- |
|  | (1) | (2) | (3) | (4) | (5) | (6) | (7) | (8) | (9) |
|  | Psychological distress  (score/24) | SDQ-Subscales (score/10) | | | | | Mental wellbeing | Self-esteem  (score/15) | BMI |
|  |  | Emotional symptoms | Conduct problems | Hyperactivity/inattention | Peer problems | Prosocial behaviour difficulty |  |  |  |
| 0 # Heterosexual | 6.19 [6.06,6.33] | 3.06 [2.99,3.13] | 1.50 [1.46,1.54] | 3.67 [3.60,3.73] | 1.81 [1.76,1.86] | 7.93 [7.88,7.97] | 16.03 [15.91,16.14] | 10.43 [10.33,10.53] | 23.02 [22.90,23.14] |
| 0 # Bisexual | 10.22 [9.73,10.71] | 4.55 [4.29,4.82] | 1.89 [1.73,2.05] | 4.56 [4.31,4.81] | 2.61 [2.43,2.80] | 7.74 [7.54,7.94] | 14.22 [13.82,14.62] | 9.02 [8.68,9.36] | 23.20 [22.71,23.69] |
| 0 # Gay/Lesbian | 8.68 [7.90,9.46] | 4.20 [3.80,4.60] | 1.61 [1.30,1.92] | 4.20 [3.80,4.59] | 2.51 [2.19,2.83] | 7.81 [7.55,8.08] | 14.36 [13.80,14.93] | 9.21 [8.65,9.76] | 23.67 [22.63,24.71] |
| 1 # Heterosexual | 7.95 [7.68,8.22] | 3.84 [3.70,3.98] | 1.95 [1.86,2.05] | 4.30 [4.16,4.44] | 2.64 [2.52,2.75] | 7.72 [7.61,7.82] | 14.97 [14.74,15.20] | 9.62 [9.44,9.80] | 23.76 [23.48,24.04] |
| 1 # Bisexual | 11.60 [10.90,12.30] | 5.12 [4.72,5.52] | 2.59 [2.29,2.90] | 5.42 [5.06,5.78] | 3.53 [3.25,3.81] | 7.69 [7.41,7.96] | 13.47 [12.87,14.06] | 8.03 [7.57,8.49] | 23.79 [22.88,24.70] |
| 1 # Gay/Lesbian | **12.24 [11.09,13.38]** | 5.64 [5.03,6.25] | 2.47 [2.05,2.89] | 5.43 [4.75,6.12] | 3.32 [2.77,3.88] | 7.51 [7.13,7.89] | 12.97 [12.10,13.84] | 7.71 [6.71,8.70] | 25.22 [23.82,26.61] |

Warwick–Edinburgh Mental Well-being Scale (WEMWBS)

Rosenberg Self-Esteem Scale (5-item)

Table T – Information on prevalence of mental health, general health, and health-risk behaviour outcomes in 8,686 adolescents from the Millennium Cohort Study

|  | **Proportion (95% CI)** | | | | | | | | | | |
| --- | --- | --- | --- | --- | --- | --- | --- | --- | --- | --- | --- |
|  | **Psychological distress** | **SDQ-S** | | | | | **Doctor-diagnosed depression or anxiety** | **Self-harm** | **Suicidality** | **Victimization** | **Poor self-rated general health** |
|  |  | **Conduct problems** | **Emotional symptoms** | **Hyperactivity/inattention** | **Peer problems** | **Prosocial behaviour difficulty** |  |  |  |  |  |
|  | (1) | (2) | (3) | (4) | (5) | (6) | (7) | (8) | (9) | (10) | (11) |
| **Not present** | 0.86 [0.85,0.86] | 0.95 [0.94,0.95] | 0.79 [0.78,0.80] | 0.86 [0.85,0.87] | 0.81 [0.80,0.82] | 0.90 [0.89,0.90] | 0.90 [0.89,0.91] | 0.78 [0.77,0.79] | 0.93 [0.92,0.94] | 0.55 [0.54,0.56] | 0.94 [0.93,0.94] |
| **Present** | 0.14 [0.14,0.15] | 0.05 [0.05,0.06] | 0.21 [0.20,0.22] | 0.14 [0.13,0.15] | 0.19 [0.18,0.20] | 0.10 [0.10,0.11] | 0.10 [0.09,0.11] | 0.22 [0.21,0.23] | 0.07 [0.06,0.08] | 0.45 [0.44,0.46] | 0.06 [0.06,0.07] |
|  | **Physical/mental health condition in past year** | **Poor quality of sleep in past month** | **Overweight/obese** | **Self-rated overweight/underweight** | **Regular smoking habit** | **Frequent binge drinking** | **Recreational drug use** | **Frequent cannabis use** | **Risky sex** | **Lack of exercise** | **Attempting to change weight** |
|  | (12) | (13) | (14) | (15) | (16) | (17) | (18) | (19) | (20) | (21) | (22) |
| **Not present** | 0.83 [0.82,0.84] | 0.69 [0.68,0.71] | 0.73 [0.72,0.74] | 0.55 [0.54,0.57] | 0.89 [0.88,0.90] | 0.85 [0.84,0.86] | 0.69 [0.68,0.71] | 0.84 [0.83,0.86] | 0.62 [0.60,0.64] | 0.78 [0.77,0.79] | 0.40 [0.39,0.42] |
| **Present** | 0.17 [0.16,0.18] | 0.31 [0.29,0.32] | 0.27 [0.26,0.28] | 0.45 [0.43,0.46] | 0.11 [0.10,0.12] | 0.15 [0.14,0.16] | 0.31 [0.29,0.32] | 0.16 [0.14,0.17] | 0.38 [0.36,0.40] | 0.22 [0.21,0.23] | 0.60 [0.58,0.61] |

Table U – Associations between sexual identity and risk for Adverse Childhood Experiences (ACEs) in 8,686 adolescents aged 17 years from the Millennium Cohort Study (estimates are from multivariable multinomial logistic regression with adjustment for ethnicity and childhood socioeconomic position).

| **Total ACE = 0 (reference)** | | |
| --- | --- | --- |
| **Total ACE score = 1** | | |
| **Variable** | **Category** | **Relative Risk Ratio [95% CI]** |
| Main effect = Sexual identity | *Heterosexual* | *Reference* |
|  | Bisexual | 1.34 [0.93,1.93] |
|  | Gay/Lesbian | 1.32 [0.68,2.55] |
| Main effect = Sex | Female | *Reference* |
|  | Male | **1.23 [1.05,1.45]** |
| Interaction between Sexual identity and Sex | Bisexual # Male | 0.69 [0.33,1.47] |
|  | Gay/Lesbian # Male | 1.08 [0.40,2.89] |
| Main effect = Ethnicity | White | *Reference* |
|  | Ethnic minority | 1.08 [0.85,1.38] |
| Main effect = Childhood socioeconomic position (parental income quintile) | 5 (highest income) | *Reference* |
|  | 4 | 1.14 [0.88,1.47] |
|  | 3 | 1.01 [0.82,1.23] |
|  | 2 | 1.13 [0.87,1.46] |
|  | 1 (lowest income) | 0.99 [0.76,1.29] |
| **Total ACE score = 2** | | |
| **Variable** | **Category** | **Relative Risk Ratio [95% CI]** |
| Main effect = Sexual identity | *Heterosexual* | *Reference* |
|  | Bisexual | **1.46 [1.02,2.08]** |
|  | Gay/Lesbian | 1.56 [0.79,3.05] |
| Main effect = Sex | Female | *Reference* |
|  | Male | **1.36 [1.14,1.62]** |
| Interaction between Sexual identity and Sex | Bisexual # Male | 0.60 [0.27,1.31] |
|  | Gay/Lesbian # Male | 1.11 [0.41,2.97] |
| Main effect = Ethnicity | White | *Reference* |
|  | Ethnic minority | 1.22 [0.94,1.58] |
| Main effect = Childhood socioeconomic position (parental income quintile) | 5 (highest income) | *Reference* |
|  | 4 | **1.36 [1.04,1.77]** |
|  | 3 | 1.23 [0.97,1.56] |
|  | 2 | **1.70 [1.30,2.22]** |
|  | 1 (lowest income) | **1.58 [1.20,2.08]** |
| **Total ACE score ≥3** | | |
| **Variable** | **Category** | **Relative Risk Ratio [95% CI]** |
| Main effect = Sexual identity | *Heterosexual* | *Reference* |
|  | Bisexual | **1.89 [1.30,2.75]** |
|  | Gay/Lesbian | 1.90 [0.95,3.80] |
| Main effect = Sex | Female | *Reference* |
|  | Male | **1.64 [1.38,1.96]** |
| Interaction between Sexual identity and Sex | Bisexual # Male | 0.89 [0.44,1.79] |
|  | Gay/Lesbian # Male | 1.24 [0.45,3.43] |
| Main effect = Ethnicity | White | *Reference* |
|  | Ethnic minority | 1.12 [0.87,1.45] |
| Main effect = Childhood socioeconomic position (parental income quintile) | 5 (highest income) | *Reference* |
|  | 4 | 1.27 [0.94,1.71] |
|  | 3 | **1.68 [1.28,2.20]** |
|  | 2 | **2.98 [2.22,4.01]** |
|  | 1 (lowest income) | **3.20 [2.43,4.20]** |

Estimates in bold indictate statistical significance i.e. 95% CI does not include 1.

**Table V – Associations between Sexual identity and risk for Adverse Childhood Experiences (ACEs) in 8,686 adolescents aged 17 years from the Millennium Cohort Study (estimates are predictive margins of interactions between Sexual identity and Sex from multivariable multinomial logistic regression with adjustment for ethnicity and childhood socioeconomic position)**

| **Total ACE = 0** | |
| --- | --- |
| **Subgroup** | Predictive margins (Proportion of subgroup with 0 ACE [95% CI]) |
| **Heterosexual # Male** | 0.18 [0.16,0.19] |
| **Heterosexual # Female** | 0.23 [0.21,0.25] |
| **Bisexual # Male** | 0.15 [0.08,0.23] |
| **Bisexual # Female** | 0.16 [0.13,0.19] |
| **Gay/Lesbian # Male** | 0.11 [0.04,0.17] |
| **Gay/Lesbian # Female** | 0.15 [0.08,0.22] |
| **Total ACE score = 1** | |
| **Subgroup** | Predictive margins (Proportion of subgroup with 1 ACE [95% CI]) |
| **Heterosexual # Male** | 0.33 [0.31,0.35] |
| **Heterosexual # Female** | 0.34 [0.32,0.36] |
| **Bisexual # Male** | 0.24 [0.17,0.31] |
| **Bisexual # Female** | 0.36 [0.31,0.41] |
| **Gay/Lesbian # Male** | 0.27 [0.18,0.37] |
| **Gay/Lesbian # Female** | 0.33 [0.25,0.42] |
| **Total ACE score = 2** | |
| **Subgroup** | Predictive margins (Proportion of subgroup with 2 ACEs [95% CI]) |
| **Heterosexual # Male** | 0.27 [0.25,0.28] |
| **Heterosexual # Female** | 0.26 [0.24,0.27] |
| **Bisexual # Male** | 0.29 [0.20,0.37] |
| **Bisexual # Female** | 0.24 [0.20,0.28] |
| **Gay/Lesbian # Male** | 0.31 [0.23,0.39] |
| **Gay/Lesbian # Female** | 0.28 [0.19,0.37] |
| **Total ACE score = ≥3** | |
| **Subgroup** | Predictive margins (Proportion of subgroup with ≥3 ACEs [95% CI]) |
| **Heterosexual # Male** | 0.22 [0.21,0.24] |
| **Heterosexual # Female** | 0.18 [0.16,0.19] |
| **Bisexual # Male** | 0.32 [0.24,0.40] |
| **Bisexual # Female** | 0.24 [0.20,0.29] |
| **Gay/Lesbian # Male** | 0.31 [0.22,0.41] |
| **Gay/Lesbian # Female** | 0.24 [0.16,0.32] |

Table W - Comparison of the distribution of Adverse Childhood Experiences (ACEs) in 8,686 adolescents aged 17 years from the Millennium Cohort Study from pre- and post-imputation data

|  | | **Adverse Childhood Experience (ACE)** | | | | | | | |
| --- | --- | --- | --- | --- | --- | --- | --- | --- | --- |
|  |  | **Maternal drug use** | **Maternal psychological distress** | **Domestic violence against mother** | **Maternal physical punishment** | **Maternal harsh parenting** | **Parental separation/divorce** | **Maternal problematic drinking** | **Bullying** |
| **Complete case data** | **Absent (%)** | 94.1 | 90.2 | 93.5 | 76.1 | 43.5 | 83.5 | 96.2 | 78.6 |
|  | **Present (%)** | 5.9 | 9.8 | 6.5 | 23.9 | 56.5 | 16.5 | 3.8 | 21.4 |
|  | **Missing (n/8,686)** | 2,729 | 3,180 | 4,930 | 2,539 | 3,141 | 2,300 | 4,933 | 1,265 |
| **Imputed data**  **(% [95% CI])** | **Absent** | 93.0 [92.4, 93.6] | 85.9 [85.1, 86.7] | 86.7 [0.857,0.877] | 74.5 [73.4, 75.5] | 45.1 [44.0, 46.3] | 81.9 [81.1, 82.8] | 92.2 [91.3, 93.1] | 78.3 [77.4, 79.2] |
|  | **Present** | 7.0 [6.4, 7.6] | 14.1 [13.3, 14.9] | 13.3 [0.123,0.143] | 25.5 [24.5, 26.6] | 54.9 [53.7, 56.0] | 18.1 [17.2, 18.9] | 7.8 [6.9, 8.7] | 21.7 [20.8, 22.6] |
